# 1,3-Dianionic annulation of ketones with ketene dithioacetal: a modified route to 3-aryl/cyclopropyl-5-thiomethyl-phenols and 1-(methylthio)-9,10-dihydrophenanthren-3-ols†

**DOI:** 10.1039/d3ra05421g

**Published:** 2023-11-22

**Authors:** Ranjay Shaw, Prasoon Prakash, Ismail Althagafi, Nand Gopal Giri, Ramendra Pratap

**Affiliations:** a Department of Chemistry, GLA University Mathura UP 281406 India; b Department of Kinesiology and Educational Psychology, Washington State University Pullman WA 99164 USA; c Department of Chemistry, Faculty of Science, Umm Al-Qura University Makkah 21955 Saudi Arabia; d Department of Chemistry, Shivaji College, University of Delhi Raja Garden New Delhi 110027 India; e Department of Chemistry, University of Delhi Delhi 110007 India ramendrapratap@gmail.com

## Abstract

A simple and efficient base-mediated [3 + 3] cyclization of 1,3-dianionic ketones with 3,3-bis(methylthio)-1-arylprop-2-en-1-ones was developed to afford 3-hydroxy-biaryls, hydroxy-xylenes, and hydroxy-teraryls. Various tri- and tetra-substituted phenols were prepared from different symmetric and asymmetric ketones. The reaction of 2-(bis(methylthio)methylene)-3,4-dihydronaphthalen-1(2*H*)-ones with different ketones provided 1-(methylthio)-9,10-dihydrophenanthren-3-ols in very good yield. The scope of the reaction was further extended by the synthesis of cyclopropyl-functionalized phenols. One of the compounds was crystallized, and its structure was confirmed using the single-crystal X-ray approach.

## Abstract 2

A simple and efficient approach was developed *via* the 1,3-dianionic annulation of acetone to afford multifunctional phenols.

## Graphical abstract

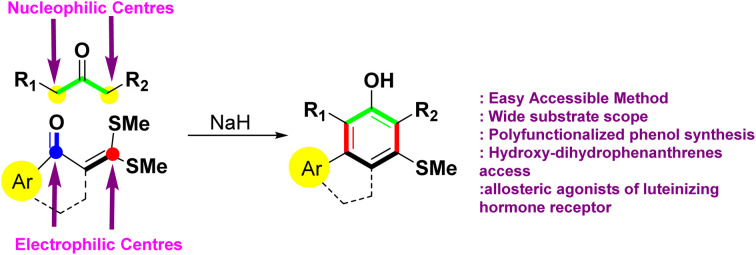

## Introduction

**Fig. 1:**
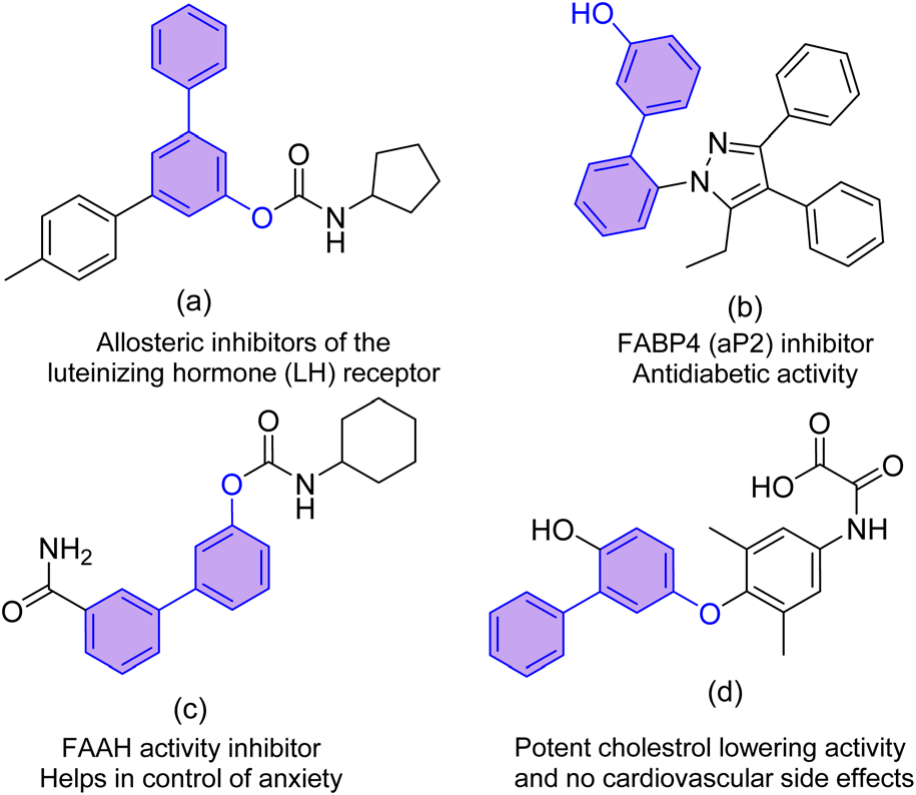
Some useful compounds with a 3-hydroxybiaryl scaffold.

Hydroxy-biaryls are the core structural motif of various natural and synthetically important medicinal compounds as a whole or substructure.^1^ Among them, 3-hydroxy-biaryls and their derivatives exhibit excellent biological activity and are extensively used in the medicinal, agrochemical, biotech, and synthetic polymer industries.^2^ 4-Methyl-[1,1′:3′,1′′-terphenyl]-5′-yl cyclopentyl carbamate (Fig. 1a) is an allosteric inhibitor of luteinizing hormone (LH) receptor, which plays a vital role in fertility and certain cancer (mainly ovarian).^3^ Similarly, 2′-(5-ethyl-3,4-diphenyl-1*H*-pyrazol-1-yl)-[1,1′-biphenyl]-3-ol (Fig. 1b) and 3′-carbamoyl-[1,1′-biphenyl]-3-yl cyclohexylcarbamate (Fig. 1c) possess promising antidiabetic and anxiolytic properties, respectively.^4^ Moreover, 2-((4-((6-hydroxy-[1,1′-biphenyl]-3-yl)oxy)-3,5-dimethylphenyl)amino)-2-oxoacetic acid (Fig. 1d) have shown very good response in lowering cholesterol level in the body, and it does not have any cardiovascular side effect unlike triiodothyronine (T3, often used to control body metabolism).^5^ Along with these, derivatives of 3-phenylphenol were also known for their good estrogenic, antibacterial, and fungistatic activities.^6^

However, the construction of *meta*-substituted phenols is a challenging task for organic chemists and hence has been less explored. Electrophilic substitution is not suitable for the functionalization of phenol at the *meta* position. However, phenols do not undergo nucleophilic substitution reaction because their benzene ring has a high π-electron density owing to the +R effect of the –OH group. Unlike the synthesis of *ortho*- and *para*-hydroxybiaryls, reports on the synthesis of *meta*-aryl phenols are limited. Among them, the [3 + 3] and [4 + 2] cyclizations of aliphatic skeletons are the most promising ways to construct it. Eichinger *et al.* reported a [3 + 3] cyclization strategy to synthesize 3,5-disubstituted aryl/alkyl phenols from chalcones and 1-acetonylpyridinium.^7^ Later on, it was observed that the use of 1-(benzotriazol-1-yl)propan-2-one as a reagent instead of 1-acetonylpyridinium chloride enhances the overall yield of the reaction.^8^ These reactions produced pyridine and benzotriazole as side products. Qian *et al.* disclosed a [3 + 3] annulation pathway for the synthesis of 3,5-disubstituted phenols from α,β-unsaturated ketones, and α-fluoro-β-ketoesters.^9^

Meanwhile, the Liebeskind group developed a [4 + 2] cyclization strategy to construct 3,5-disubstituted phenols from a cobalt complex of conjugated ketenes and alkynes.^10^ The Junjappa group reported the synthesis of various substituted phenols and 9,10-dihydrophenanthrenes from the [4 + 2] cycloaromatization of methyl ketene dithioacetal and ketones.^11^ Although, ketene dithioacetals are excellent electrophilic precursors,^12^ alkyl ketene dithioacetals are tedious to prepare and afford very low yield owing to self-substitution under basic conditions. Further, the ketone substrates are generally limited to cyclic ketones, which restricts the scope of substrates in the reaction. Therefore, the development of new approaches for the synthesis of functionalized hydroxy arenes continues to be of great interest among synthetic chemists. Herein, we reported an efficient and cost-effective base-mediated [3 + 3] cyclization of simple ketones with diverse α-aroyl ketene dithioacetals for the synthesis of various 3-hydroxy biaryls, teraryl, and tetraaryl systems and further evaluated their biological activity against luteinizing hormone/choriogonadotropin receptor (LHCGR) using a computational approach.

## Results and discussion

At the outset, the reaction of 3,3-bis(methylthio)-1-phenylprop-2-en-1-one (1a) and acetone (2) was performed using KOH in DMF or DMSO as solvent at 25 °C for 12 hours, and no product was observed (Table 1, entries 1 and 2). An elevated reaction temperature of up to 50 °C was also not able to furnish any product in the reaction (Table 1, entry 3). Then, stronger bases NaH and NaNH_2_ were inspected for the reaction in DMSO at 50 °C, and 5-(methylthio)-[1,1′-biphenyl]-3-ol (3a) was obtained in 64% and 57%, respectively (Table 1, entries 4 and 5). Further, several combinations of base and solvent were examined for the optimization of the reaction condition, and NaH in DMF at 50 °C was observed as the best reaction condition, which provides a 72% yield of 5-(methylthio)-[1,1′-biphenyl]-3-ol (3a) in 8 hours (Table 1, entry 6). Alkoxide bases, such as *t*-BuOK and NaOEt in DMF, also provided product 3a in good yield. However, the less polar solvents, THF and dioxane, resulted in an inferior yield of the 3a. The structure of product 3a was confirmed by ^1^H NMR, ^13^C NMR, and mass spectrometry techniques.

**Table 1:** Optimization of reaction condition for the synthesis of 3a^a^

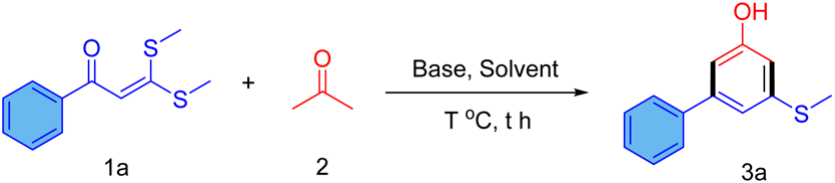					
Entry	Base	Solvent	Temp. (°C)	Time (h)	% Yield^b^
1	KOH	DMF	25	12	—
2	KOH	DMSO	25	12	—
3	KOH	DMSO	50	12	—
4	NaH	DMSO	50	8	64
5	NaNH_2_	DMSO	50	8	57
**6**	**NaH**	**DMF**	**50**	**8**	**72**
7	NaH	DMF	80	6	65
8	NaH	DMF	25	12	—
9	*t*-BuOK	DMF	50	10	63
10	NaOEt	DMF	50	10	57
11	NaH	THF	Reflux	12	28
12	NaH	Dioxane	90	12	33

a All the reactions were performed using 0.3 mmol of 3,3-bis(methylthio)-1-phenylprop-2-en-1-one (1a), 0.5 mmol of acetone (2), and 0.75 mmol of NaH in 1.5 mL of DMF.

b Yield of the isolated product after column chromatography.

**Scheme 1:**
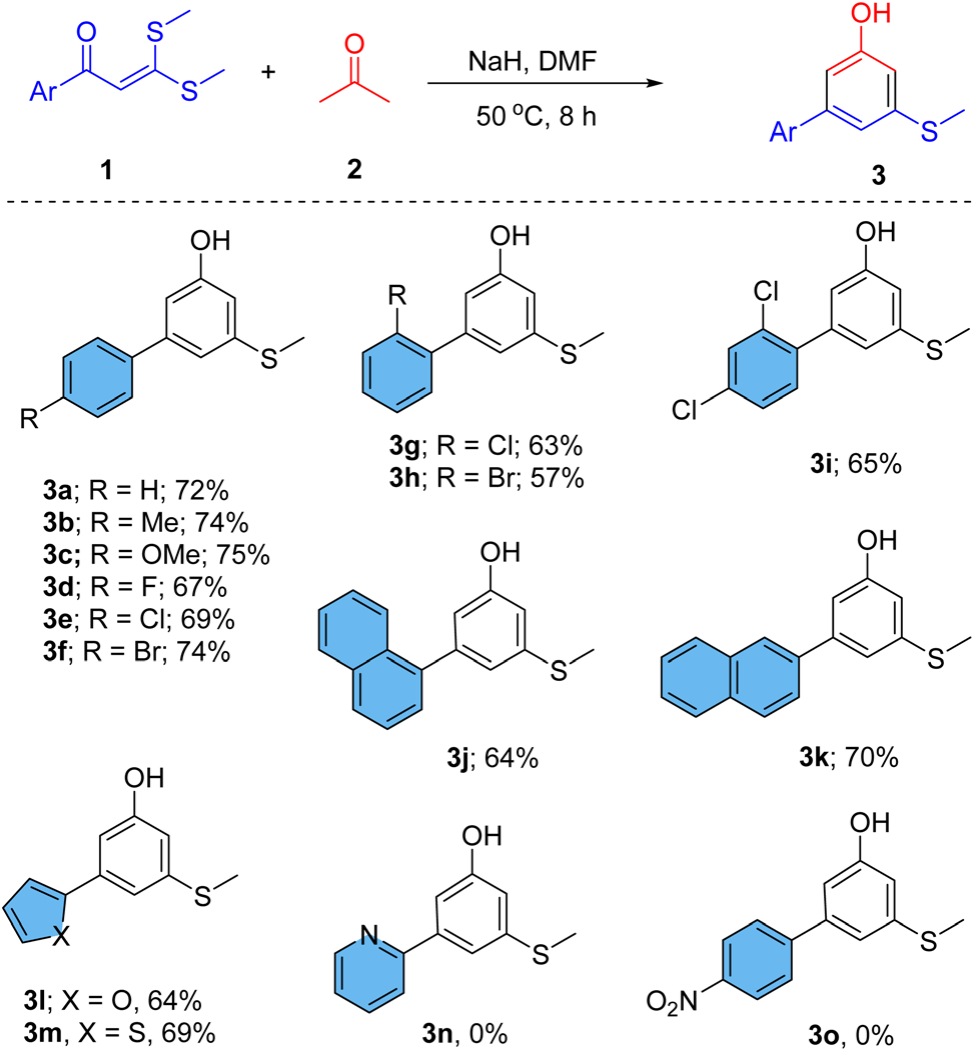
Synthesis of 5-aryl-3-methylthio-phenols (3).

Several 3-aryl-5-methylthio-phenols (3a–m) were synthesized in 63–76% yield under the optimized reaction conditions (Scheme 1). The reaction showed great tolerance to halides (3d–i), which is the prime advantage of this method over metal-catalyzed coupling strategies for the synthesis of halogenated hydroxy-biaryls. Electron donating substituents, such as –OMe and halogens, at the *para* position of the aryl ring of ketene dithioacetal, lead to a slight increase in the yield of the reaction (3c–f). When electron-withdrawing pyridyl or 4-nitrophenyl rings are attached to the carbonyl carbon of the ketene dithioacetals, the reaction does not yield products 3n and 3o as expected. However, *ortho* substituents on the aryl ring cause a steric interference for nucleophilic attack on the carbonyl carbon, and a lower yield of the product was obtained (3g–h). In addition, differently substituted arylated phenols have been synthesized in good yields, such as dichlorophenyl (3i, 65%) naphthalen-1-yl (3j, 64%), naphthalen-2-yl (3k, 70%) and heteroaryl-substituted phenols (3l–m). No specific trend was observed in the yield of the products depending on the nature of the aryl group.

**Scheme 2:**
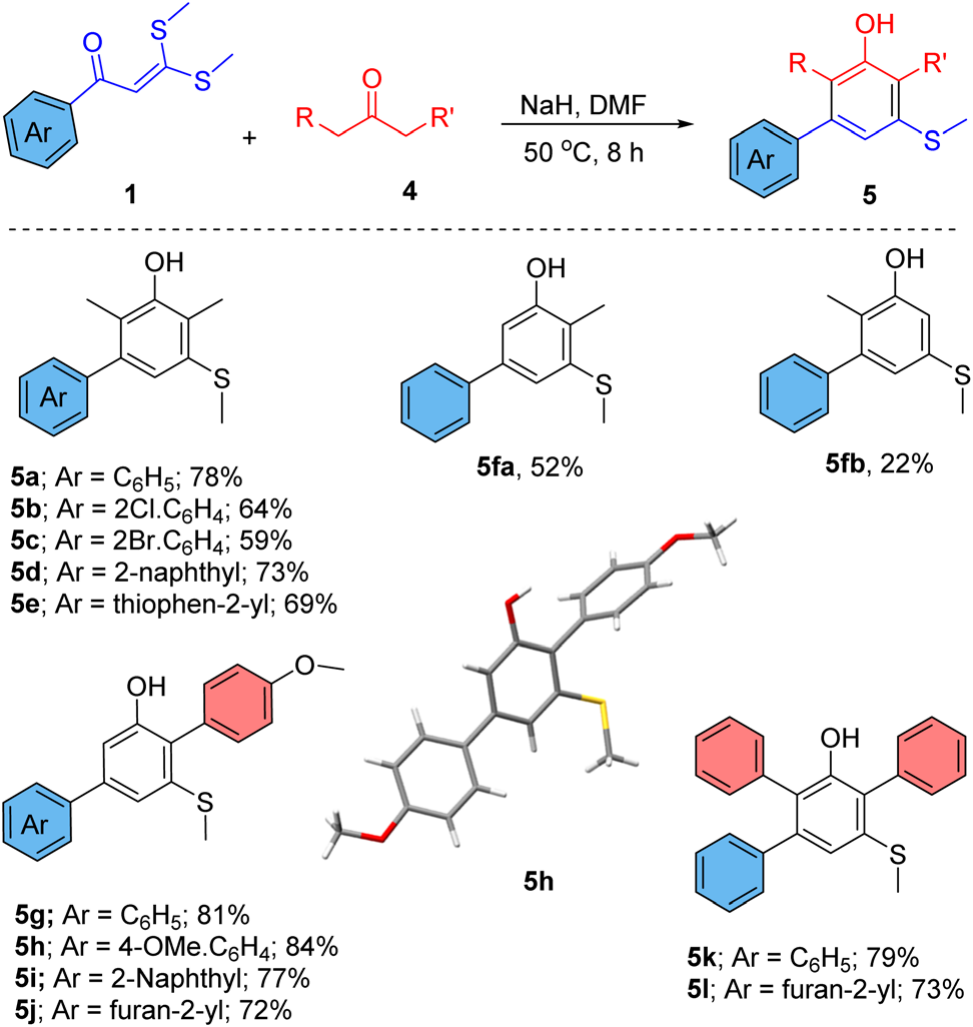
Scope of various ketones for the synthesis of functionalized phenols.

The scope of this reaction was further explored using different ketones (Scheme 2). When 3-pentanone was treated with 1a, substituted 2,6-xylenols (5a–e) were obtained as a product. Xylenols are often used in the synthesis of natural products and medicinally important compounds.^2*c*,13^ Apart from these, they are also used in the preparation of polymers, pigments, cosmetics, and antioxidants.^2*c*,14^ Reaction of an asymmetric ketone 2-butanone with 1a produces a mixture of 4-methyl-5-(methylthio)-[1,1′-biphenyl]-3-ol (5fa, major, 52%) and 2-methyl-5-(methylthio)-[1,1′-biphenyl]-3-ol (5fb, minor, 22%). To achieve complete selectivity for product 5fb, bulky bases, such as *t*-BuOK and KHMDS, were used instead of NaH, but again a mixture of 5fa and 5fb was obtained. Curiously, 1-(4-methoxyphenyl)-propane-2-one was tried as a ketonic reagent with 1a in the reaction, and 4-methoxy-6′-(methylthio)-[1,1′:4′,1′′-terphenyl]-2′-ol (5g) was selectively obtained in very good yield even with NaH. The nature of aryl substitution in propan-2-one plays an important role in the selectivity of the reaction by producing regioselective enolates from 1-(4-methoxyphenyl)propan-2-one. Various *ortho*-aryl phenols (5g–j) were prepared in very good yield under the same reaction conditions. Similarly, the reaction of 1,3-diphenylacetone provided teraryl 5-(methylthio)phenols (5k–l). *o*-Aryl-phenols are very good precursors for the synthesis of dibenzofurans.^15^ Hence, intramolecular cyclizations of such *ortho*-aryl phenols in this reaction could furnish directly functionalized dibenzofurans. Further, we confirmed the structure of compound 5h by applying single crystal X-ray.

**Scheme 3:**
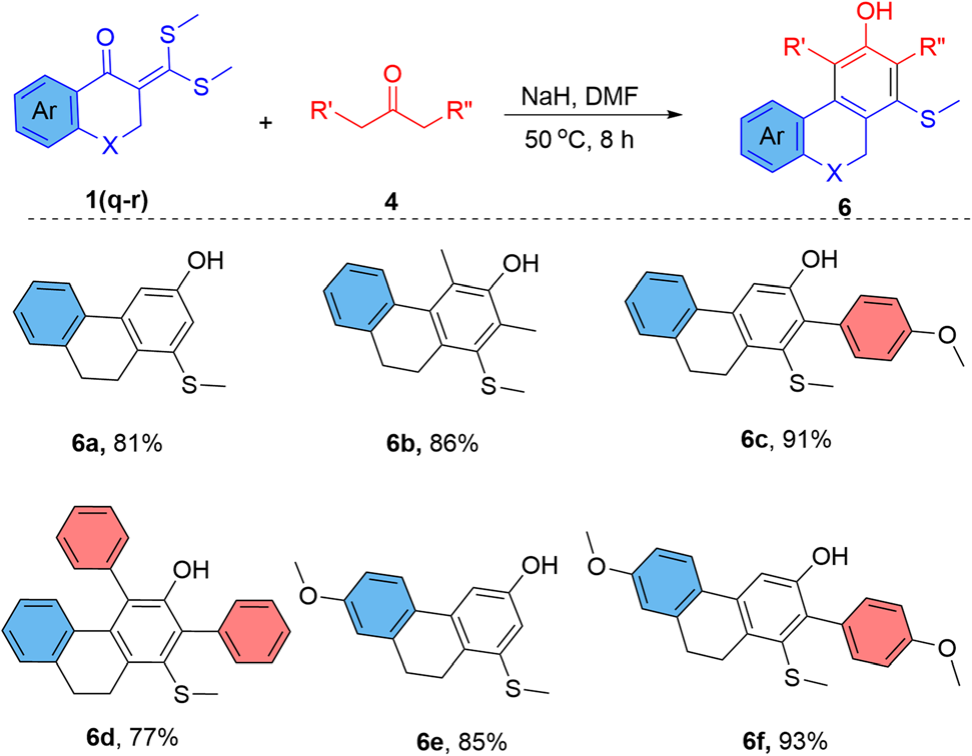
Synthesis of bridged hydroxy-biaryls (9,10-dihydrophenanthren-3-ol).

Inspired by these results, the ketene dithioacetals of cyclic ketones were investigated for the reaction. The reaction of 2-(bis(methylthio)methylene)-3,4-dihydronaphthalen-1(2*H*)-ones (1q–r) with alkyl ketones provides polycyclic phenols in very good yield (Scheme 3). Previous approaches for the synthesis of 5,6-dihydrophenanthren-3-ols or 3-hydroxy-phenanthrenes involve thermolysis of 4-phenethylphenols^16^ or aromatization of tetrahydrophenanthren-3(2*H*)-ones^17^ or Diels–Alder cyclization between nitronaphthalenes and Danishefsky's diene.^18^ These synthetic methods require very high reaction temperatures and provide a low yield of products. We have prepared several 1-(methylthio)-9,10-dihydrophenanthren-3-ols (6a–f) in 81–93% yield using a simple base-mediated cyclization approach. The restricted spatial geometry of the ketene dithioacetals of cyclic ketones favours the reaction and provides a better product yield than the ketene dithioacetals of acyclic ketones.

**Scheme 4:**
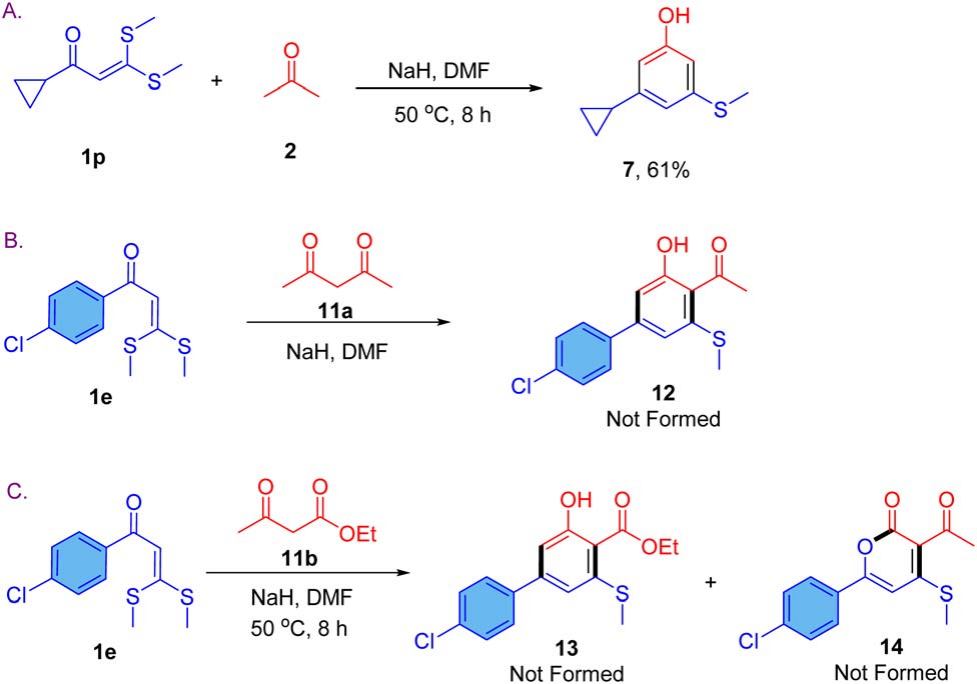
Scope and limitation of the reaction; (A) use of 1-cyclopropyl-3,3-bis(methylthio)prop-2-en-1-one as precursor; (B) reaction with acetylacetone; (C) reaction with ethyl acetoacetate.

To evaluate the effectiveness of the reaction involving α-alkanoyl ketene dithioacetals, we conducted a reaction between 1-cyclopropyl-3,3-bis(methylthio)prop-2-en-1-one (1p) and acetone under optimized conditions, resulting in the formation of product 7 in moderate yields (Scheme 4A). We also intended to explore the cyclization of α-aroyl ketene dithioacetals with acetylacetone and ethyl acetoacetate as dianion sources, but the reaction did not progress under either the optimized or elevated temperature conditions of 70 °C (Scheme 4B and C).

**Scheme 5:**
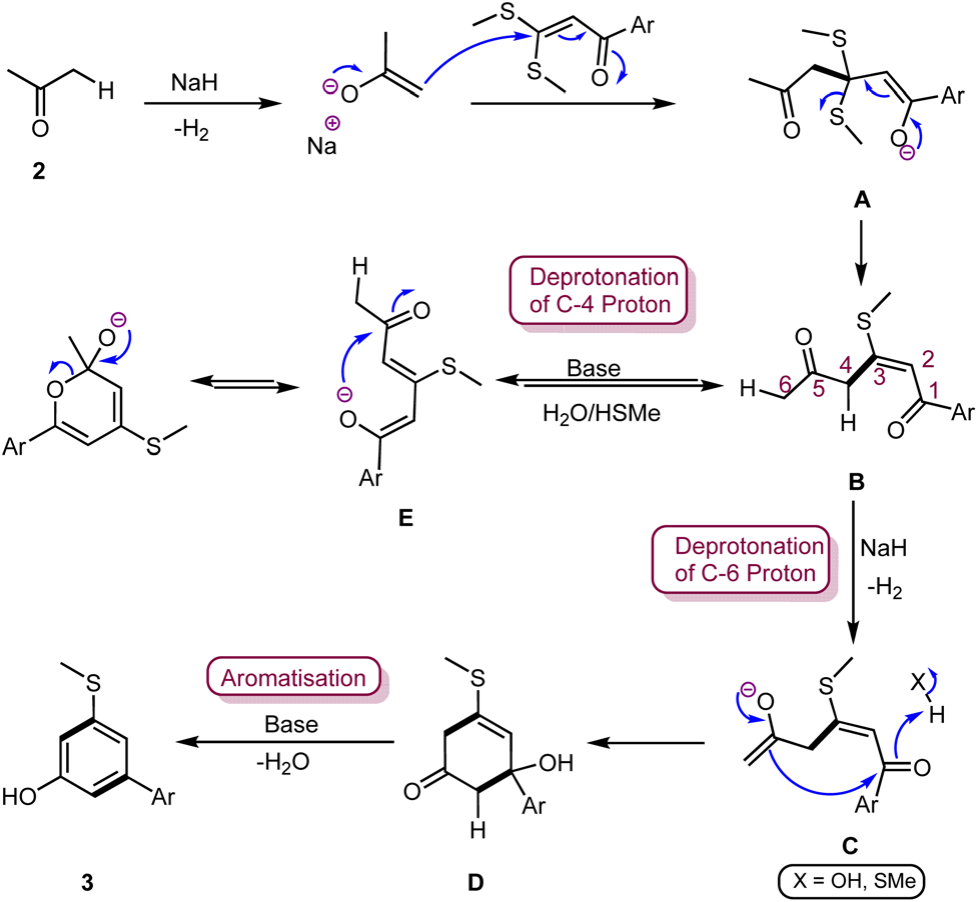
Plausible mechanism of the synthesis of functionalized phenols.

Based on these observations, a reaction mechanism was proposed, as depicted in Scheme 5. The reaction is initiated by the Michael addition of enolate of ketone (2) to ketene dithioacetal, followed by the elimination of –SMe to produce a substitution product 3-(methylthio)-1-arylhex-2-ene-1,5-dione (B). Under basic reaction conditions, intermediate B can undergo deprotonation from either C-4 or C-6 carbon center. Deprotonation from C-4 of intermediate B is more feasible than deprotonation from C-6; hence, the formation of enolate E cannot be ignored. However, enolate E does not lead to any stable product and reverts to intermediate B by absorbing one proton from the reaction mixture. In the presence of a weaker base or at room temperature, intermediate B probably exhibits only C-4 deprotonation. However, in the presence of a stronger base, such as NaH, intermediate B undergoes C-6 deprotonation to generate an enolate C, which undergoes 1,6-intramolecular cyclization to produce cyclic intermediate D. Subsequent condensation, followed by aromatization of intermediate D in the base environment, results in the phenolic product 3.

The synthesis of products 5, 6 and 7 also follows a similar reaction pathway. In this reaction, ketones contribute two nucleophilic carbon centres as dianions, while ketene dithioacetal acts as a 1,3-dielectrophilic partner to construct a six-membered cyclic ring *via* a [3 + 3] cycloaromatization reaction.

## Experimental section

### General

Commercially available reagents and solvents from Alfa Aesar, Spectrochem, Sigma-Aldrich, Fisher Scientific and TCI Chemicals were used without further purification. ^1^H and ^13^C NMR spectra were recorded using a 400 MHz and 100 MHz NMR spectrometer (Jeol and Bruker Instrument) respectively, and CDCl_3_ (from Eurisotop) was used as the solvent. Chemical shifts for all the compounds are reported in parts per million (ppm) shifts (*δ*-value). One singlet at *δ* 7.26 ppm of ^1^H and a triplet at 77.00 ppm of ^13^C NMR for CDCl_3_ was taken as an internal standard. Signal patterns are mentioned as s, singlet; d, doublet; dd, double doublet; t, triplet; q, quartet; m, multiplet; bs, broad singlet and bm, broad multiplet. The coupling constant (*J*) for protons is given in hertz (Hz). Infrared (IR) spectra were recorded using a PerkinElmer AX-1 spectrophotometer and reported in wave number (cm^−1^). HRMS is reported for the peak of (M + H)^+^ using an Agilent G6530AA (LC-HRMS-Q-TOF) spectrometer. Reagent-grade solvents were used for extraction and chromatography. The yield of the product was reported as chromatographically isolated pure materials.

### General protocol for the synthesis of 5-aryl-3-methylthio-phenols (3a–m)

To a nitrogen-flushed vial, α-aroyl ketene dithioacetal (1, 0.3 mmol), acetone (2, 0.5 mmol) and NaH (0.75 mmol) were mixed, and 1.5 mL of DMF was added. The mixture was stirred at 50 °C, and the reaction was observed by TLC in regular time intervals. On completion of the reaction, the crude was neutralized by 10% HCl and then extracted with ethyl acetate and water three times (25 mL × 3). The organic layer was collected, dried, and purified by column chromatography using 10% of ethyl acetate in hexane.

### 3a: 5-(methylthio)-[1,1′-biphenyl]-3-ol

Yield: 72%; 0.30*R*_f_ (10% ethylacetate in hexane), red viscous liquid; ^1^H-NMR (400 MHz, CDCl_3_) *δ* 7.54 (d, *J* = 7.0 Hz, 2H, Ar–H), 7.42 (t, *J* = 7.4 Hz, 2H, Ar–H), 7.35 (t, *J* = 7.2 Hz, 1H, Ar–H), 7.04 (t, *J* = 1.5 Hz, 1H, Ar–H), 6.83 (t, *J* = 1.8 Hz, 1H, Ar–H), 6.74 (t, *J* = 1.9 Hz, 1H, Ar–H), 5.81 (b s, 1H, –OH), 2.50 (s, 3H, –SCH_3_); ^13^C-NMR (100 MHz, CDCl_3_) *δ* 156.3, 143.3, 140.4, 140.4, 128.7, 127.7, 127.1, 117.6, 111.9, 111.2, 15.6; IR (cm^−1^): 3351 (broad), 3055, 2926, 1705, 1585, 1429, 1190, 926, 818, 697; HRMS (*m*/*z*): [M + H]^+^ calculated for (C_13_H_13_OS)^+^: 217.0682; found: 217.0689.

### 3b: 4′-methyl-5-(methylthio)-[1,1′-biphenyl]-3-ol

Yield: 74%; 0.30*R*_f_ (10% ethylacetate in hexane), red viscous liquid; ^1^H-NMR (400 MHz, CDCl_3_) *δ* 7.44 (d, *J* = 8.1 Hz, 2H, Ar–H), 7.23 (d, *J* = 7.8 Hz, 2H, Ar–H), 7.02 (t, *J* = 1.4 Hz, 1H, Ar–H), 6.82 (t, *J* = 1.8 Hz, 1H, Ar–H), 6.72 (t, *J* = 1.9 Hz, 1H, Ar–H), 5.68 (b s, 1H, –OH), 2.50 (s, 3H, –SCH_3_), 2.39 (s, 3H, –CH_3_); ^13^C-NMR (100 MHz, CDCl_3_) *δ* 156.3, 143.2, 140.3, 137.5, 130.2, 129.4, 126.9, 117.4, 111.6, 111.0, 21.1, 15.6; IR (cm^−1^): 3341 (broad), 3052, 2957, 1695, 1579, 1424, 1193, 926, 823, 701; HRMS (*m*/*z*): [M + H]^+^ calculated for (C_14_H_15_OS)^+^: 231.0838; found: 231.0851.

### 3c: 4′-methoxy-5-(methylthio)-[1,1′-biphenyl]-3-ol

Yield: 75%; 0.25*R*_f_ (10% ethylacetate in hexane), red viscous liquid; ^1^H-NMR (400 MHz, CDCl_3_) *δ* 7.45 (d, *J* = 8.8 Hz, 2H, Ar–H), 6.99 (t, *J* = 1.5 Hz, 1H, Ar–H), 6.94 (d, *J* = 8.9 Hz, 2H, Ar–H), 6.79 (t, *J* = 1.9 Hz, 1H, Ar–H), 6.70 (t, *J* = 1.9 Hz, 1H, Ar–H), 6.26 (s, 1H, –OH), 3.84 (s, 3H, –OCH_3_), 2.48 (s, 3H, –SCH_3_); ^13^C-NMR (100 MHz, CDCl_3_) *δ* 159.2, 156.3, 142.7, 140.2, 132.8, 128.1, 117.1, 114.1, 111.3, 110.7, 55.3, 15.5; IR (cm^−1^): 3363 (broad), 3054, 2949, 1698, 1576, 1423, 1190, 898, 830, 702; HRMS (*m*/*z*): [M + H]^+^ calculated for (C_14_H_15_O_2_S)^+^: 247.0787; found: 217.0796.

### 3d: 4′-fluoro-5-(methylthio)-[1,1′-biphenyl]-3-ol

Yield: 67%; 0.20*R*_f_ (10% ethylacetate in hexane), brown sticky solid; ^1^H-NMR (400 MHz, CDCl_3_) *δ* 7.49 (dd, *J* = 8.7, 5.4 Hz, 2H, Ar–H), 7.13–7.09 (m, 2H, Ar–H), 6.98 (s, 1H, Ar–H), 6.74 (d, *J* = 16.4 Hz, 2H, Ar–H), 5.23 (s, 1H, –OH), 2.50 (s, 3H, –SCH_3_); ^13^C-NMR (100 MHz, CDCl_3_) *δ* 163.8 & 161.4 (*J* = 245.4 Hz), 156.2, 142.3, 140.6, 128.7 & 128.6 (*J* = 7.7 Hz), 127.4, 117.5, 115.7 & 115.5 (*J* = 22.0 Hz), 111.7, 111.0, 15.5; IR (cm^−1^): 3373 (broad), 3054, 2923, 1699, 1580, 1430, 1199, 926, 822, 734; HRMS (*m*/*z*): [M + H]^+^ calculated for (C_13_H_12_FOS)^+^: 235.0587; found: 235.0590.

### 3e: 4′-chloro-5-(methylthio)-[1,1′-biphenyl]-3-ol

Yield: 69%; 0.25*R*_f_ (10% ethylacetate in hexane), red viscous liquid; ^1^H-NMR (400 MHz, CDCl_3_) *δ* 7.54 (d, *J* = 8.7 Hz, 2H, Ar–H), 7.40 (d, *J* = 8.7 Hz, 2H, Ar–H), 6.98 (t, *J* = 1.5 Hz, 1H, Ar–H), 6.77–6.76 (m, 1H, Ar–H), 6.74–6.73 (m, 1H, Ar–H), 5.29 (s, 1H, –OH), 2.50 (s, 3H, –SCH_3_); ^13^C-NMR (100 MHz, CDCl_3_) *δ* 156.3, 142.1, 140.8, 139.3, 131.9, 128.7, 122.0, 117.4, 112.2, 110.9, 15.6; IR (cm^−1^): 3349 (broad), 3049, 2924, 1691, 1577, 1429, 1189, 927, 821, 697; HRMS (*m*/*z*): [M + H]^+^ calculated for (C_13_H_12_ClOS)^+^: 251.0292; found: 251.0304.

### 3f: 4′-bromo-5-(methylthio)-[1,1′-biphenyl]-3-ol

Yield: 74%; 0.25*R*_f_ (10% ethylacetate in hexane), red viscous liquid; ^1^H-NMR (400 MHz, CDCl_3_) *δ* 7.46 (d, *J* = 8.7 Hz, 2H, Ar–H), 7.38 (d, *J* = 8.4 Hz, 2H, Ar–H), 6.98 (s, 1H, Ar–H), 6.77 (t, *J* = 1.6 Hz, 1H, Ar–H), 6.73 (t, *J* = 1.7 Hz, 1H, Ar–H), 5.43 (s, 1H, –OH), 2.50 (s, 3H, –SCH_3_); ^13^C-NMR (100 MHz, CDCl_3_) *δ* 156.3, 142.0, 140.8, 138.8, 133.7, 128.9, 128.3, 117.4, 112.1, 111.0, 15.6; IR (cm^−1^): 3365 (broad), 3053, 2954, 1692, 1578, 1431, 1190, 929, 828, 699; HRMS (*m*/*z*): [M + H]^+^ calculated for (C_13_H_12_BrOS)^+^: 294.9787/294.9766; found: 294.9795/294.9772.

### 3g: 2′-chloro-5-(methylthio)-[1,1′-biphenyl]-3-ol

Yield: 63%; 0.25*R*_f_ (10% ethylacetate in hexane), red viscous liquid; ^1^H-NMR (400 MHz, CDCl_3_) *δ* 7.47–7.44 (m, 1H, Ar–H), 7.31–7.27 (m, 3H, Ar–H), 6.87 (s, 1H, Ar–H), 6.77 (d, *J* = 2.2 Hz, 1H, Ar–H), 6.68 (t, *J* = 1.0 Hz, 1H, Ar–H), 5.34 (s, 1H, –OH), 2.49 (s, 3H, –SCH_3_); ^13^C-NMR (100 MHz, CDCl_3_) *δ* 155.5, 141.2, 139.9, 139.7, 132.3, 131.1, 129.9, 128.8, 126.8, 119.8, 113.4, 112.3, 15.5; IR (cm^−1^): 3361 (broad), 3051, 2953, 1689, 1571, 1431, 1192, 922, 836, 707; HRMS (*m*/*z*): [M + H]^+^ calculated for (C_13_H_12_ClOS)^+^: 251.0292; found: 251.0305.

### 3h: 2′-bromo-5-(methylthio)-[1,1′-biphenyl]-3-ol

Yield: 57%; 0.25*R*_f_ (10% ethylacetate in hexane), red viscous liquid; ^1^H-NMR (400 MHz, CDCl_3_) *δ* 7.65 (dd, *J* = 8.0, 1.1 Hz, 1H, Ar–H), 7.36–7.29 (m, 2H, Ar–H), 7.22–7.18 (m, 1H, Ar–H), 6.83 (t, *J* = 1.5 Hz, 1H, Ar–H), 6.76 (t, *J* = 2.0 Hz, 1H, Ar–H), 6.64 (dd, *J* = 2.2, 1.4 Hz, 1H, Ar–H), 5.23 (s, 1H, –OH), 2.49 (s, 3H, –SCH_3_); ^13^C-NMR (100 MHz, CDCl_3_) *δ* 155.4, 142.9, 141.7, 139.8, 133.1, 131.0, 129.0, 127.3, 122.3, 119.6, 113.3, 112.2, 15.5; IR (cm^−1^): 3360 (broad), 3055, 2952, 1694, 1578, 1419, 1191, 936, 836, 700; HRMS (*m*/*z*): [M + H]^+^ calculated for (C_13_H_12_BrOS)^+^: 294.9787/296.9766; found: 294.9795/296.9772.

### 3i: 2′,4′-dichloro-5-(methylthio)-[1,1′-biphenyl]-3-ol

Yield: 65%; 0.20*R*_f_ (10% ethylacetate in hexane), brown sticky solid; ^1^H-NMR (400 MHz, CDCl_3_) *δ* 7.47 (d, *J* = 1.9 Hz, 1H, Ar–H), 7.28 (d, *J* = 2.1 Hz, 1H, Ar–H), 7.26 (s, 1H, Ar–H), 6.81 (t, *J* = 1.4 Hz, 1H, Ar–H), 6.76 (t, *J* = 1.9 Hz, 1H, Ar–H), 6.63 (t, *J* = 1.8 Hz, 1H, Ar–H), 5.27 (s, 1H, –OH), 2.48 (s, 3H, –SCH_3_); ^13^C-NMR (100 MHz, CDCl_3_) *δ* 155.6, 140.2, 140.1, 138.2, 133.9, 133.1, 131.8, 129.7, 127.1, 119.5, 113.2, 112.5, 15.5; IR (cm^−1^): 3355 (broad), 3055, 2928, 1693, 1575, 1433, 1195, 926, 826, 704; HRMS (*m*/*z*): [M + H]^+^ calculated for (C_13_H_11_Cl_2_OS)^+^: 284.9902; found: 284.9907.

### 3j: 3-(methylthio)-5-(naphthalen-1-yl)phenol

Yield: 64%; 0.30*R*_f_ (10% ethylacetate in hexane), brown sticky solid; ^1^H-NMR (400 MHz, CDCl_3_) *δ* 7.93–7.85 (m, 3H, Ar–H), 7.52–7.48 (m, 2H, Ar–H), 7.46–7.39 (m, 2H, Ar–H), 6.94 (t, *J* = 1.6 Hz, 1H, Ar–H), 6.82 (t, *J* = 2.1 Hz, 1H, Ar–H), 6.73 (t, *J* = 2.0 Hz, 1H, Ar–H), 5.76 (s, 1H, –OH), 2.49 (s, 3H, –SCH_3_); ^13^C-NMR (100 MHz, CDCl_3_) *δ* 155.7, 142.6, 140.0, 139.3, 133.6, 131.3, 128.2, 127.8, 126.6, 126.1, 125.9, 125.8, 125.2, 120.1, 114.0, 111.8, 15.4; IR (cm^−1^): 3348 (broad), 3059, 2952, 1692, 1572, 1426, 1192, 919, 833, 701; HRMS (*m*/*z*): [M + H]^+^ calculated for (C_17_H_15_OS)^+^: 267.0838; found: 267.0847.

### 3k: 3-(methylthio)-5-(naphthalen-2-yl)phenol

Yield: 70%; 0.30*R*_f_ (10% ethylacetate in hexane), brown sticky solid; ^1^H-NMR (400 MHz, CDCl_3_) *δ* 8.00 (s, 1H, Ar–H), 7.91–7.85 (m, 3H, Ar–H), 7.68 (dd, *J* = 8.5, 1.8 Hz, 1H, Ar–H), 7.52–7.49 (m, 2H, Ar–H), 7.17 (t, *J* = 1.4 Hz, 1H, Ar–H), 6.95–6.94 (m, 1H, Ar–H), 6.77 (t, *J* = 1.9 Hz, 1H, Ar–H), 5.34 (s, 1H, –OH), 2.54 (s, 3H, –SCH_3_); ^13^C-NMR (100 MHz, CDCl_3_) *δ* 156.3, 143.2, 140.6, 137.7, 133.5, 132.8, 128.4, 128.2, 127.6, 126.4, 126.1, 125.9, 125.3, 117.9, 111.9, 111.4, 15.7; IR (cm^−1^): 3362 (broad), 3056, 2949, 1692, 1576, 1426, 1193, 918, 834, 701; HRMS (*m*/*z*): [M + H]^+^ calculated for (C_17_H_15_OS)^+^: 267.0838; found: 267.0848.

### 3l: 3-(furan-2-yl)-5-(methylthio)phenol

Yield: 64%; 0.30*R*_f_ (10% ethylacetate in hexane), red viscous liquid; ^1^H-NMR (400 MHz, CDCl_3_) *δ* 7.28 (d, *J* = 4.3 Hz, 2H, Ar–H), 7.06 (t, *J* = 4.3 Hz, 2H, Ar–H), 6.86 (t, *J* = 1.9 Hz, 1H, Ar–H), 6.67 (t, *J* = 1.9 Hz, 1H, Ar–H), 5.94 (s, 1H, –OH), 2.49 (s, 3H, –SCH_3_); ^13^C-NMR (100 MHz, CDCl_3_) *δ* 156.3, 143.4, 140.7, 136.1, 127.9, 125.1, 123.6, 116.3, 112.1, 109.9, 15.5; IR (cm^−1^): 3357 (broad), 3056, 2951, 1690, 1570, 1421, 1194, 916, 845, 699; HRMS (*m*/*z*): [M + H]^+^ calculated for (C_11_H_11_O_2_S)^+^: 207.0474; found: 207.0481.

### 3m: 3-(methylthio)-5-(thiophen-2-yl)phenol

Yield: 69%; 0.30*R*_f_ (10% ethylacetate in hexane), red viscous liquid; ^1^H-NMR (400 MHz, CDCl_3_) *δ* 7.43 (d, *J* = 1.5 Hz, 1H, Ar–H), 7.12 (t, *J* = 1.4 Hz, 1H, Ar–H), 6.91–6.90 (m, 1H, Ar–H), 6.64 (t, *J* = 1.9 Hz, 1H, Ar–H), 6.61 (d, *J* = 3.3 Hz, 1H, Ar–H), 6.44 (q, *J* = 1.7 Hz, 1H, Ar–H), 5.55 (s, 1H, –OH), 2.48 (s, 3H, –SCH_3_); ^13^C-NMR (100 MHz, CDCl_3_) *δ* 156.2, 153.0, 142.3, 140.6, 132.5, 114.2, 112.0, 111.7, 107.6, 105.8, 15.5; IR (cm^−1^): 3349 (broad), 3059, 2954, 1688, 1572, 1426, 1196, 919, 851, 703; HRMS (*m*/*z*): [M + H]^+^ calculated for (C_11_H_11_OS_2_)^+^: 223.0246; found: 223.0252.

### General protocol for the synthesis of 1 and/or 6-substituted 5-aryl-3-methylthio-phenols (5a–l)

To a nitrogen-flushed vial, α-aroyl ketene dithioacetal (1, 0.3 mmol), ketone (4, 0.5 mmol) and NaH (0.75 mmol) were mixed, and 1.5 mL of DMF was added. The mixture was stirred at 50 °C, and the reaction was observed by TLC in regular time intervals. On completion of the reaction, the crude was neutralized using 10% HCl and then extracted 3–4 times with ethyl acetate and water. The organic layer was collected, dried, and purified by column chromatography using 10% of ethyl acetate in hexane.

### 5a: 2,4-dimethyl-5-(methylthio)-[1,1′-biphenyl]-3-ol

Yield: 78%; 0.30*R*_f_ (10% ethylacetate in hexane), brown viscous liquid; ^1^H-NMR (400 MHz, CDCl_3_) *δ* 7.44–7.41 (m, 2H, Ar–H), 7.36 (t, *J* = 7.3 Hz, 1H, Ar–H), 7.32–7.30 (m, 2H, Ar–H), 6.74 (s, 1H, Ar–H), 4.85 (s, 1H, –OH), 2.43 (s, 3H, –SCH_3_), 2.34 (s, 3H, –CH_3_), 2.13 (s, 3H, –CH_3_); ^13^C-NMR (100 MHz, CDCl_3_) *δ* 152.1, 141.5, 141.0, 135.4, 129.3, 128.1, 126.9, 120.2, 119.3, 117.9, 16.1, 13.1, 12.4; IR (cm^−1^): 3579 (broad), 3036, 2925, 1624, 1506, 1435, 1196, 997, 856, 718; HRMS (*m*/*z*): [M + H]^+^ calculated for (C_15_H_17_OS)^+^: 245.0995; found: 245.0995.

### 5b: 2′-chloro-2,4-dimethyl-5-(methylthio)-[1,1′-biphenyl]-3-ol

Yield: 64%; 0.35*R*_f_ (10% ethylacetate in hexane), brown viscous liquid; ^1^H-NMR (400 MHz, CDCl_3_) *δ* 7.46 (q, *J* = 3.1 Hz, 1H, Ar–H), 7.31 (q, *J* = 3.1 Hz, 2H, Ar–H), 7.24–7.22 (m, 1H, Ar–H), 6.62 (s, 1H, Ar–H), 4.81 (s, 1H, –OH), 2.41 (s, 3H, –SCH_3_), 2.33 (s, 3H, –CH_3_), 1.99 (s, 3H, –CH_3_); ^13^C-NMR (100 MHz, CDCl_3_) *δ* 151.8, 140.1, 138.2, 135.5, 133.6, 131.3, 129.3, 128.7, 126.6, 120.7, 118.8, 118.7, 16.1, 12.8, 12.5; IR (cm^−1^): 3589 (broad), 3048, 2932, 1634, 1513, 1426, 1191, 1003, 857, 709; HRMS (*m*/*z*): [M + H]^+^ calculated for (C_15_H_16_ClOS)^+^: 279.0605; found: 279.0619.

### 5c: 2′-bromo-2,4-dimethyl-5-(methylthio)-[1,1′-biphenyl]-3-ol

Yield: 59%; 0.30*R*_f_ (10% ethylacetate in hexane), brown viscous liquid; ^1^H-NMR (400 MHz, CDCl_3_) *δ* 7.67–7.64 (m, 1H, Ar–H), 7.35 (t, *J* = 7.5 Hz, 1H, Ar–H), 7.25–7.20 (m, 2H, Ar–H), 6.61 (s, 1H, Ar–H), 4.80 (s, 1H, –OH), 2.43 (s, 3H, –SCH_3_), 2.33 (s, 3H, –CH_3_), 1.97 (s, 3H, –CH_3_); ^13^C-NMR (100 MHz, CDCl_3_) *δ* 151.8, 142.2, 139.9, 135.4, 132.5, 131.1, 128.8, 127.2, 123.9, 120.7, 118.7, 118.5, 16.1, 12.8, 12.5; IR (cm^−1^): 3598 (broad), 3046, 2934, 1629, 1507, 1427, 1194, 990, 842, 713; HRMS (*m*/*z*): [M + H]^+^ calculated for (C_15_H_16_BrOS)^+^: 323.0100/325.0079; found: 323.0110/325.0089.

### 5d: 2,6-dimethyl-3-(methylthio)-5-(naphthalen-2-yl)phenol

Yield: 73%; 0.30*R*_f_ (10% ethylacetate in hexane), brown viscous liquid; ^1^H-NMR (400 MHz, CDCl_3_) *δ* 7.91–7.86 (m, 3H, Ar–H), 7.77 (s, 1H, Ar–H), 7.52 (t, *J* = 3.7 Hz, 2H, Ar–H), 7.45 (d, *J* = 8.4 Hz, 1H, Ar–H), 6.83 (s, 1H, Ar–H), 4.90 (s, 1H, –OH), 2.45 (s, 3H, –SCH_3_), 2.37 (s, 3H, –CH_3_), 2.17 (s, 3H, –CH_3_); ^13^C-NMR (100 MHz, CDCl_3_) *δ* 152.2, 140.9, 139.1, 135.5, 133.2, 132.3, 128.0, 127.8, 127.7, 127.5, 126.2, 125.9, 120.4, 119.6, 118.1, 16.2, 13.2, 12.5; IR (cm^−1^): 3603 (broad), 3036, 2941, 1623, 1504, 1421, 1196, 999, 848, 728; HRMS (*m*/*z*): [M + H]^+^ calculated for (C_19_H_19_OS)^+^: 295.1151; found: 295.1167.

### 5e: 2,6-dimethyl-3-(methylthio)-5-(thiophen-2-yl)phenol

Yield: 69%; 0.30*R*_f_ (10% ethylacetate in hexane), brown viscous liquid; ^1^H-NMR (400 MHz, CDCl_3_) *δ* 7.34 (d, *J* = 5.0 Hz, 1H, Ar–H), 7.09 (dd, *J* = 5.0, 3.7 Hz, 1H, Ar–H), 7.00 (d, *J* = 3.7 Hz, 1H, Ar–H), 6.86 (s, 1H, Ar–H), 4.87 (s, 1H, –OH), 2.44 (s, 3H, –SCH_3_), 2.32 (s, 3H, –CH_3_), 2.26 (s, 3H, –CH_3_); ^13^C-NMR (100 MHz, CDCl_3_) *δ* 152.2, 142.7, 135.6, 133.1, 127.0, 126.7, 125.1, 121.0, 120.1, 118.8, 16.1, 13.2, 12.5; IR (cm^−1^): 3609 (broad), 3040, 2924, 1622, 1498, 1421, 1189, 1007, 840, 725; HRMS (*m*/*z*): [M + H]^+^ calculated for (C_13_H_15_OS_2_)^+^: 251.0559; found: 251.0562.

### 5fa and 5fb: 4-methyl-5-(methylthio)-[1,1′-biphenyl]-3-ol and 2-methyl-5-(methylthio)-[1,1′-biphenyl]-3-ol

Yield: 74% (5fa = 52% and 5fb = 22%); 0.30*R*_f_ (10% ethylacetate in hexane), brown viscous liquid; ^1^H-NMR (400 MHz, CDCl_3_) *δ* 7.56–7.54 (m, 2H, Ar–H), 7.45–7.40 (m, 3H, Ar–H), 7.37–7.35 (m, 1H, Ar–H), 7.34–7.29 (m, 1H, Ar–H), 7.01 (d, *J* = 1.5 Hz, 1H, Ar–H), 6.83 (d, *J* = 1.4 Hz, 1H, Ar–H), 6.78 (d, *J* = 1.9 Hz, 0H, Ar–H), 6.75 (d, *J* = 1.8 Hz, 0H, Ar–H), 5.17 (s, 1H, –OH), 2.52 (s, 3H, –SCH_3_), 2.46 (s, 1H, –SCH_3_), 2.31 (s, 3H, –CH_3_), 2.11 (s, 1H, –CH_3_); ^13^C-NMR (100 MHz, CDCl_3_) *δ* 154.3, 153.7, 141.2, 140.7, 140.1, 139.7, 135.9, 129.1, 128.7, 128.5, 128.1, 127.8, 127.4, 127.0, 127.0, 120.7, 120.5, 116.4, 112.1, 110.9, 17.4, 15.9, 12.7, 11.9; IR (cm^−1^): 3570 (broad), 3041, 2929, 2863, 1632, 1506, 1433, 1193, 998, 851, 730; HRMS (*m*/*z*): [M + H]^+^ calculated for (C_14_H_15_OS)^+^: 231.0838; found: 231.0844.

### Reaction performed in *t*-BuOK

Yield 67% (5fa = 55% and 5fb = 12%) ^1^H-NMR (400 MHz, CDCl_3_) *δ* 7.55 (dd, *J* = 8.2, 1.4 Hz, 2H, Ar–H), 7.45–7.40 (m, 2H, Ar–H), 7.37–7.35 (m, 1H, Ar–H), 7.33–7.29 (m, 1H, Ar–H), 6.99 (d, *J* = 1.8 Hz, 1H, Ar–H), 6.83 (d, *J* = 1.4 Hz, 1H, Ar–H), 6.76 (d, *J* = 1.8 Hz, 0H, Ar–H), 6.74 (d, *J* = 1.8 Hz, 0H, Ar–H), 5.04 (2s, 1H, –OH), 2.51 (s, 3H, –SCH_3_), 2.46 (s, 1H, –SCH_3_), 2.30 (s, 3H, –CH_3_), 2.10 (s, 1H, –CH_3_).

### 5g: 4-methoxy-6′-(methylthio)-[1,1′:4′,1′′-terphenyl]-2′-ol

Yield: 81%; 0.30*R*_f_ (10% ethylacetate in hexane), yellowish sticky solid; ^1^H-NMR (400 MHz, CDCl_3_) *δ* 7.62 (d, *J* = 7.7 Hz, 2H, Ar–H), 7.48–7.45 (m, 2H, Ar–H), 7.40–7.36 (m, 1H, Ar–H), 7.32 (d, *J* = 8.7 Hz, 2H, Ar–H), 7.08 (d, *J* = 6.7 Hz, 2H, Ar–H), 7.04 (d, *J* = 1.6 Hz, 1H, Ar–H), 7.01 (d, *J* = 1.5 Hz, 1H, Ar–H), 4.95 (s, 1H, –OH), 3.88 (s, 3H, –OCH_3_), 2.41 (s, 3H, –SCH_3_); ^13^C-NMR (100 MHz, CDCl_3_) *δ* 159.9, 153.2, 142.4, 140.7, 139.7, 131.9, 128.8, 127.6, 127.1, 125.1, 124.5, 115.1, 115.0, 110.6, 55.3, 15.8; IR (cm^−1^): 3605 (broad), 3038, 2930, 2857, 1619, 1496, 1438, 1220, 1194, 1000, 848, 724; HRMS (*m*/*z*): [M + H]^+^ calculated for (C_20_H_19_O_2_S)^+^: 323.1100; found: 323.1108.

### 5h: 4,4′′-dimethoxy-6′-(methylthio)-[1,1′:4′,1′′-terphenyl]-2′-ol

Yield: 84%; 0.35*R*_f_ (10% ethylacetate in hexane), yellowish sticky solid; ^1^H-NMR (400 MHz, CDCl_3_) *δ* 7.56 (dd, *J* = 6.6, 2.1 Hz, 2H, Ar–H), 7.31 (dd, *J* = 6.4, 2.3 Hz, 2H, Ar–H), 7.07 (dd, *J* = 6.6, 2.1 Hz, 2H, Ar–H), 7.01–6.98 (m, 3H, Ar–H), 6.96 (d, *J* = 1.4 Hz, 1H, Ar–H), 4.92 (s, 1H, –OH), 3.87 (2s, 6H, –OCH_3_), 2.40 (s, 3H, –SCH_3_); ^13^C-NMR (100 MHz, CDCl_3_) *δ* 159.9, 159.4, 153.2, 142.0, 139.6, 133.2, 131.9, 128.1, 125.3, 124.0, 115.0, 114.8, 114.2, 110.2, 55.3, 55.3, 15.8; IR (cm^−1^): 3597 (broad), 3029, 2936, 2855, 1627, 1499, 1434, 1226, 1191, 990, 848, 730; HRMS (*m*/*z*): [M + H]^+^ calculated for (C_21_H_21_O_3_S)^+^: 353.1206; found: 353.1210.

### 5i: 4′-methoxy-6-(methylthio)-4-(naphthalen-2-yl)-[1,1′-biphenyl]-2-ol

Yield: 77%; 0.35*R*_f_ (10% ethylacetate in hexane), yellowish sticky solid; ^1^H-NMR (400 MHz, CDCl_3_) *δ* 8.09 (s, 1H, Ar–H), 7.96–7.88 (m, 3H, Ar–H), 7.78 (dd, *J* = 8.5, 1.8 Hz, 1H, Ar–H), 7.56–7.49 (m, 2H, Ar–H), 7.36 (d, *J* = 8.7 Hz, 2H, Ar–H), 7.17 (dd, *J* = 13.8, 1.4 Hz, 2H, Ar–H), 7.10 (d, *J* = 8.8 Hz, 2H, Ar–H), 5.06 (s, 1H, –OH), 3.89 (s, 3H, –OCH_3_), 2.46 (s, 3H, –SCH_3_); ^13^C-NMR (100 MHz, CDCl_3_) *δ* 159.9, 153.3, 142.3, 139.9, 138.0, 133.5, 132.8, 131.9, 128.4, 128.2, 127.6, 126.4, 126.1, 125.8, 125.4, 125.2, 124.7, 115.4, 115.0, 110.9, 55.3, 15.8; IR (cm^−1^): 3593 (broad), 3034, 2930, 2858, 1621, 1501, 1435, 1228, 1195, 996, 850, 721; HRMS (*m*/*z*): [M + H]^+^ calculated for (C_24_H_21_O_2_S)^+^: 373.1257; found: 373.1271.

### 5j: 4-(furan-2-yl)-4′-methoxy-6-(methylthio)-[1,1′-biphenyl]-2-ol

Yield: 72%; 0.35*R*_f_ (10% ethylacetate in hexane), yellowish sticky solid; ^1^H-NMR (400 MHz, CDCl_3_) *δ* 7.49 (d, *J* = 1.4 Hz, 1H, Ar–H), 7.29 (d, *J* = 8.7 Hz, 2H, Ar–H), 7.13 (dd, *J* = 10.5, 1.4 Hz, 2H, Ar–H), 7.06 (d, *J* = 8.8 Hz, 2H, Ar–H), 6.69 (d, *J* = 3.4 Hz, 1H, Ar–H), 6.50 (q, *J* = 1.7 Hz, 1H, Ar–H), 4.99 (s, 1H, –OH), 3.87 (s, 3H, –OCH_3_), 2.42 (s, 3H, –SCH_3_); ^13^C-NMR (100 MHz, CDCl_3_) *δ* 159.9, 153.3, 153.2, 142.2, 139.9, 131.8, 131.6, 125.2, 124.6, 114.9, 111.7, 107.2, 105.6, 55.2, 15.6; IR (cm^−1^): 3585 (broad), 3026, 2939, 2857, 1628, 1503, 1440, 1221, 1194, 998, 853, 719; HRMS (*m*/*z*): [M + H]^+^ calculated for (C_18_H_17_O_3_S)^+^: 313.0893; found: 313.0905.

### 5k: 5′-(methylthio)-4′-phenyl-[1,1′:2′,1′′-terphenyl]-3′-ol

Yield: 79%; 0.30*R*_f_ (10% ethylacetate in hexane), yellowish solid; ^1^H-NMR (400 MHz, CDCl_3_) *δ* 7.55–7.51 (m, 2H, ArH), 7.47–7.43 (m, 3H, ArH), 7.29–7.27 (m, 1H, ArH), 7.25–7.21 (m, 2H, ArH), 7.20–7.14 (m, 7H, ArH), 6.90 (s, 1H, –ArH), 5.05 (s, 1H, –OH), 2.38 (s, 3H, –SCH_3_); ^13^C-NMR (100 MHz, CDCl_3_) *δ* 150.0, 142.0, 140.9, 138.3, 135.5, 134.7, 131.1, 130.5, 129.7, 129.0, 128.5, 128.4, 127.8, 127.2, 126.6, 125.4, 123.9, 117.9, 15.7; IR (cm^−1^): 3580 (broad), 3024, 2941, 2855, 1634, 1509, 1441, 1219, 1196, 998, 853, 720; HRMS (*m*/*z*): [M + H]^+^ calculated for (C_25_H_21_OS)^+^: 369.1308; found: 369.1310.

### 5l: 4′-(furan-2-yl)-6′-(methylthio)-[1,1′:3′,1′′-terphenyl]-2′-ol

Yield: 73%; 0.30*R*_f_ (10% ethylacetate in hexane), yellowish solid; ^1^H-NMR (400 MHz, CDCl_3_) *δ* 7.54–7.48 (m, 4H, –ArH), 7.47–7.41 (m, 5H, –ArH), 7.38–7.34 (m, 3H, –ArH), 6.22 (d, *J* = 3.4 Hz, 1H, –ArH), 5.34 (s, 1H, –ArH), 4.88 (s, 1H, –OH), 2.47 (s, 3H, –SCH_3_); ^13^C-NMR (100 MHz, CDCl_3_) *δ* 152.0, 150.2, 141.4, 138.8, 135.8, 134.6, 130.4 (2C), 130.2, 129.3, 128.9, 128.4, 128.2, 125.3, 122.0, 114.0, 111.4, 109.6, 15.7; IR (cm^−1^): 3585 (broad), 3028, 2936, 2857, 1630, 1505, 1440, 1222, 1194, 997, 861, 719; HRMS (*m*/*z*): [M + H]^+^ calculated for (C_23_H_19_O_2_S)^+^: 359.1100; found: 359.1105.

### General protocol for the synthesis of 1-(methylthio)-9,10-dihydrophenanthren-3-ols (6a–f)

To a nitrogen flushed vacuum dried vial, 2-(bis(methylthio)methylene)-6-(methoxy/*H*)-3,4-dihydronaphthalen-1(2*H*)-one (1, 0.3 mmol), ketone (4, 0.5 mmol) and NaH (0.75 mmol) were mixed, and 1.5 mL of DMF was added. The mixture was stirred at 50 °C, and the reaction was observed using TLC in regular time intervals. On completion of the reaction, the crude was neutralized by 10% HCl and then extracted 3–4 times with ethyl acetate and water. The organic layer was collected, dried, and purified by column chromatography using 10% of ethyl acetate in hexane.

### 6a: 1-(methylthio)-9,10-dihydrophenanthren-3-ol

Yield: 81%; 0.40*R*_f_ (10% ethylacetate in hexane), cream coloured sticky solid; ^1^H-NMR (400 MHz, CDCl_3_) *δ* 7.63 (d, *J* = 7.3 Hz, 1H, Ar–H), 7.30–7.27 (m, 1H, Ar–H), 7.24 (d, *J* = 3.8 Hz, 2H, Ar–H), 7.06 (d, *J* = 2.3 Hz, 1H, Ar–H), 6.69 (d, *J* = 2.5 Hz, 1H, Ar–H), 5.20 (s, 1H, –OH), 2.85 (s, 4H, –CH_2_), 2.46 (s, 3H, –SCH_3_); ^13^C-NMR (100 MHz, CDCl_3_) *δ* 154.6, 138.2, 137.4, 136.0, 134.0, 127.9, 127.7, 127.4, 126.8, 124.1, 111.4, 107.7, 28.8, 24.3, 15.7; IR (cm^−1^): 3654, 3036, 2935, 2851, 1626, 1469, 1218, 1072, 853, 768, 720; HRMS (*m*/*z*): [M + H]^+^ calculated for (C_15_H_15_OS)^+^: 243.0838; found: 243.0849.

### 6b: 2,4-dimethyl-1-(methylthio)-9,10-dihydrophenanthren-3-ol

Yield: 86%; 0.40*R*_f_ (10% ethylacetate in hexane), cream coloured sticky solid; ^1^H-NMR (400 MHz, CDCl_3_) *δ* 7.49 (d, *J* = 7.1 Hz, 1H, Ar–H), 7.30 (d, *J* = 7.1 Hz, 1H, Ar–H), 7.27–7.25 (m, 1H, Ar–H), 7.24–7.22 (m, 1H, Ar–H), 4.80 (s, 1H, –OH), 3.13–3.10 (t, *J* = 6.5 Hz, 2H, –CH_2_), 2.72 (t, *J* = 6.6 Hz, 2H, –CH_2_), 2.59 (s, 3H, –SCH_3_), 2.50 (s, 3H, –CH_3_), 2.21 (s, 3H, –CH_3_); ^13^C-NMR (100 MHz, CDCl_3_) *δ* 151.6, 140.1, 136.2, 134.8, 134.5, 132.5, 128.6, 127.1, 126.7, 125.7, 125.5, 120.9, 30.2, 28.5, 19.1, 15.6, 14.6; IR (cm^−1^): 3660, 3036, 2930, 2846, 1622, 1470, 1227, 1065, 861, 764, 718; HRMS (*m*/*z*): [M + H]^+^ calculated for (C_17_H_19_OS)^+^: 271.1151; found: 271.1157.

### 6c: 2-(4-methoxyphenyl)-1-(methylthio)-9,10-dihydrophenanthren-3-ol

Yield: 88%; 0.35*R*_f_ (10% ethylacetate in hexane), cream coloured sticky solid; ^1^H-NMR (400 MHz, CDCl_3_) *δ* 7.72 (d, *J* = 7.6 Hz, 1H, Ar–H), 7.43 (s, 1H, Ar–H), 7.35–7.27 (m, 5H, Ar–H), 7.07 (d, *J* = 8.7 Hz, 2H, Ar–H), 4.93 (s, 1H, –OH), 3.90 (s, 3H, –OCH_3_), 3.21 (t, *J* = 7.1 Hz, 2H, –CH_2_), 2.91–2.88 (t, *J* = 7.2 Hz, 2H, –CH_2_), 2.03 (s, 3H, –SCH_3_); ^13^C-NMR (100 MHz, CDCl_3_) *δ* 159.4, 152.0, 137.7, 136.0, 135.1, 134.3, 133.4, 131.6, 131.4, 127.8, 127.7, 127.5, 126.9, 124.1, 114.5, 111.2, 55.2, 29.2, 26.4, 19.3; IR (cm^−1^): 3621, 3044, 2931, 2848, 1617, 1478, 1215, 1184, 1072, 864, 775, 716; HRMS (*m*/*z*): [M + H]^+^ calculated for (C_22_H_21_O_2_S)^+^: 349.1257; found: 349.1274.

### 6d: 1-(methylthio)-2,4-diphenyl-9,10-dihydrophenanthren-3-ol

Yield: 86%; 0.30*R*_f_ (10% ethylacetate in hexane), cream coloured solid; ^1^H-NMR (400 MHz, CDCl_3_) *δ* 7.54–7.50 (m, 2H, Ar–H), 7.47–7.38 (m, 5H, Ar–H), 7.36–7.32 (m, 3H, Ar–H), 7.24 (d, *J* = 7.4 Hz, 1H, Ar–H), 7.07 (td, *J* = 7.3, 1.4 Hz, 1H, Ar–H), 6.69–6.84 (m, 2H, Ar–H), 5.01 (s, 1H, –OH), 3.21 (t, *J* = 6.7 Hz, 2H, –CH_3_), 2.88–2.85 (m, 2H, –CH_3_), 2.09 (s, 3H, –SCH_3_); ^13^C-NMR (100 MHz, CDCl_3_) *δ* 149.0, 140.0, 137.1, 136.8, 135.9, 134.9, 134.3, 134.0, 131.8, 130.9, 130.3, 129.6, 129.0, 128.5, 127.9, 127.6, 127.0, 126.6, 126.2, 125.2, 30.1, 28.1, 19.3; IR (cm^−1^): 3580 (broad), 3024, 2941, 2855, 1634, 1509, 1441, 1219, 1196, 998, 853, 720; HRMS (*m*/*z*): [M + H]^+^ calculated for (C_27_H_23_OS)^+^: 395.1464; found: 395.1465.

### 6e: 7-methoxy-1-(methylthio)-9,10-dihydrophenanthren-3-ol

Yield: 85%; 0.35*R*_f_ (10% ethylacetate in hexane), cream coloured sticky solid; ^1^H-NMR (400 MHz, CDCl_3_) *δ* 7.56 (d, *J* = 8.5 Hz, 1H, Ar–H), 6.98 (d, *J* = 2.2 Hz, 1H, Ar–H), 6.83–6.77 (m, 2H, Ar–H), 6.63 (d, *J* = 2.2 Hz, 1H, Ar–H), 5.15 (s, 1H, –OH), 3.84 (s, 3H, –OCH_3_), 2.83 (broad d, *J* = 1.2, 4H, –CH_2_), 2.45 (s, 3H, –SCH_3_); ^13^C-NMR (100 MHz, CDCl_3_) *δ* 159.2, 154.6, 139.1, 138.1, 136.0, 127.0, 126.6, 125.4, 113.2, 112.3, 110.7, 107.2, 55.3, 29.2, 24.4, 15.7; IR (cm^−1^): 3666, 3032, 2931, 2847, 1621, 1476, 1223, 1066, 863, 765, 713; HRMS (*m*/*z*): [M + H]^+^ calculated for (C_16_H_17_O_2_S)^+^: 273.0944; found: 273.0953.

### 6f: 7-methoxy-2-(4-methoxyphenyl)-1-(methylthio)-9,10-dihydrophenanthren-3-ol

Yield: 93%; 0.30*R*_f_ (10% ethylacetate in hexane), cream coloured solid; ^1^H-NMR (400 MHz, CDCl_3_) *δ* 7.63 (d, *J* = 8.5 Hz, 1H, Ar–H), 7.33 (s, 1H, Ar–H), 7.28 (dd, *J* = 6.6, 2.1 Hz, 2H, Ar–H), 7.05 (dd, *J* = 6.7, 2.0 Hz, 2H, Ar–H), 6.85 (dd, *J* = 8.5, 2.6 Hz, 1H, Ar–H), 6.80 (d, *J* = 2.6 Hz, 1H, Ar–H), 4.84 (s, 1H, –OH), 3.87 (s, 3H, –OCH_3_), 3.84 (s, 3H, –OCH_3_), 3.18 (t, *J* = 7.2 Hz, 2H, –CH_2_), 2.85 (t, *J* = 7.2 Hz, 2H, –CH_2_), 2.00 (s, 3H, –SCH_3_); ^13^C-NMR (100 MHz, CDCl_3_) *δ* 159.4, 159.3, 152.0, 139.4, 136.0, 135.0, 132.6, 131.7, 130.6, 127.6, 127.2, 125.4, 114.5, 113.1, 112.3, 110.5, 55.3, 55.3, 29.7, 26.5, 19.3; IR (cm^−1^): 3623, 3046, 2933, 2846, 1614, 1474, 1217, 1188, 1075, 867, 772, 711; HRMS (*m*/*z*): [M + H]^+^ calculated for (C_23_H_23_O_3_S)^+^: 379.1362; found: 379.1375.

### 7: 3-cyclopropyl-5-(methylthio)phenol

Yield: 61%; 0.40*R*_f_ (10% ethylacetate in hexane), yellowish liquid; ^1^H-NMR (400 MHz, CDCl_3_) *δ* 6.57 (s, 1H, Ar–H), 6.52 (t, *J* = 1.9 Hz, 1H, Ar–H), 6.29 (t, *J* = 1.9 Hz, 1H, Ar–H), 5.35 (s, 1H, –OH), 2.44 (s, 3H, –SCH_3_), 1.80 (m, 1H, –CH), 0.93 (m, 2H, –CH_2_), 0.66 (m, 2H, –CH_2_); ^13^C-NMR (100 MHz, CDCl_3_) *δ* 156.0, 146.5, 139.6, 116.5, 110.3, 109.5, 15.6, 15.3, 9.1; HRMS (*m*/*z*): [M + H]^+^ calculated for (C_10_H_13_OS)^+^: 181.0682; found: 181.0690.

## Conclusions

In conclusion, we observed that the synthesis of *m*-aryl phenols is more challenging than that of other aryl phenols. We developed a simple and efficient [3 + 3] cycloaromatization reaction for the synthesis of 3-aryl-5-methylthio-phenols by the reaction of α-aroyl-ketene dithioacetal and ketones, containing acidic protons on both sides of the carbonyl groups. Several 3-aryl-phenols, hydroxy-xylenes, 2,5-diaryl-phenols, and 9,10-dihydrophenanthren-3-ols have been synthesized using this strategy. The reaction efficiently works with ketones with alkyl or aryl substitutions but fails with the 1,3-dicarbonyl system. A plausible reaction pathway was proposed for this reaction. We will further develop new entities from these synthesized phenols and ketene dithioacetals.

## Conflicts of interest

There are no conflicts to declare.

## Supplementary Material

RA-013-D3RA05421G-s001

RA-013-D3RA05421G-s002

## References

[cit1] Teponno R. B., Kusari S., Spiteller M. (2016). Recent advances in research on lignans and neolignans. Nat. Prod. Rep..

[cit2] (a) Tyman J. H. P. , Synthetic and Natural Phenols, Elsevier, New York, 1996

[cit3] Heitman L. H., Narlawar R., de Vries H., Willemsen M. N., Wolfram D., Brussee J., IJzerman A. P. (2009). Substituted terphenyl compounds as the first class of low molecular weight allosteric inhibitors of the luteinizing hormone receptor. J. Med. Chem..

[cit4] (a) Liu W. , US Pat., 7300639B2, 2007

[cit5] Stanton J. L., Cahill E., Dotson R., Tan J., Tomaselli H. C., Wasvary J. M., Stephan Z. F., Steele R. E. (2000). Synthesis and biological activity of phenoxyphenyl oxamic acid derivatives related to L-thyronine. Bioorg. Med. Chem. Lett..

[cit6] (b) Nussbaum K. and Hoffmann M., US Pat., 6753451B2, 2004

[cit7] Eichinger K., Nussbaumer P., Balkan S., Schulz G. (1987). Neue Synthesen alkylaryl-und diaryl-disubstituierter Phenole und Salicylsäure-ethylester. Synthesis.

[cit8] Katritzky A. R., Belyakov S. A., Henderson S. A., Steel P. J. (1997). Improved Syntheses of 3, 5-Diaryl-Substituted Phenols. J. Org. Chem..

[cit9] Qian J., Yi W., Huang X., Miao Y., Zhang J., Cai C., Zhang W. (2015). One-pot synthesis of 3, 5-disubstituted and polysubstituted phenols from acyclic precursors. Org. Lett..

[cit10] Huffman M. A., Liebeskind L. S. (1990). Insertion of (.eta. 5-indeny) cobalt (I) into cyclobutenones: the first synthesis of phenols from isolated vinylketene complexes. J. Am. Chem. Soc..

[cit11] Barun O., Nandi S., Panda K., Ila H., Junjappa H. (2002). [4+ 2] cycloaromatization of 4-bis(methylthio)-3-buten-2-one with active methylene ketones: a simple and facile phenol annulation. J. Org. Chem..

[cit12] Xu C., Wang M., Liu Q. (2019). Recent Advances in Metal-Catalyzed Bond-Forming Reactions of Ketene S, S-Acetals. Adv. Synth. Catal..

[cit13] Sham H. L., Betebenner D. A., Chen X., Saldivar A., Vasavanonda S., Kempf D. J., Plattner J. J., Norbeck D. W. (2002). Synthesis and structure–activity relationships of a novel series of HIV-1 protease inhibitors encompassing ABT-378 (Lopinavir). Bioorg. Med. Chem. Lett..

[cit14] Pospíšilová M., Svobodová D., Gasparič J., Macháček M. (1990). Investigation of the colour reaction of phenols with MBTH, II: properties of the isolated products of the reaction with phenol, 2, 6-dimethylphenol and 4-methylphenol. Microchim. Acta.

[cit15] Xiao B., Gong T. J., Liu Z. J., Liu J. H., Luo D. F., Xu J., Liu L. (2011). Synthesis of dibenzofurans via palladium-catalyzed phenol-directed C–H activation/C–O cyclization. J. Am. Chem. Soc..

[cit16] Buchanan A. C., Dunstan T. D. J., Douglas E. C., Poutsma M. L. (1986). Thermolysis of model compounds for coal. Part 5. Enhancement of free-radical chain rearrangement, cyclization, and hydrogenolysis during thermolysis of surface-immobilized bibenzyl. Implications for coal chemistry. J. Am. Chem. Soc..

[cit17] El-Feraly F. S., Cheatham S. F., McChesney J. D. (1985). Synthesis and ^13^C nuclear magnetic resonance assignments of cannithrene 1: a cannabis constituent. Can. J. Chem..

[cit18] Paredes E., Brasca R., Kneeteman M., Mancini P. M. (2007). A novel application of the Diels–Alder reaction: nitronaphthalenes as normal electron demand dienophiles. Tetrahedron.

