# Supplementary material for: 1,3-Dianionic annulation of ketones with ketene dithioacetal: a modified route to 3-aryl/cyclopropyl-5-thiomethyl-phenols and 1-(methylthio)-9,10-dihydrophenanthren-3-ols
Source: RSC Adv. 2023 Nov 22;13(48):34299–307. doi: 10.1039/d3ra05421g (PMC10664189; doi:10.1039/d3ra05421g)
Supplement: RA-013-D3RA05421G-s001 [file RA-013-D3RA05421G-s001.pdf]

Electronic Supplementary Material (ESI) for RSC Advances.
This journal is © The Royal Society of Chemistry 2023

1,3-Dianionic annulation of ketones with ketene dithioacetal: A modified route to 3-aryl/cyclopropyl-5-thiomethyl-phenols and 1-(methylthio)-9,10-dihydrophenanthren-3-ols

Ranjay Shaw,[a] Prasoon Prakash,[b] Ismail Althagafi,[c] Nand Gopal Giri,[d] Ramendra Pratap[d] *

[a] Department of Chemistry, GLA University, Mathura, U.P., India-281406
[b] Department of Kinesiology and Educational Psychology, Washington State University, Pullman, WA, USA-99164
[c]Department of Chemistry. Faculty of Science, Umm Al-Qura University, Makkah, Saudi Arabia
[d]Department of Chemistry, Shivaji College, University of Delhi, Raja Garden, New Delhi-110027, India
[e]Department of Chemistry, University of Delhi, North Campus, Delhi, India-110007

Entry	Content	Page No.
1.	General method for synthesis of precursors	S2-S3
2.	General information on experimental	S3
3.	Crystal data of product **5h**	S4-S6
4.	References	S6
5.	^{1}H and ^{13}C NMR spectra of products	S7-S39

1. **General method for synthesis of precursors:** [3,3-bis(methylthio)-1-arylprop-2-en-1-ones] (1)[1]

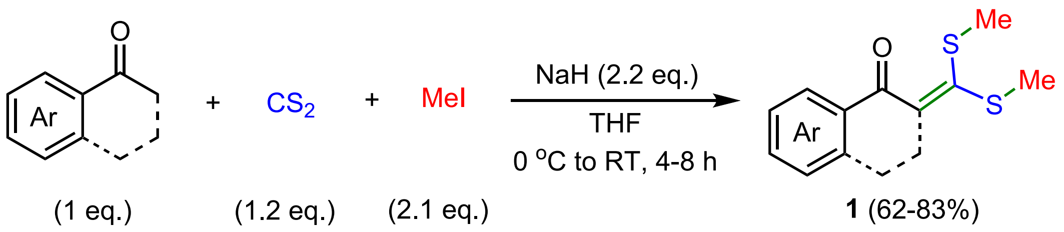

1 (62-83%)

To a stirred solution of NaH (44 mmol) in THF (50 mL) at 0°C, alkyl aryl ketone (20 mmol) was added dropwise manner (in 8-10 minutes) through a dropping funnel. The suspension was stirred for 20 minutes, and carbon disulfide (CS_2) (24 mmol) was added in dropwise over 8-10 minutes. The reaction mixture (RM) was allowed to stir for 30 min, then MeI (42 mmol) was added dropwise over 12-15 minutes. The RM was slowly brought up to room temperature (RT) and stirring continued for another 4-8 hours with monitoring by TLC. After completion of the reaction, RM was poured into crushed ice. Most of the 3,3-bis(methylthio)-1-arylprop-2-en-1-ones were precipitated in crushed ice. The precipitate was filtered and washed with ice-cold water and dried overnight inside a vacuum hood. Few 3,3-bis(methylthio)-1-arylprop-2-en-1-ones were not precipitated on crushed ice, which was extracted with ethyl acetate in ice-cooled water, dried with Na_2SO_4 and concentrated under reduced pressure. The crude mixture was recrystallized with ethyl acetate and n-hexane.

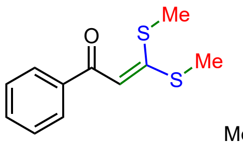

1a

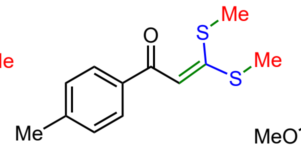

1b

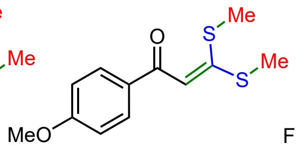

1c

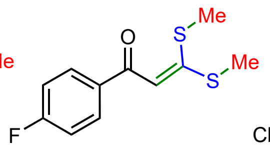

1d

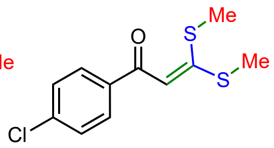

1e

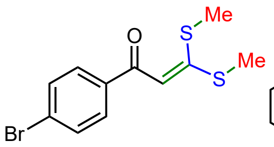

1f

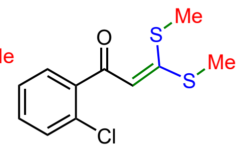

1g

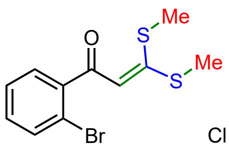

1h

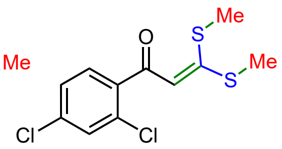

1i

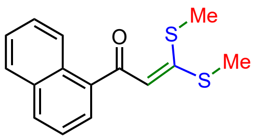

1j

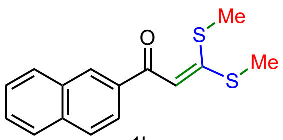

1k

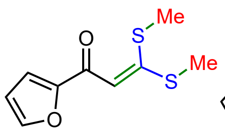

1l

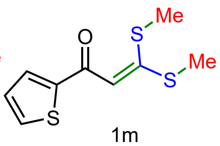

1m

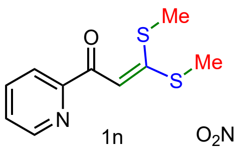

1n

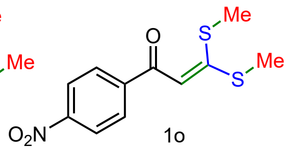

1o

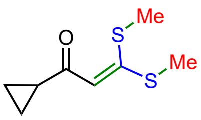

1p

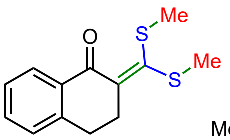

1q

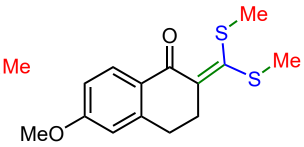

1r

2. General information on experimental:

Commercially available reagents and solvents from Alfa Aesar, Spectrochem, Sigma Aldrich, Fischer Scientific and TCI Chemicals were used without further purification. ^{1}H and ^{13}C NMR spectra were recorded on a 400 MHz and 100 MHz NMR spectrometer (Jeol and Bruker instrument) respectively, and $CDCl_3$ (from Eurisotop) was used as solvent. Chemical shifts for all the compounds are reported in parts per million (ppm) shifts (δ-value). One singlet at δ 7.26 ppm of ^{1}H and a triplet at 77.00 ppm of ^{13}C NMR for $CDCl_3$ were taken as an internal standard. Signal patterns are mentioned as s, singlet; d, doublet; dd, double doublet; t, triplet; q, quartet; m, multiplet; bs, broad singlet and bm, broad multiplet. The coupling constant (J) for protons are given in hertz (Hz). Infrared (IR) spectra were recorded on a Perkine Elmer AX-1 spectrophotometer and reported in wave number (cm^{-1}). HRMS is reported for the peak of $(M+H)^+$ using Agilent G6530AA (LC-HRMS-Q-TOF) spectrometer. Reagent-grade solvents were used for extraction and chromatography. The yield of the product was reported as chromatographically isolated pure materials.

3. Crystal data of product 5h:

Crystal Preparation: The product is a sticky solid at room temperature. To grow the crystal, a highly concentrated (saturated) solution of the compound **5h** was prepared by dissolving it in hot methanol. After that, the compound was cooled to room temperature and kept in the fridge for 3 days. After 3 days of cooling small crystals were generated (with minimum exposure of room temperature).

X-ray structure analysis: Intensity data for the compound **5h** was collected at 288 K on an XtaLAB Synergy, Single source at home/near, HyPix diffractometer using graphite monochromated Mo-Kα radiation λ = 0.71073 Å. The structure was solved by Using Olex2[2] with SHELXT[3] structure solution program using Intrinsic Phasing and refined with the olex2 refine[4] refinement package using Gauss-Newton minimisation. The hydrogen atoms were placed at the calculated positions and included in the last cycles of the refinement. The graphics for publication were prepared by using Mercury software. Crystallographic data collection and structure solution parameters are summarized in Table 1.

Table S1: Crystallographic data of **5h**

Empirical formula	***$C_{21}H_{20}O_3S$***
CCDC	***2297558***
Formula weight	*352.44*
Temperature/K	*288*
Crystal system	*monoclinic*
Space group	*C2/c*
a/Å	*22.2427(13)*
b/Å	*5.4882(4)*
c/Å	*29.653(2)*
α/°	*90*
β/°	*90.096(6)*
γ/°	*90*
Volume/Å^3	*3619.8(4)*
Z	*68*
ρ_{calc}g/cm^3	*1.293*
μ/mm^{-1}	*0.195*
F(000)	*1489.9*
Crystal size/mm^3	*0.1 × 0.1 × 0.1*
Radiation	*Mo Kα (λ = 0.71073)*

2Θ range for data collection/°	*6.6 to 73.96*
Index ranges	*-27 ≤ h ≤ 28, -6 ≤ k ≤ 7, -42 ≤ l ≤ 46*
Reflections collected	*8803*
Independent reflections	*4088 [R_{int} = 0.0816, R_{sigma} = 0.0895]*
Data/restraints/parameters	*4088/0/230*
Goodness-of-fit on F^2	*0.972*
`Final R indexes [I>=2σ (I)]	*R_1 = 0.0509, wR_2 = 0.1128*
Final R indexes [all data]	*R_1 = 0.1103, wR_2 = 0.1462*
Largest diff. peak/hole / e Å^{-3}	*0.39/-0.54*

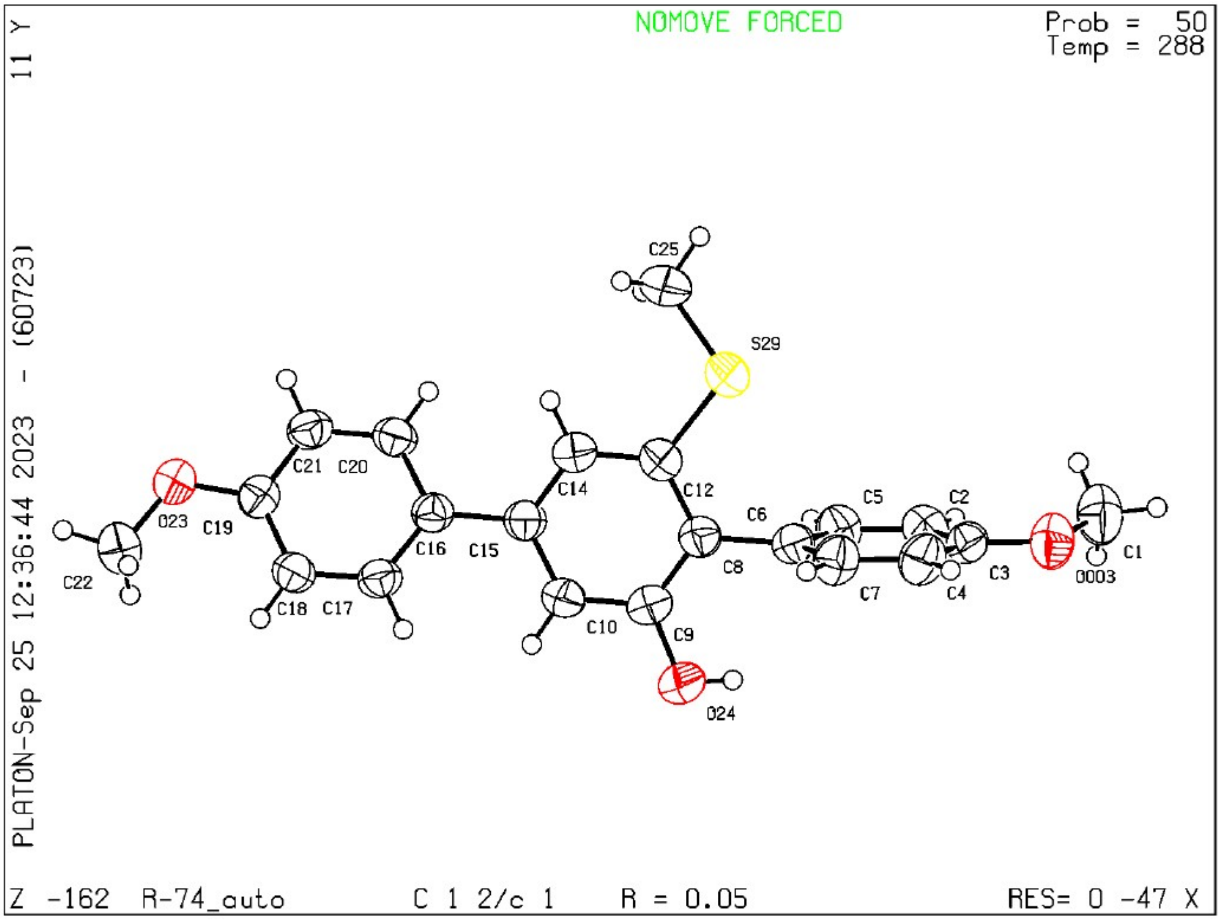

Figure S1. ORTEP diagram of product **5h** in 50% probability.

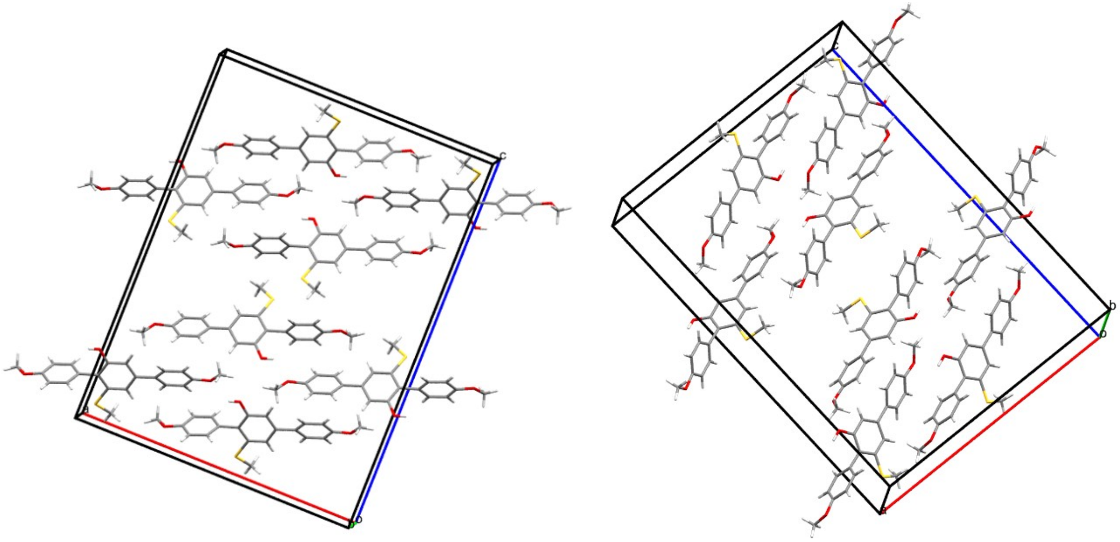

Figure 2. Packing structure of 5h in a unit cell.

4. References:

1. (a) Verma, S.; Sharon, S. Intramolecular cyclization to fused pyranoisoxazoles and molecular packing motifs identification through X-ray structural studies. *J. Indian Chem. Soc.,* **2021**, *98*, 100135; (b) Shaw, R.; Pratap, R. A Green and Base-Free Arylation of Thiomethylated 2-pyranones and Ketene Dithioacetals via Liebeskind-Srogl Coupling in Water. *Asian J. Org. Chem.*, **2022**, *11*, e202200078.
2. Dolomanov, O.V., Bourhis, L.J., Gildea, R.J, Howard, J.A.K. & Puschmann, H. (2009), J. Appl. Cryst. 42, 339-341.
3. Sheldrick, G.M. (2015). Acta Cryst. A71, 3-8.
4. Bourhis, L.J., Dolomanov, O.V., Gildea, R.J., Howard, J.A.K., Puschmann, H. (2015). Acta Cryst. A71, 59-75.

5. ^{1}H and ^{13}C NMR spectra of products

3a. 5-(methylthio)-[1,1'-biphenyl]-3-ol: ^{1}H and ^{13}C NMR

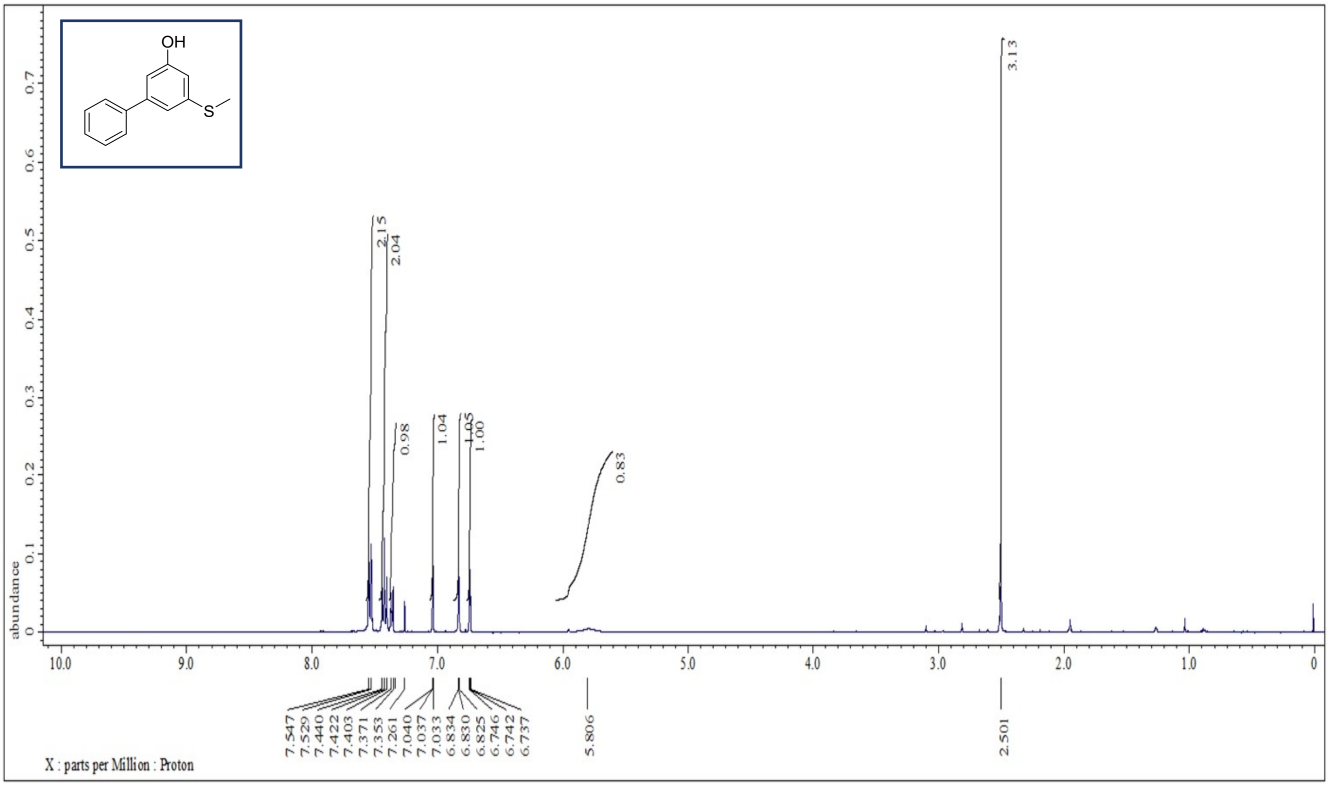

Expanded view for aromatic and phenolic proton.

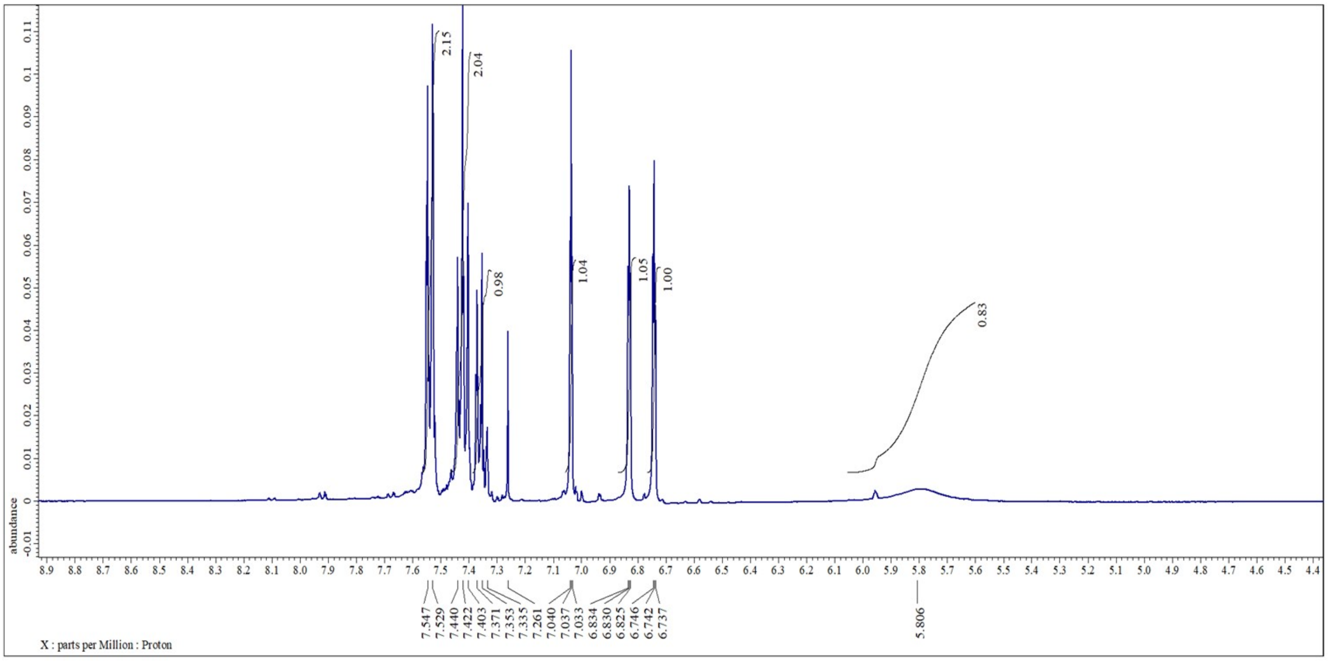

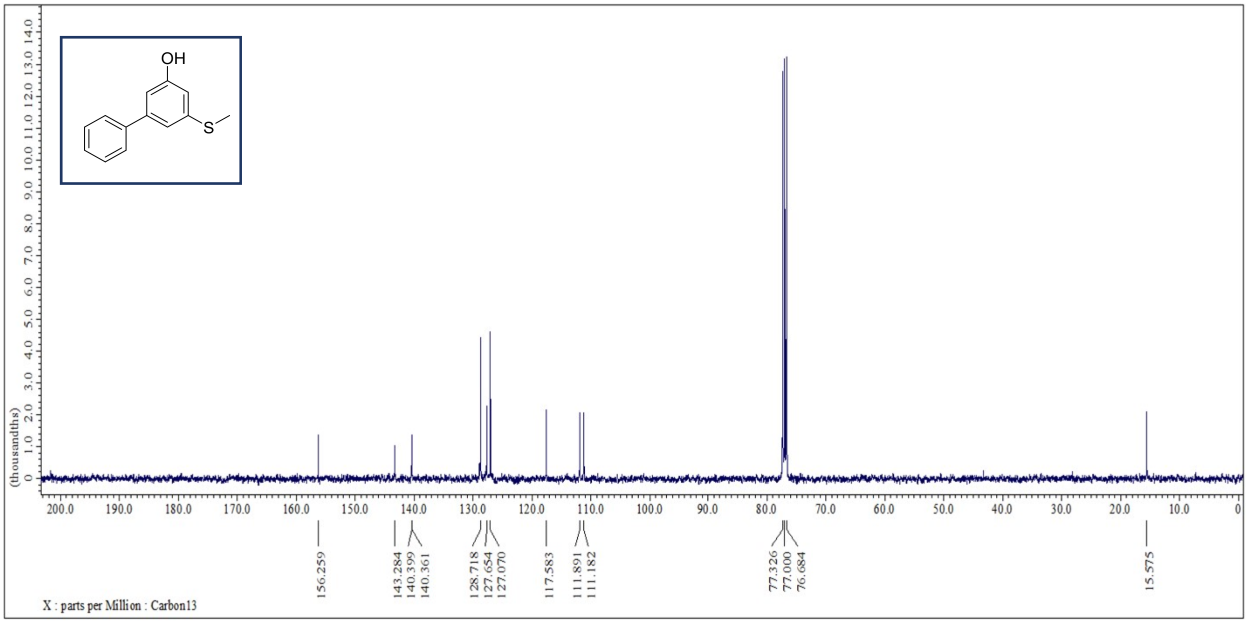

OH
S
(thousandths)
X : parts per Million : Carbon13
156.259
143.284
140.399
140.361
128.718
127.654
127.070
117.583
111.891
111.182
77.326
77.000
76.684
15.575

3b. 4'-methyl-5-(methylthio)-[1,1'-biphenyl]-3-ol: ^1H and ^{13}C NMR

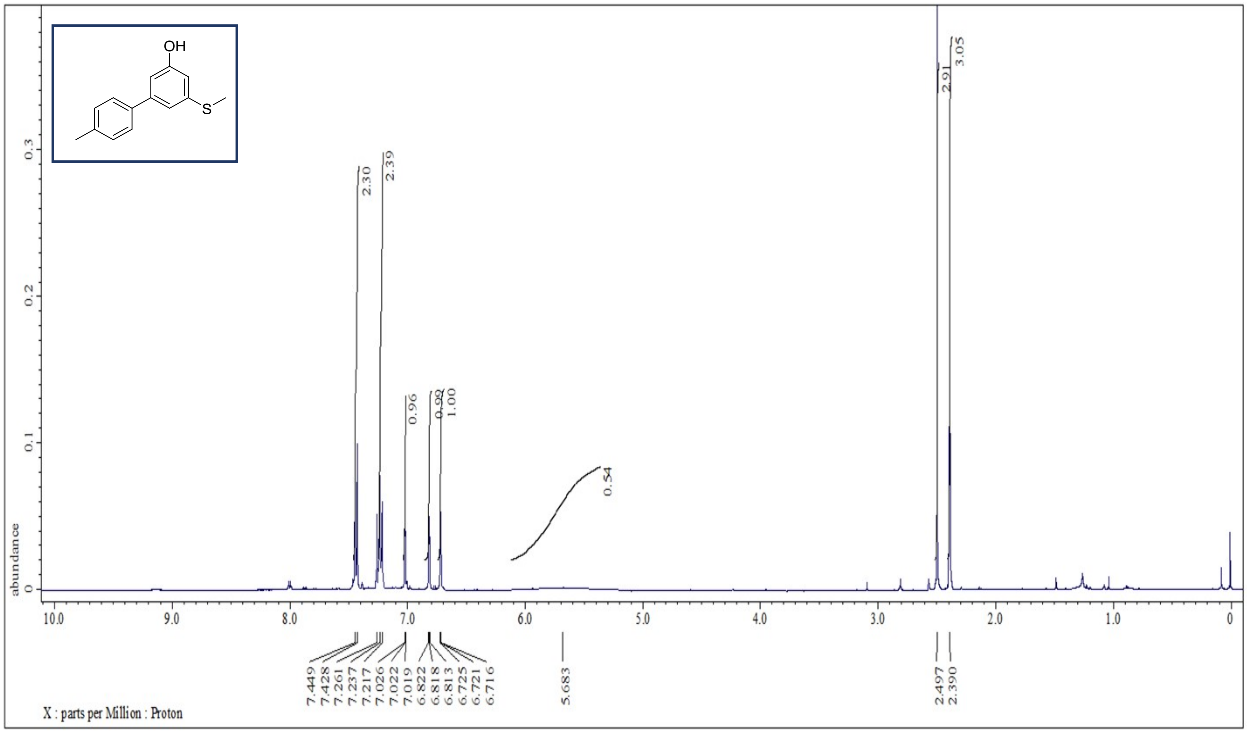

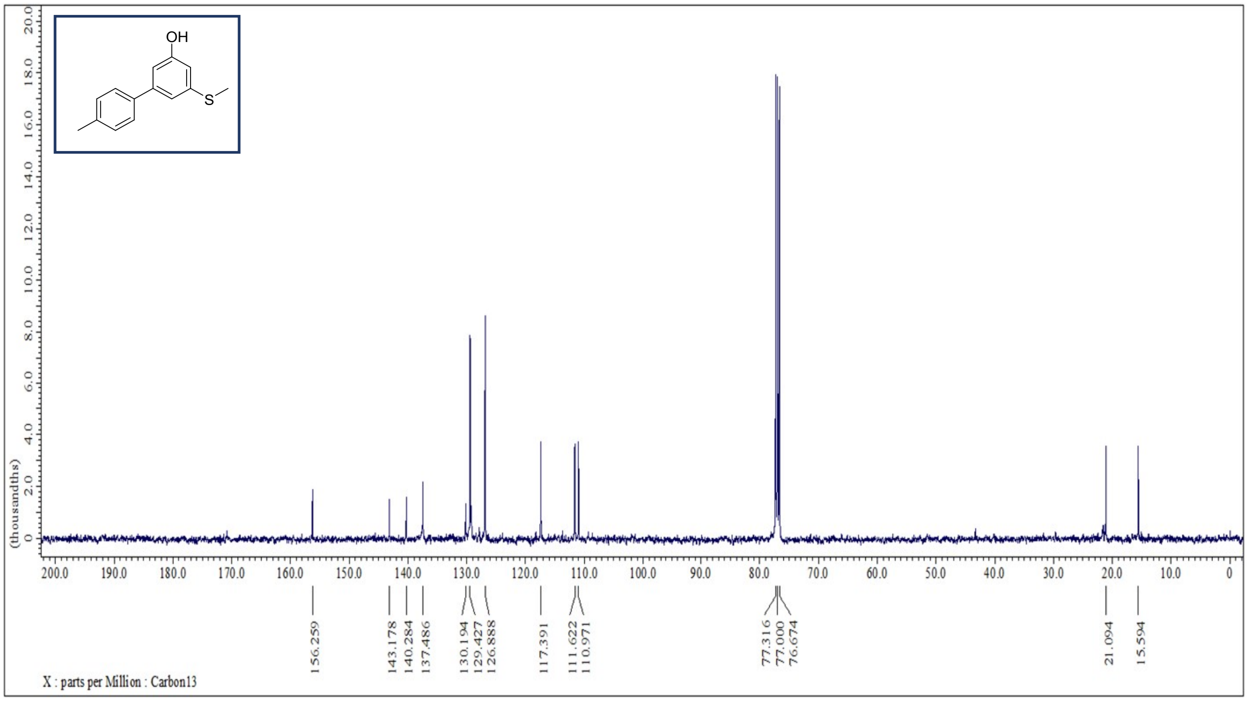

3c. 4'-methoxy-5-(methylthio)-[1,1'-biphenyl]-3-ol: ^{1}H and ^{13}C NMR

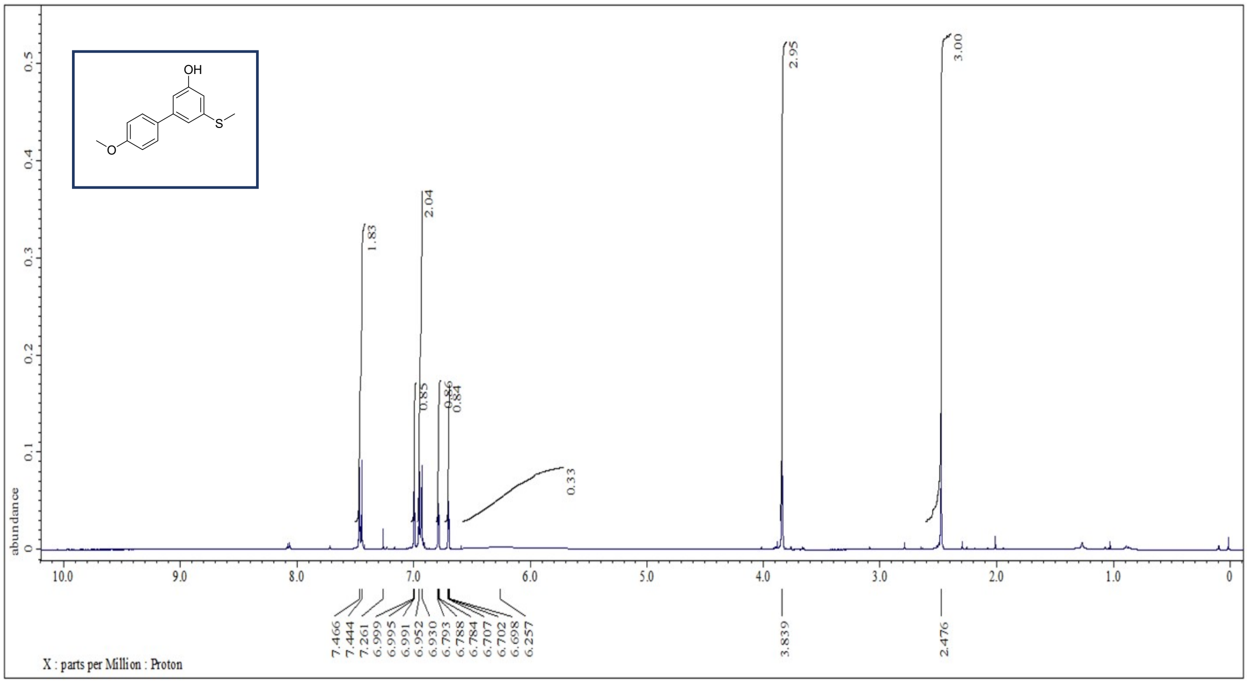

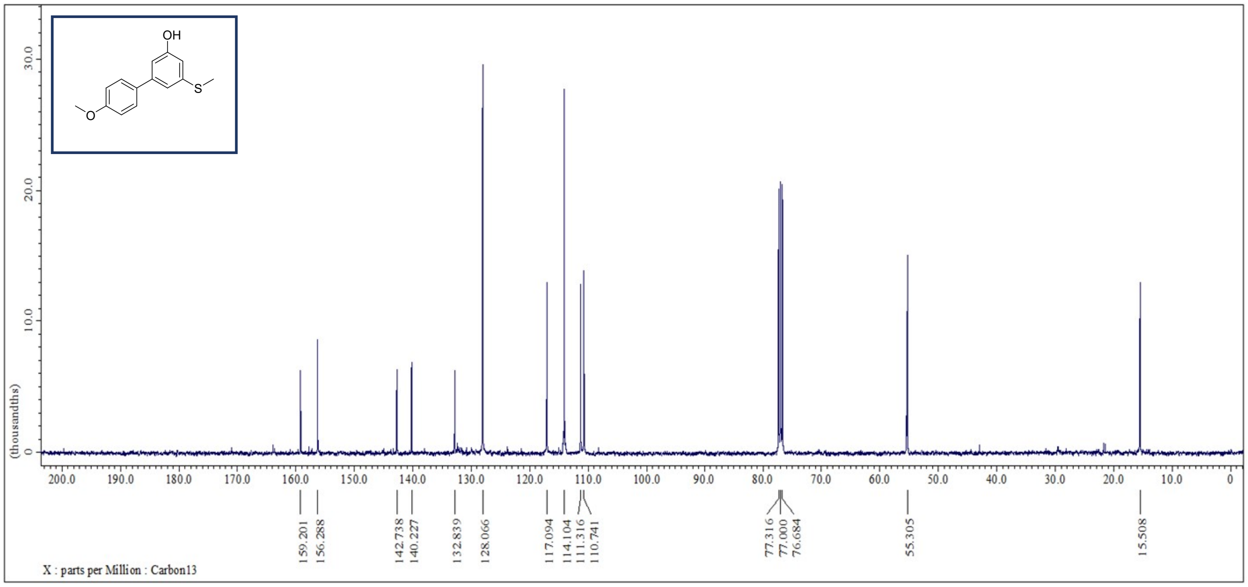

3d. 4'-fluoro-5-(methylthio)-[1,1'-biphenyl]-3-ol: ^{1}H and ^{13}C NMR

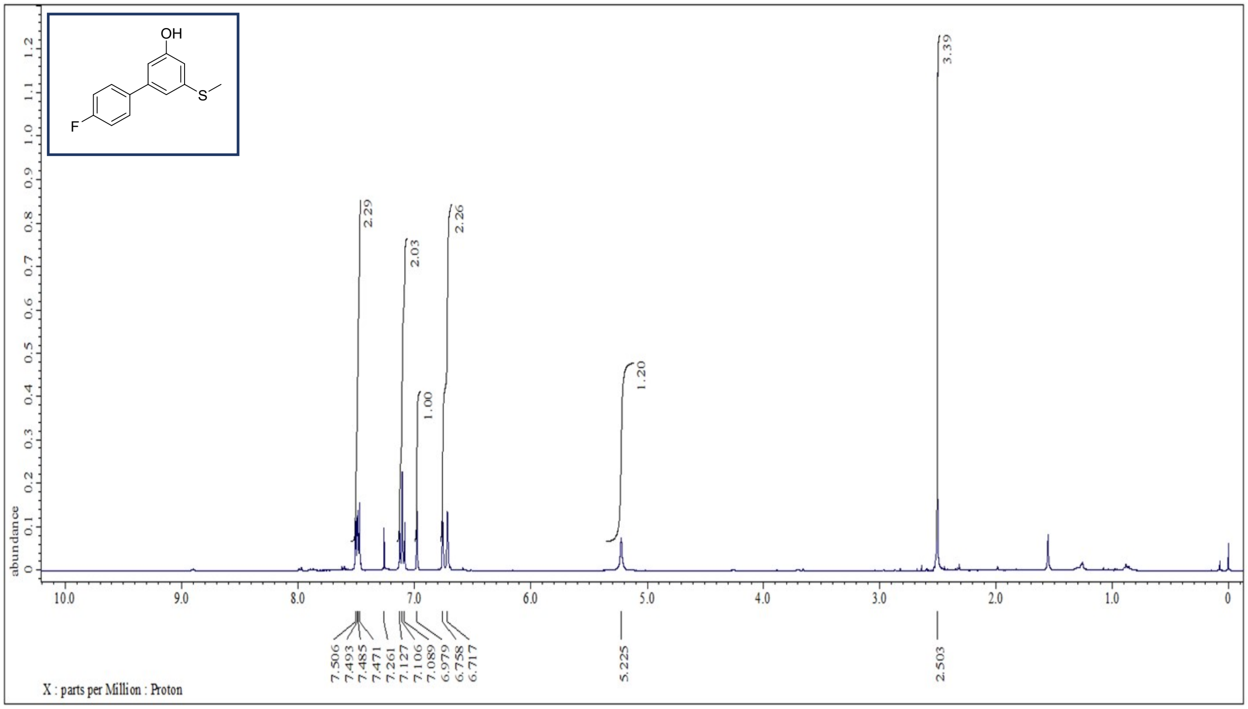

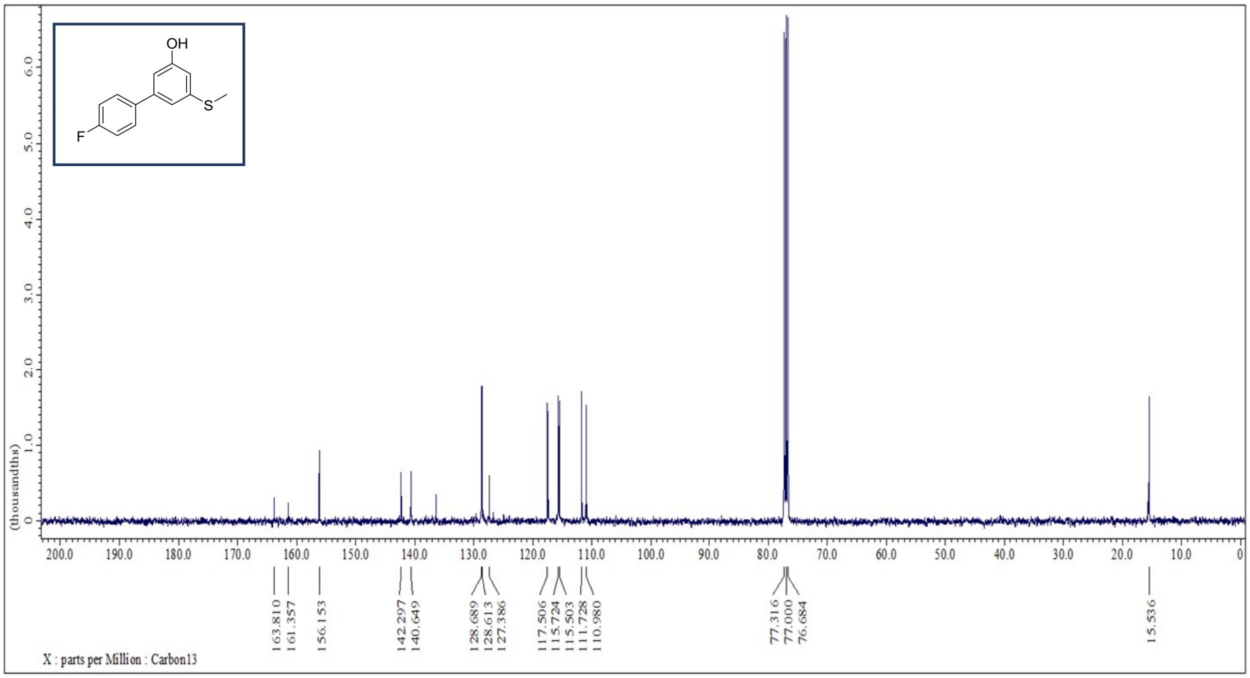

3e. 4'-chloro-5-(methylthio)-[1,1'-biphenyl]-3-ol: ^{1}H and ^{13}C NMR

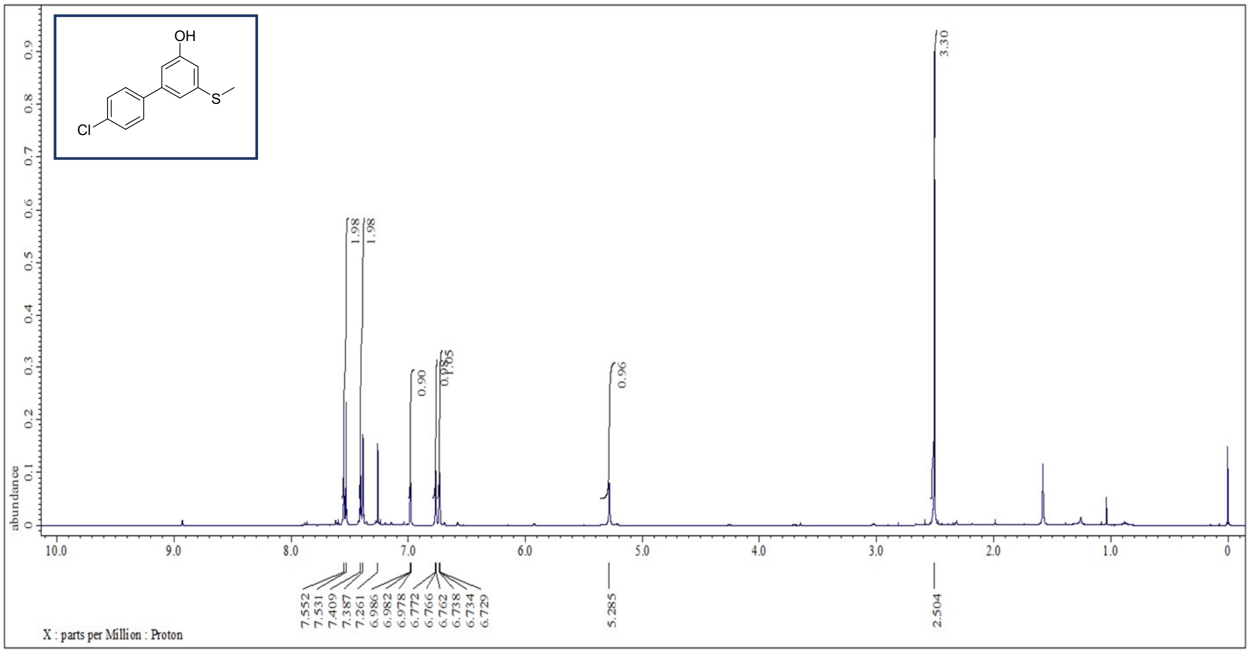

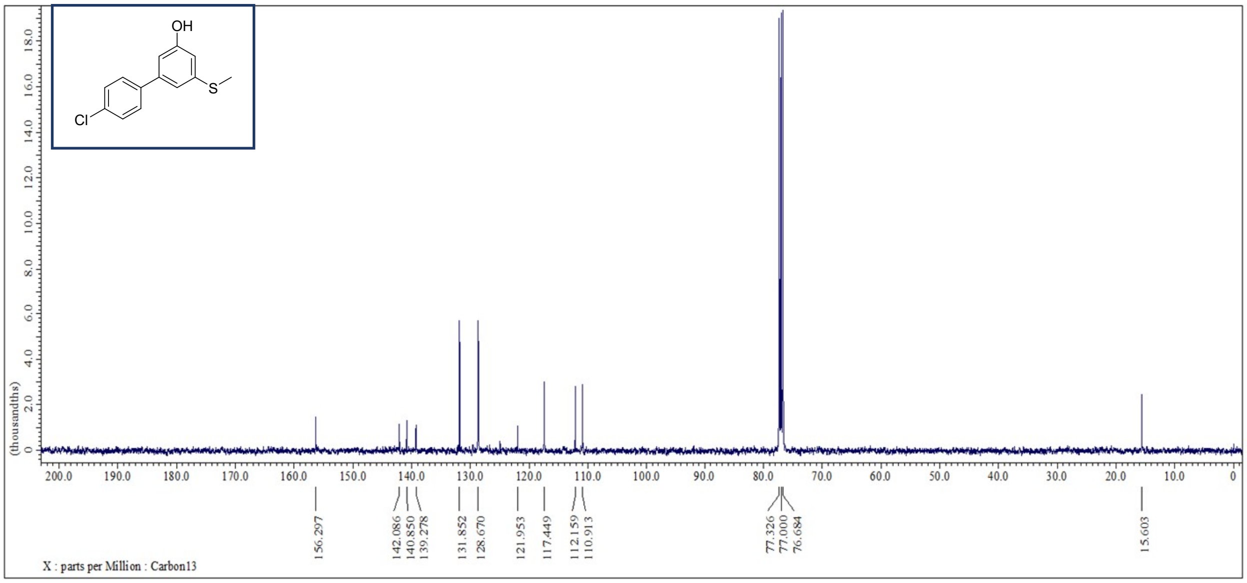

3f. 4'-bromo-5-(methylthio)-[1,1'-biphenyl]-3-ol: ^{1}H and ^{13}C NMR

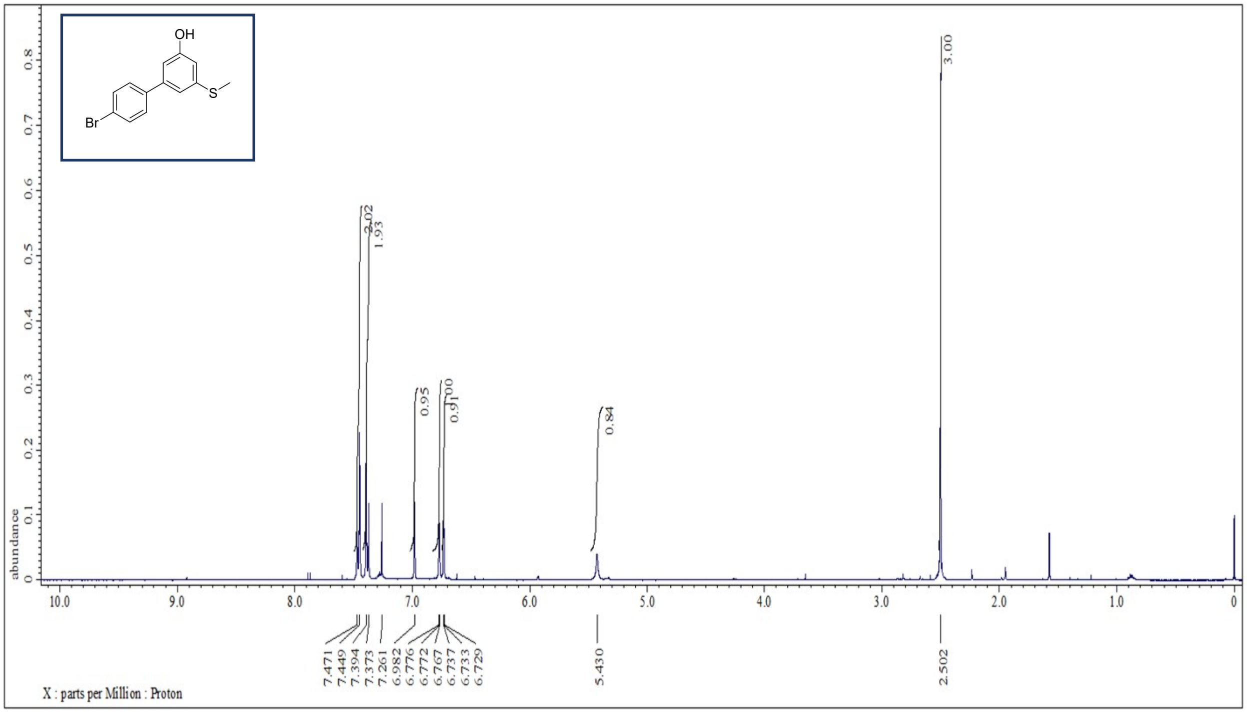

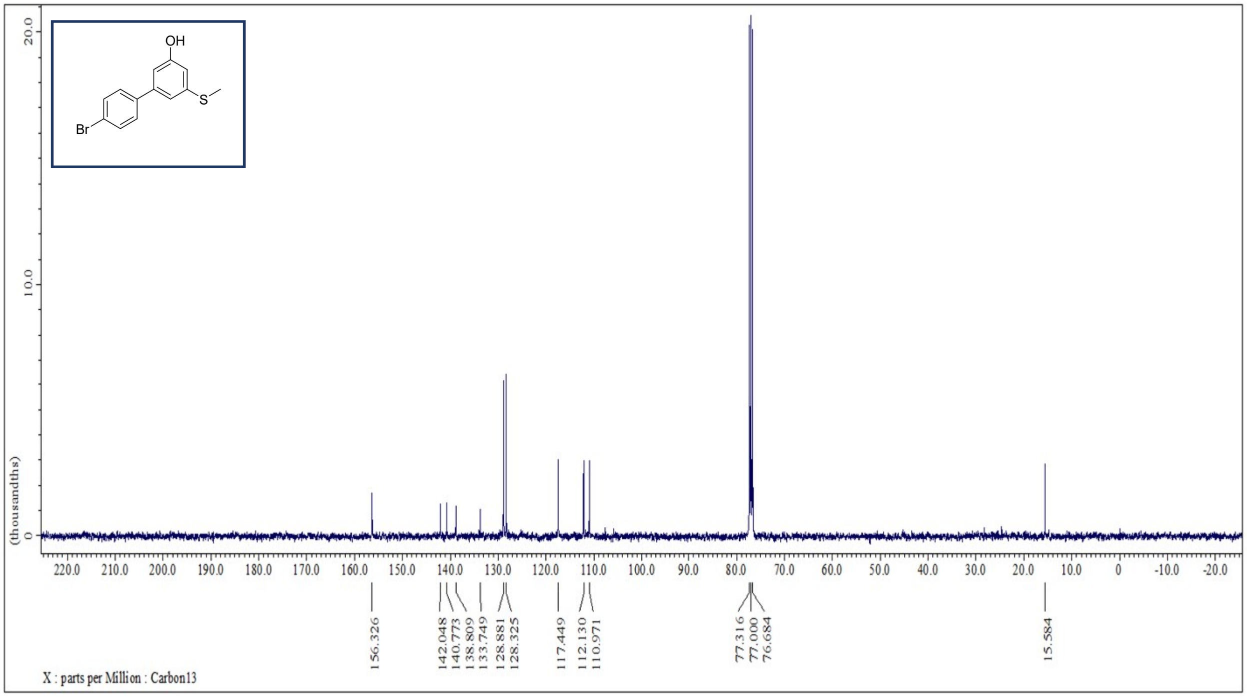

3g. 2'-chloro-5-(methylthio)-[1,1'-biphenyl]-3-ol: ^{1}H and ^{13}C NMR

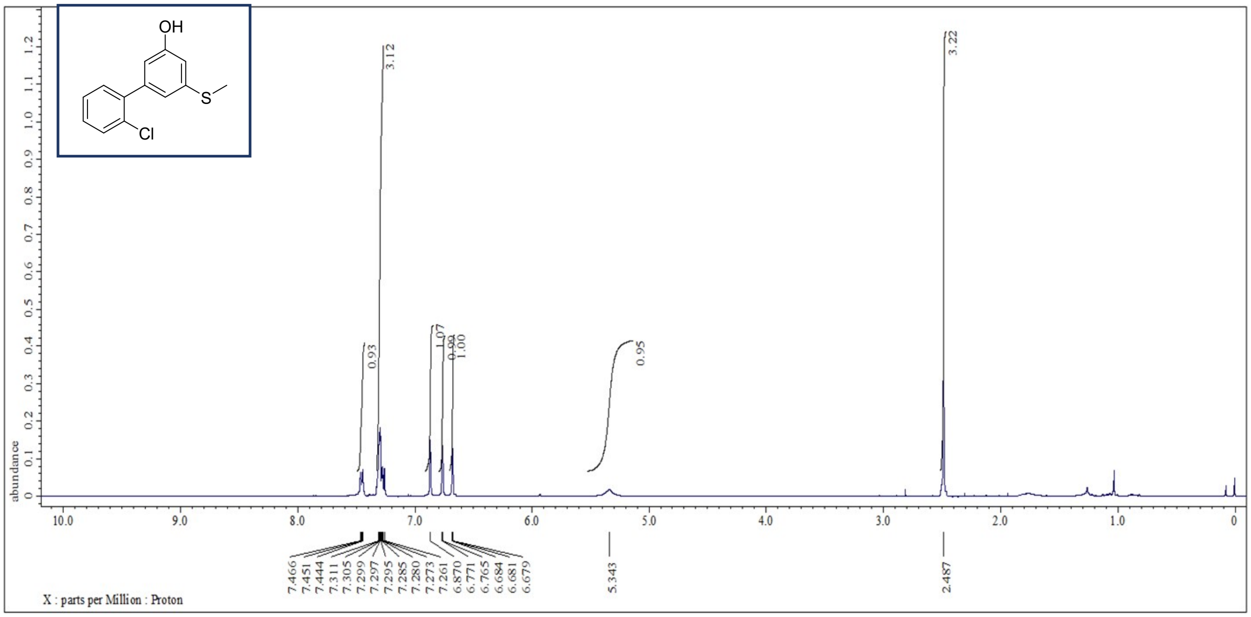

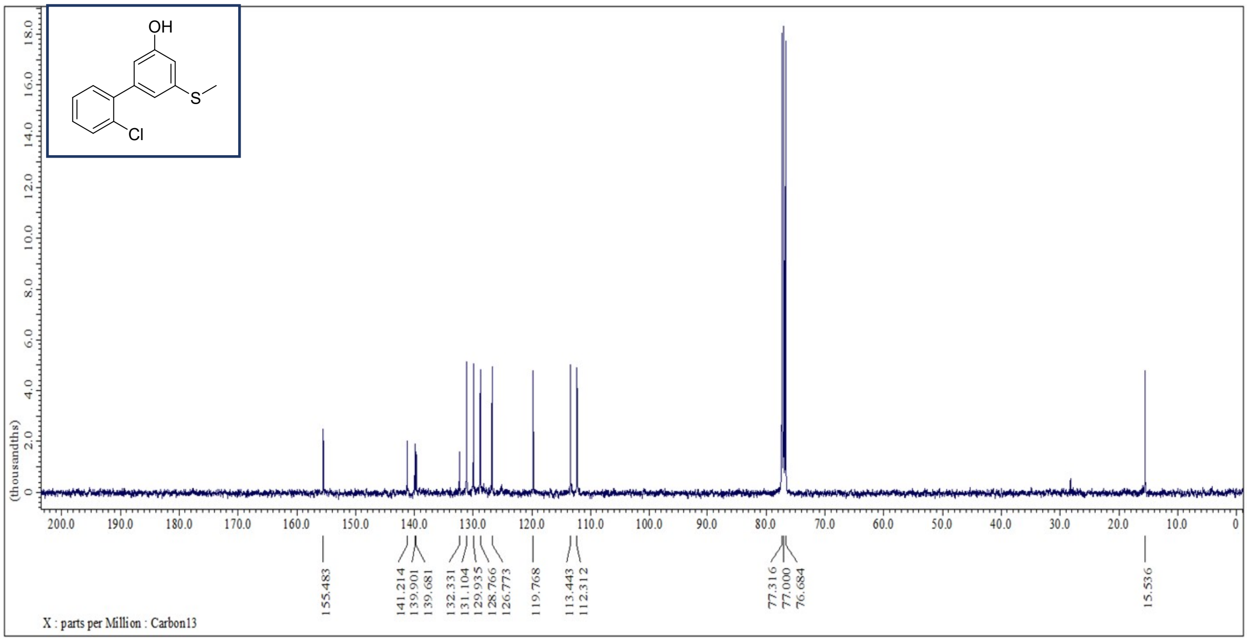

3h. 2'-bromo-5-(methylthio)-[1,1'-biphenyl]-3-ol: ^{1}H and ^{13}C NMR

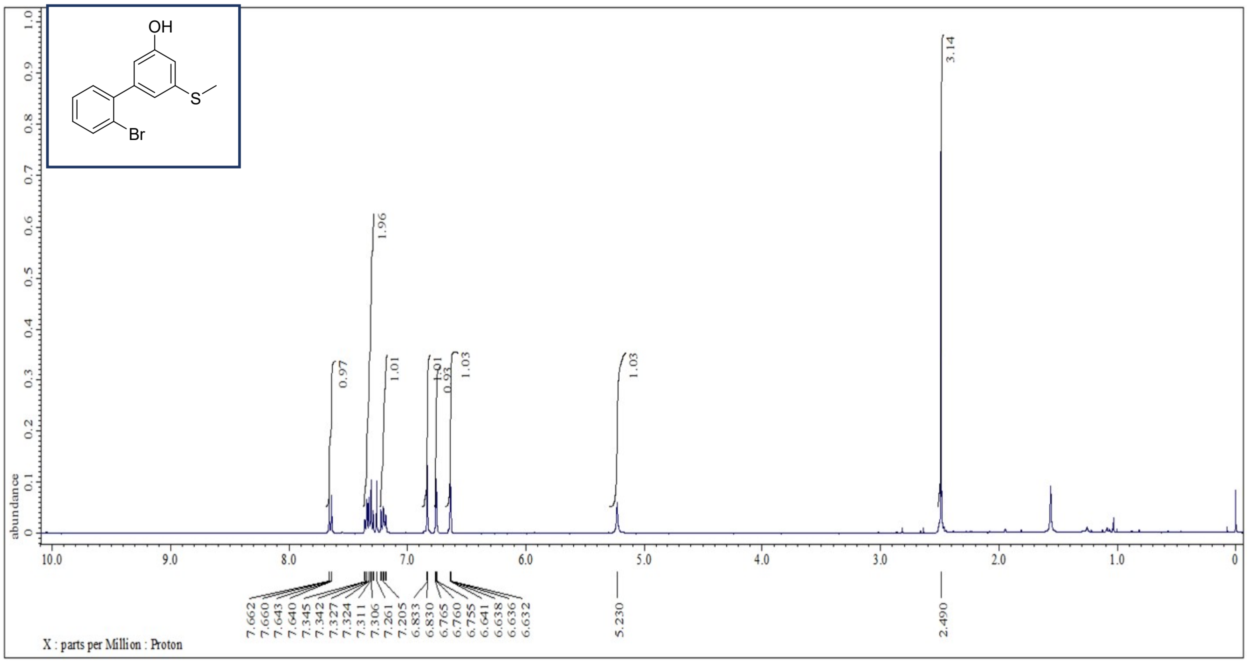

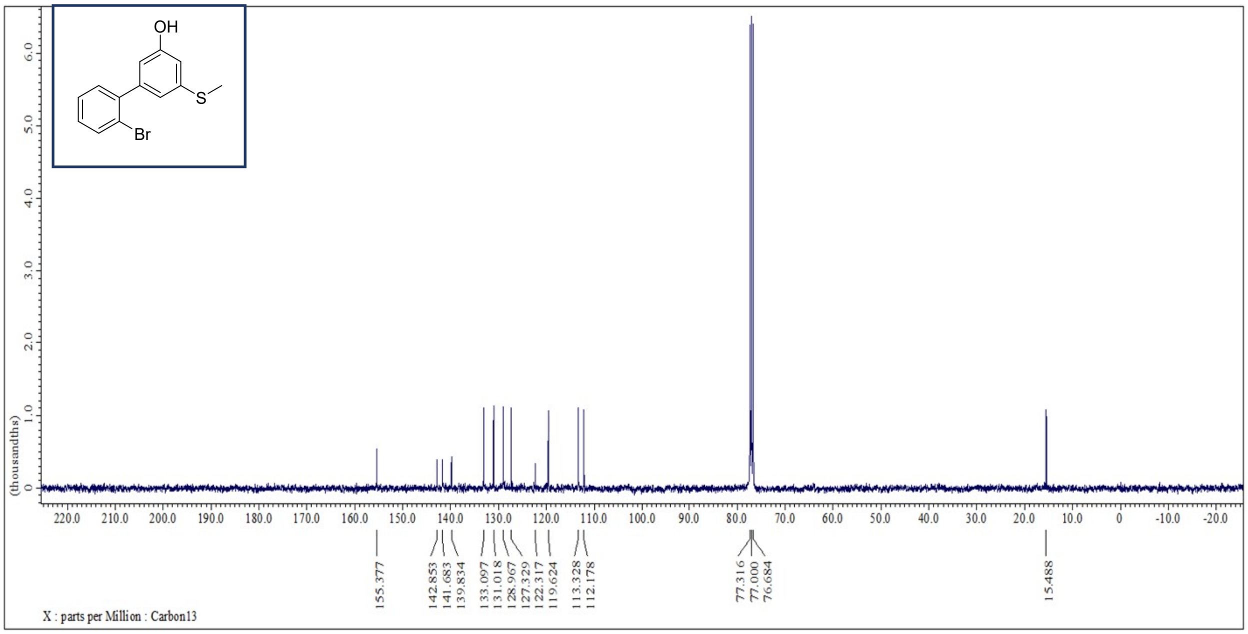

3i. 2',4'-dichloro-5-(methylthio)-[1,1'-biphenyl]-3-ol: ^{1}H and ^{13}C NMR

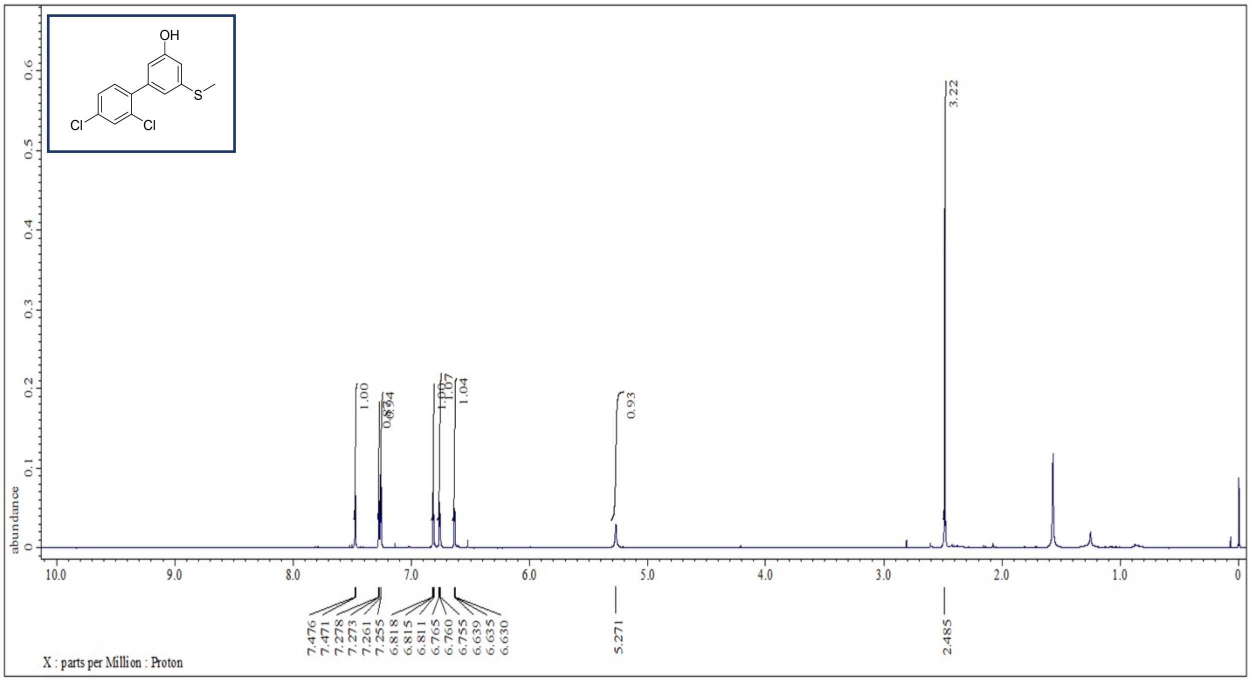

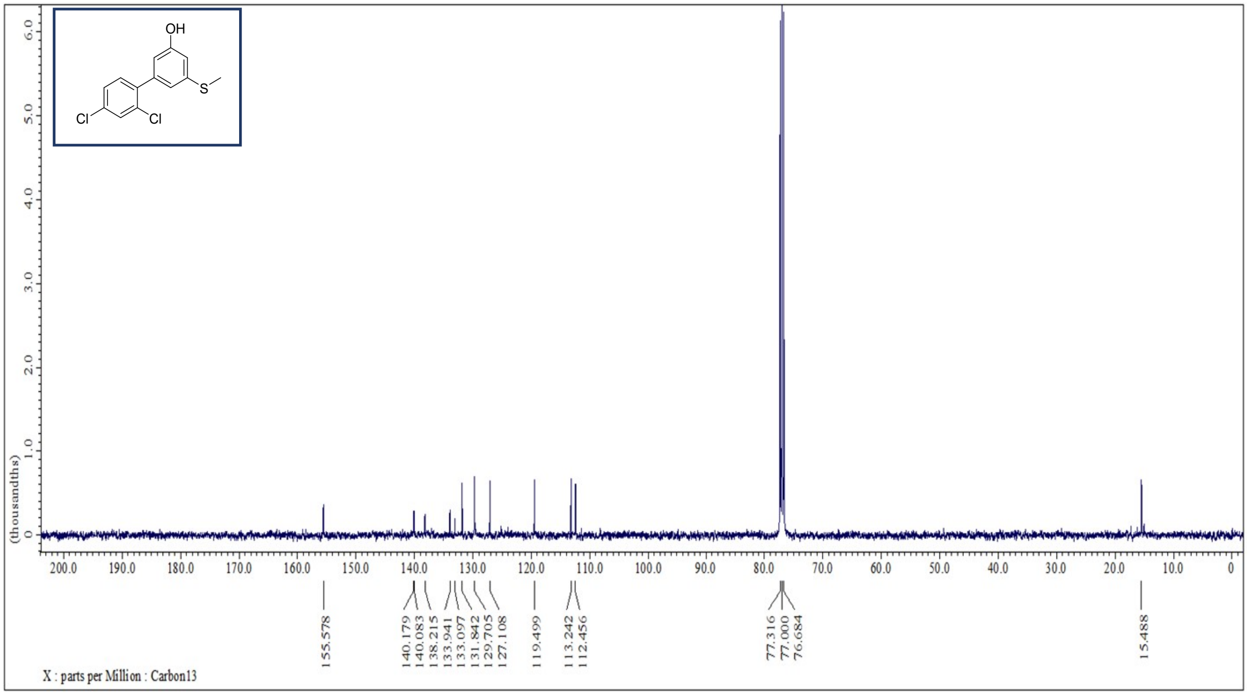

3j. 3-(methylthio)-5-(naphthalen-1-yl)phenol: ^{1}H and ^{13}C NMR

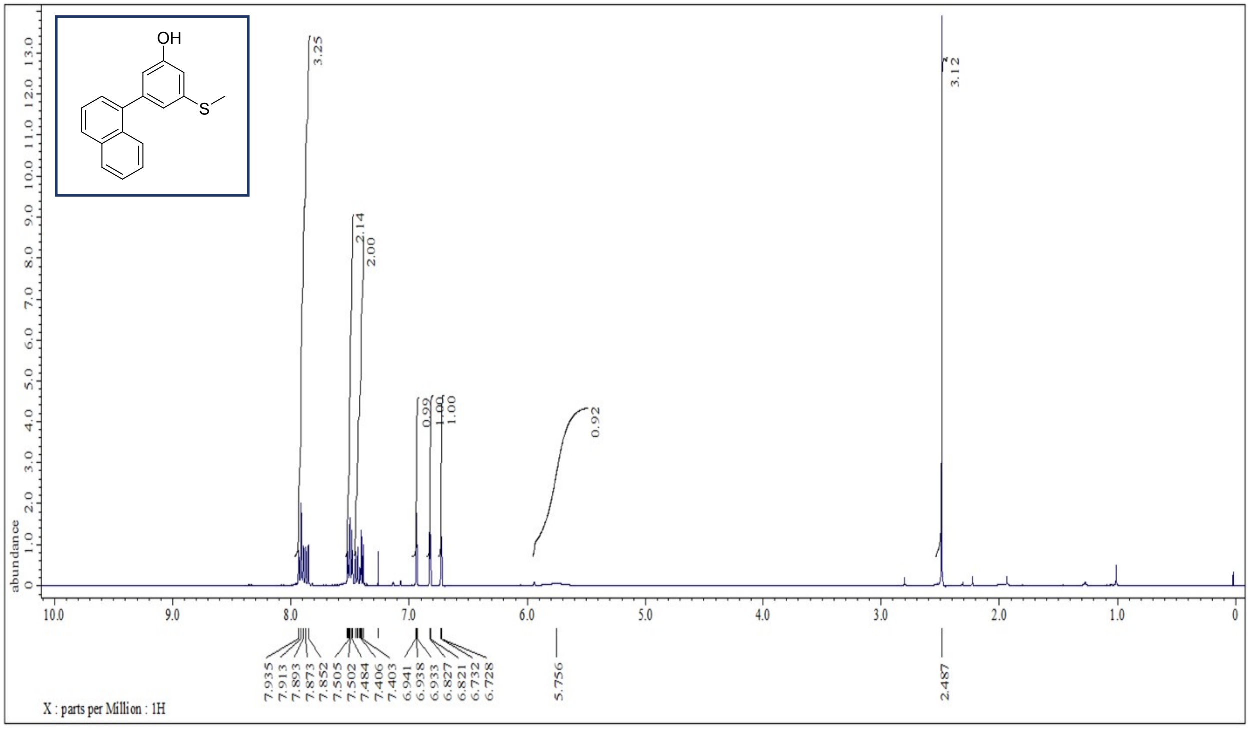

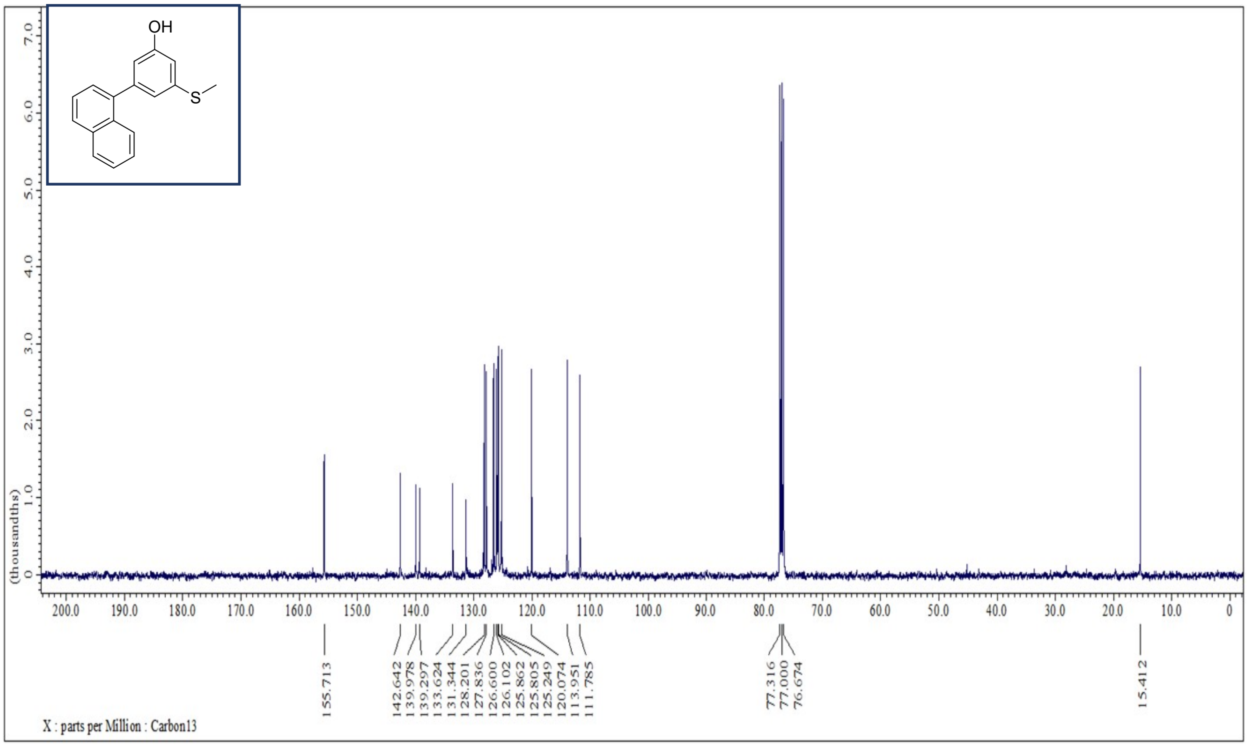

3k. 3-(methylthio)-5-(naphthalen-2-yl)phenol: ^{1}H and ^{13}C NMR

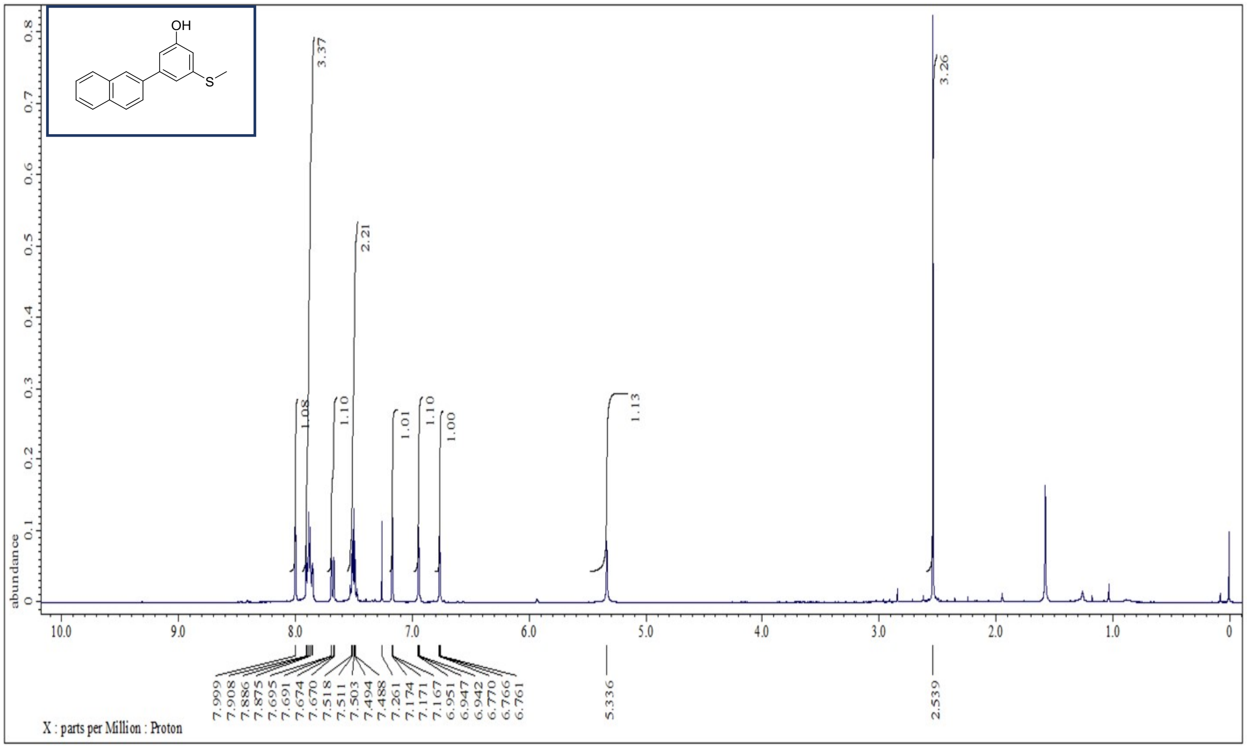

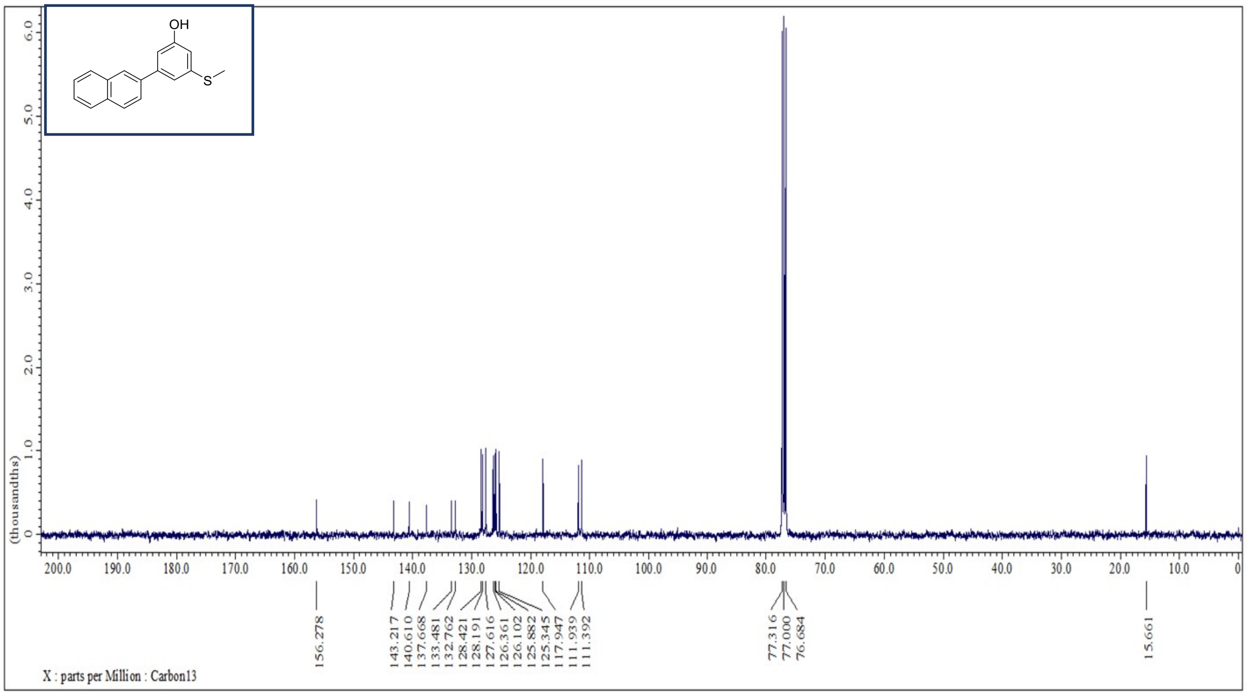

3l. 3-(furan-2-yl)-5-(methylthio)phenol: ^{1}H and ^{13}C NMR

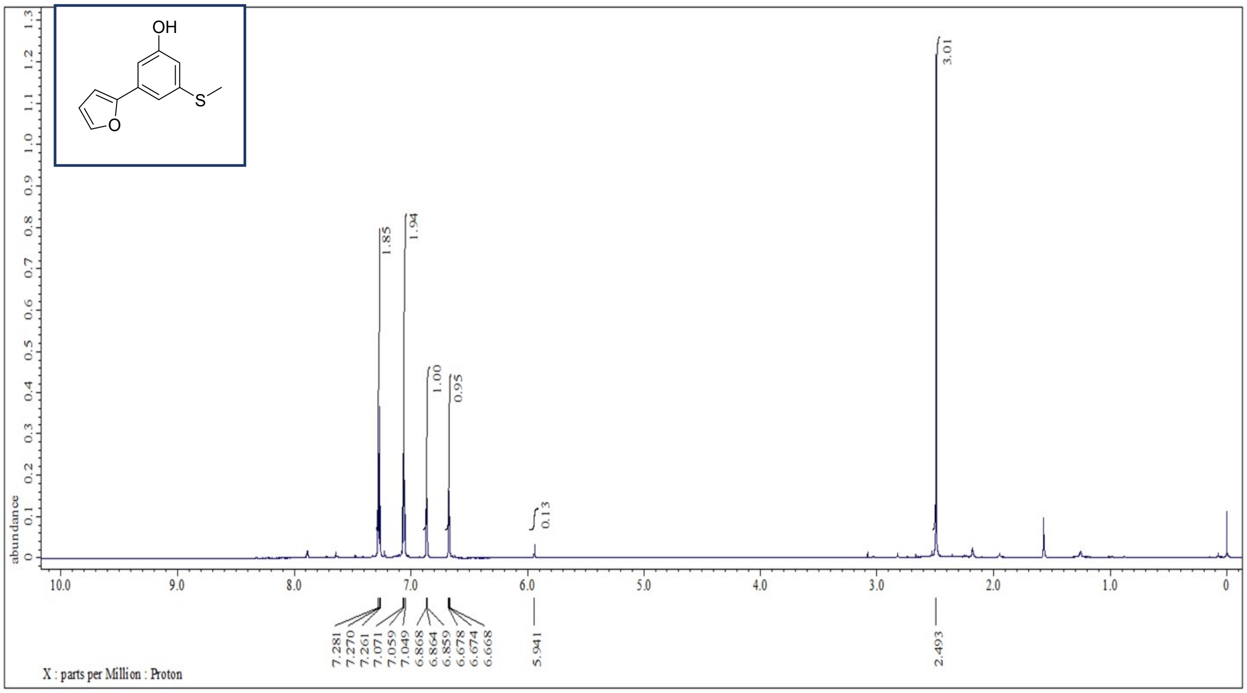

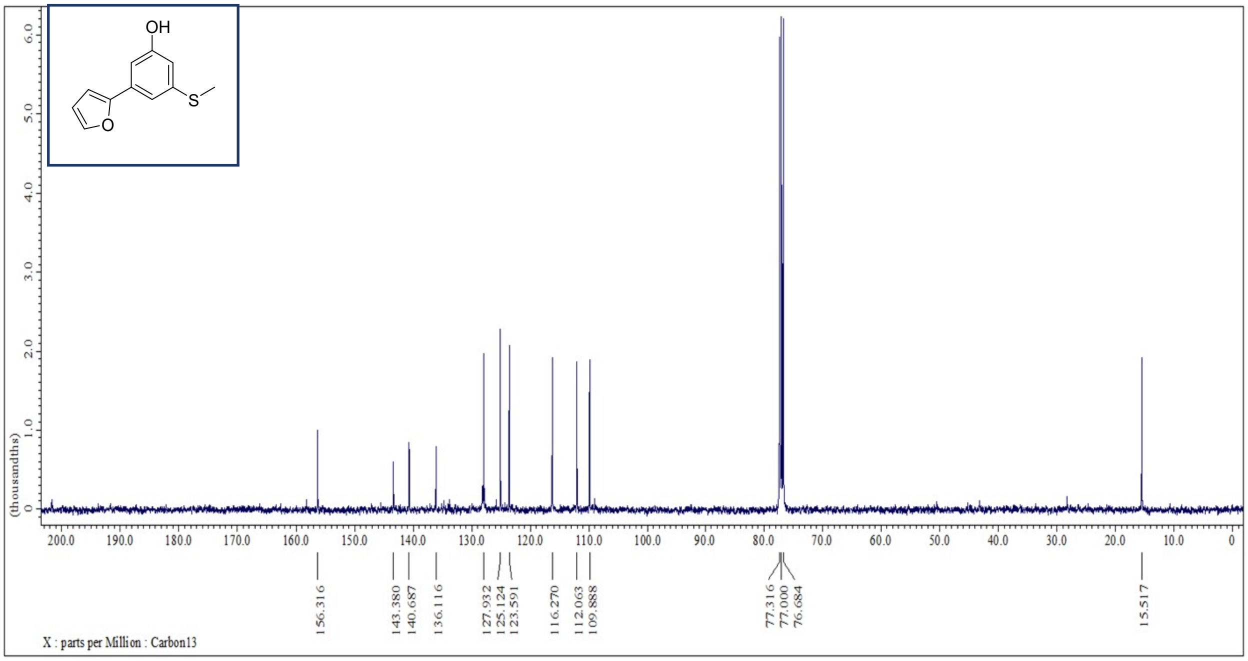

3m. 3-(methylthio)-5-(thiophen-2-yl)phenol: ^{1}H and ^{13}C NMR

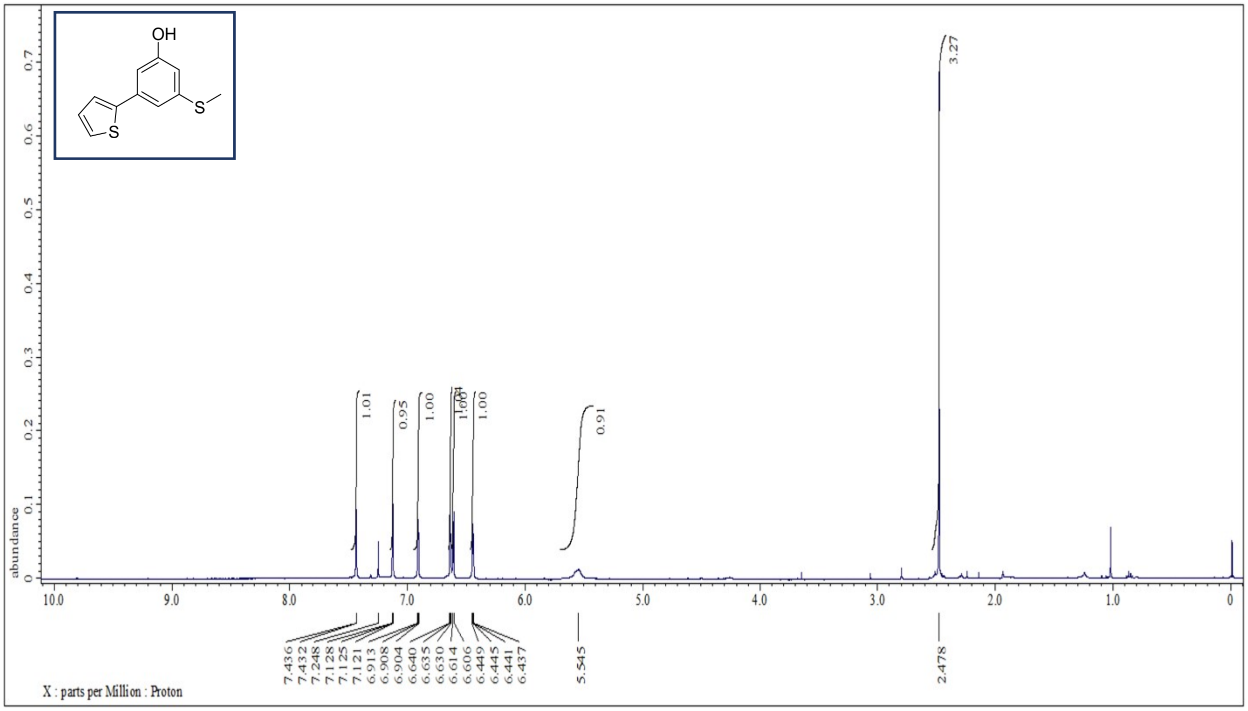

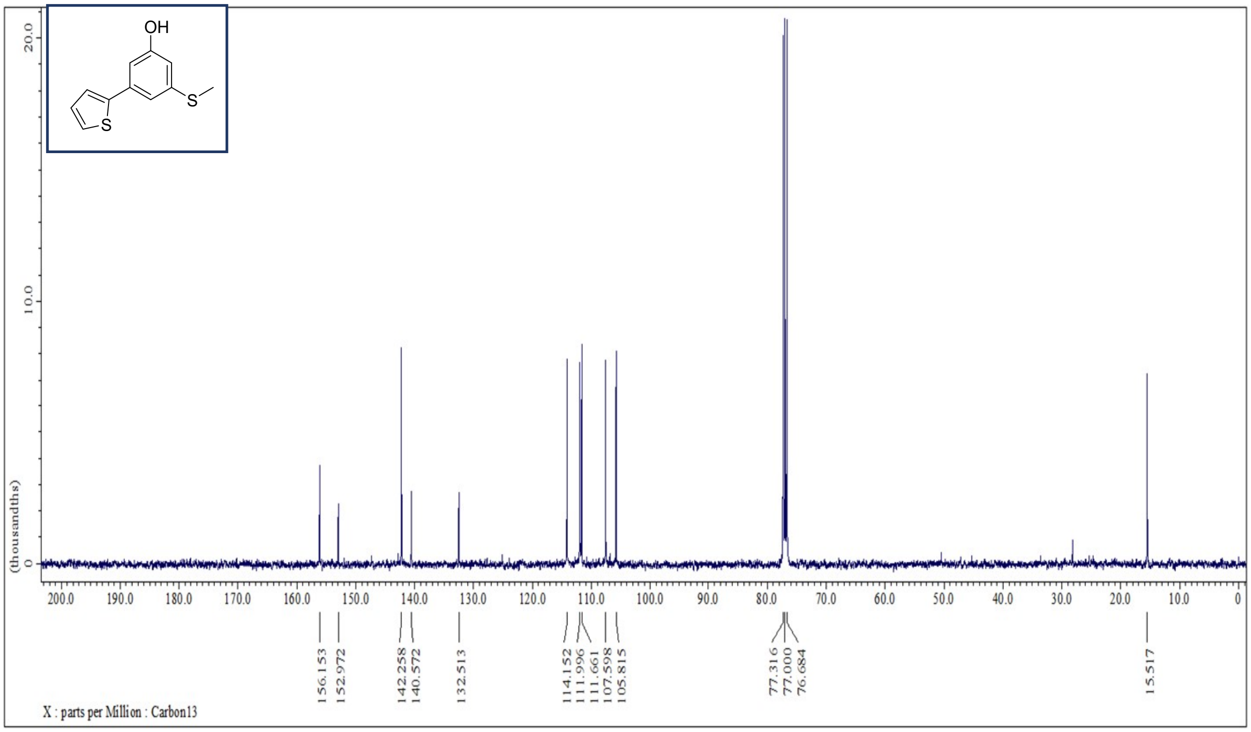

5a. 2,4-dimethyl-5-(methylthio)-[1,1'-biphenyl]-3-ol: ^{1}H and ^{13}C NMR

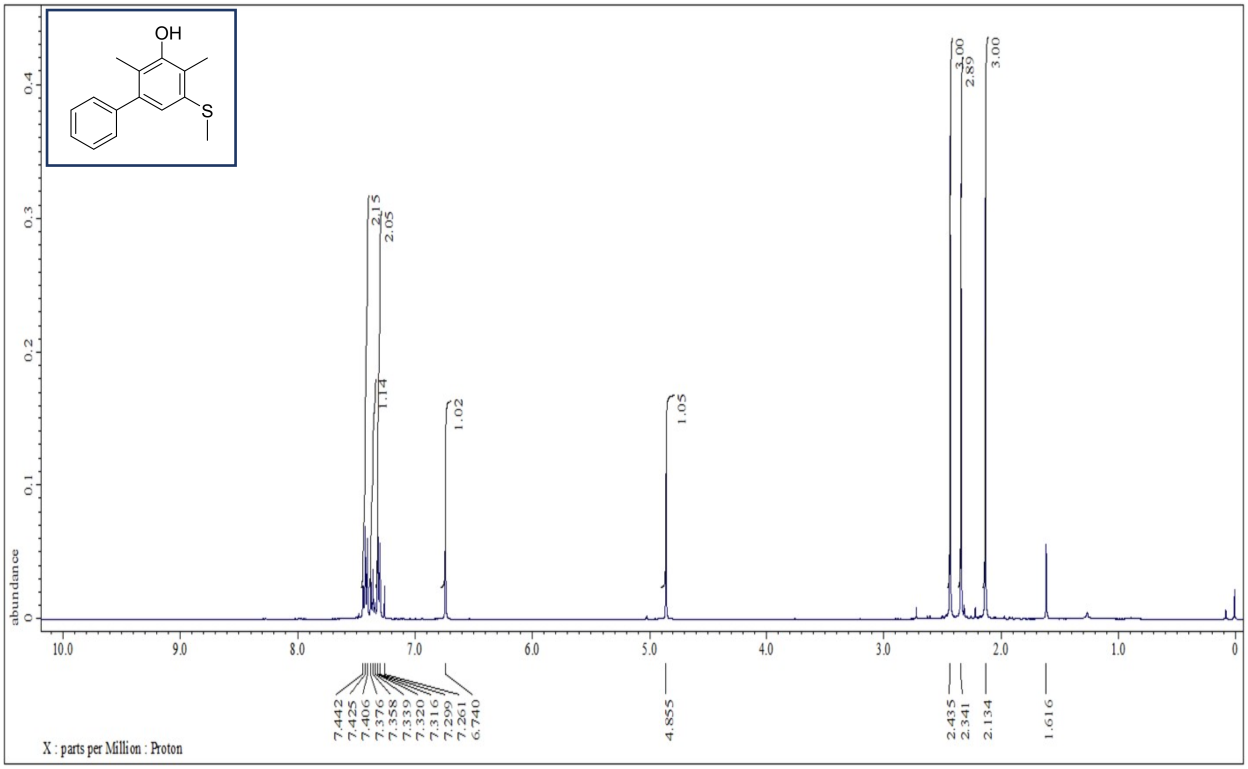

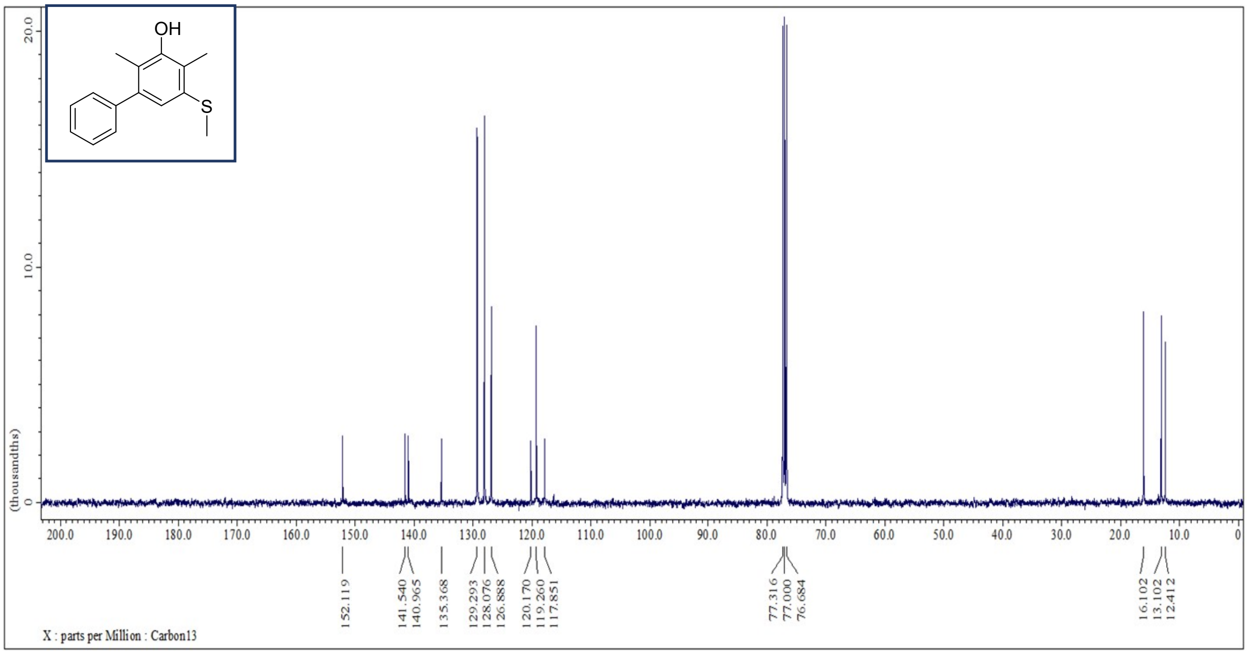

5b. 2'-chloro-2,4-dimethyl-5-(methylthio)-[1,1'-biphenyl]-3-ol: ^{1}H and ^{13}C NMR

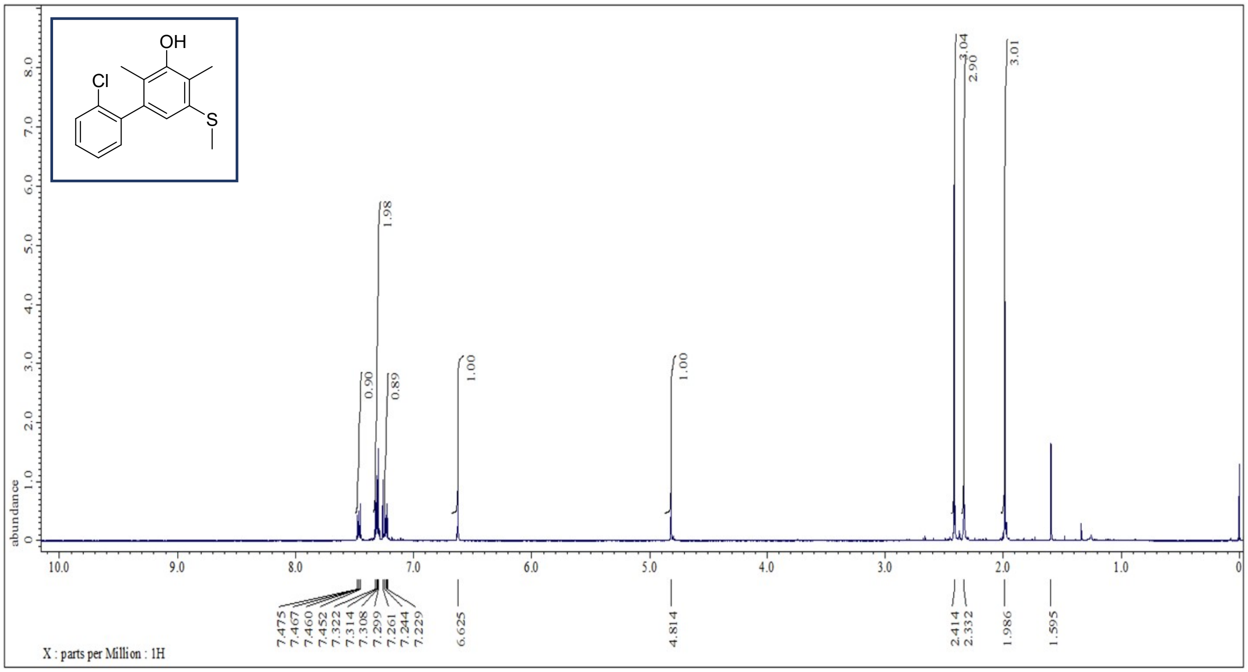

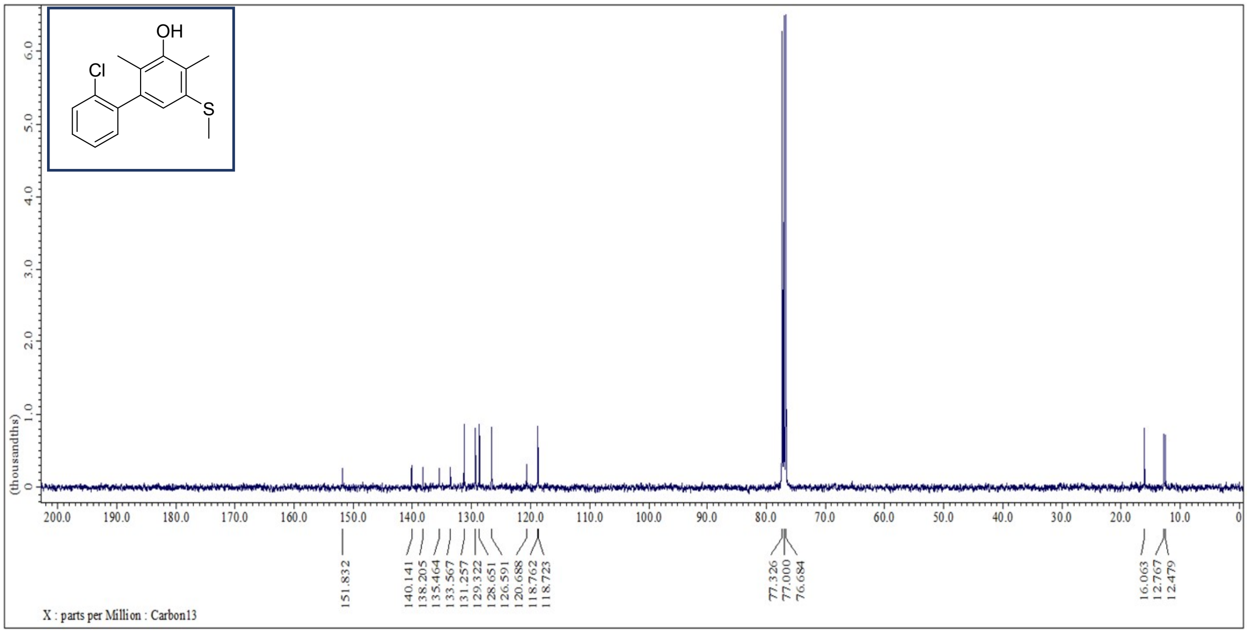

5c. 2'-bromo-2,4-dimethyl-5-(methylthio)-[1,1'-biphenyl]-3-ol: ^{1}H and ^{13}C NMR

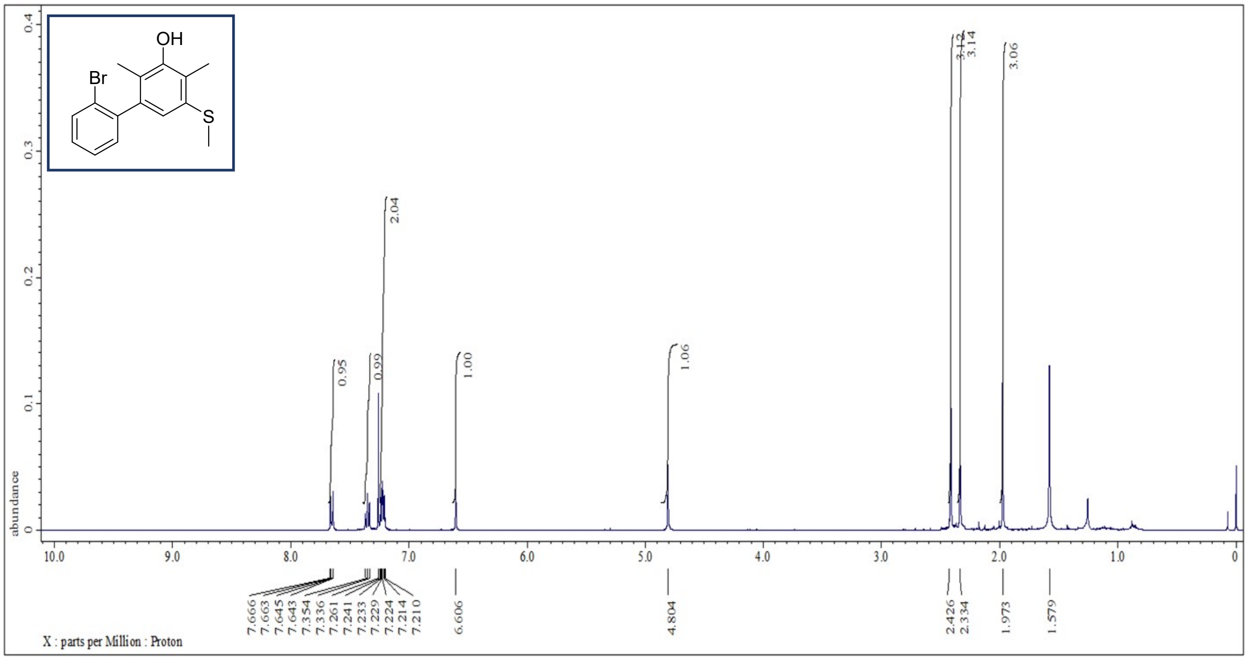

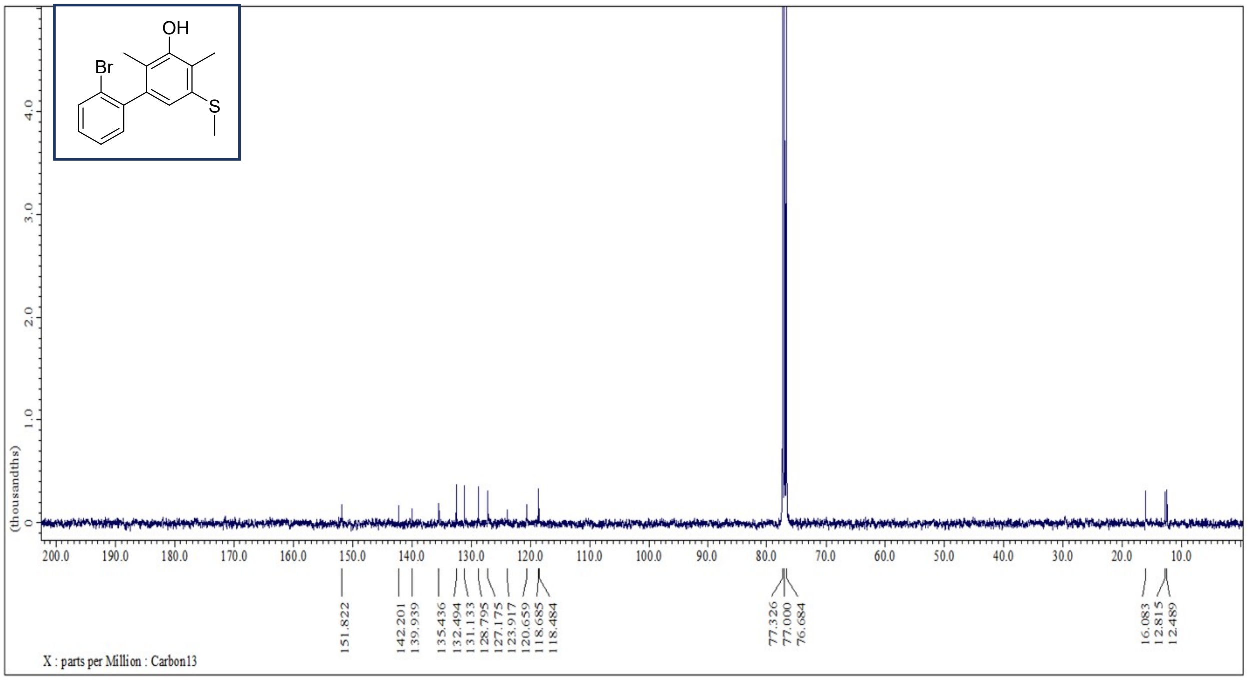

5d. 2,6-dimethyl-3-(methylthio)-5-(naphthalen-2-yl)phenol: ^{1}H and ^{13}C NMR

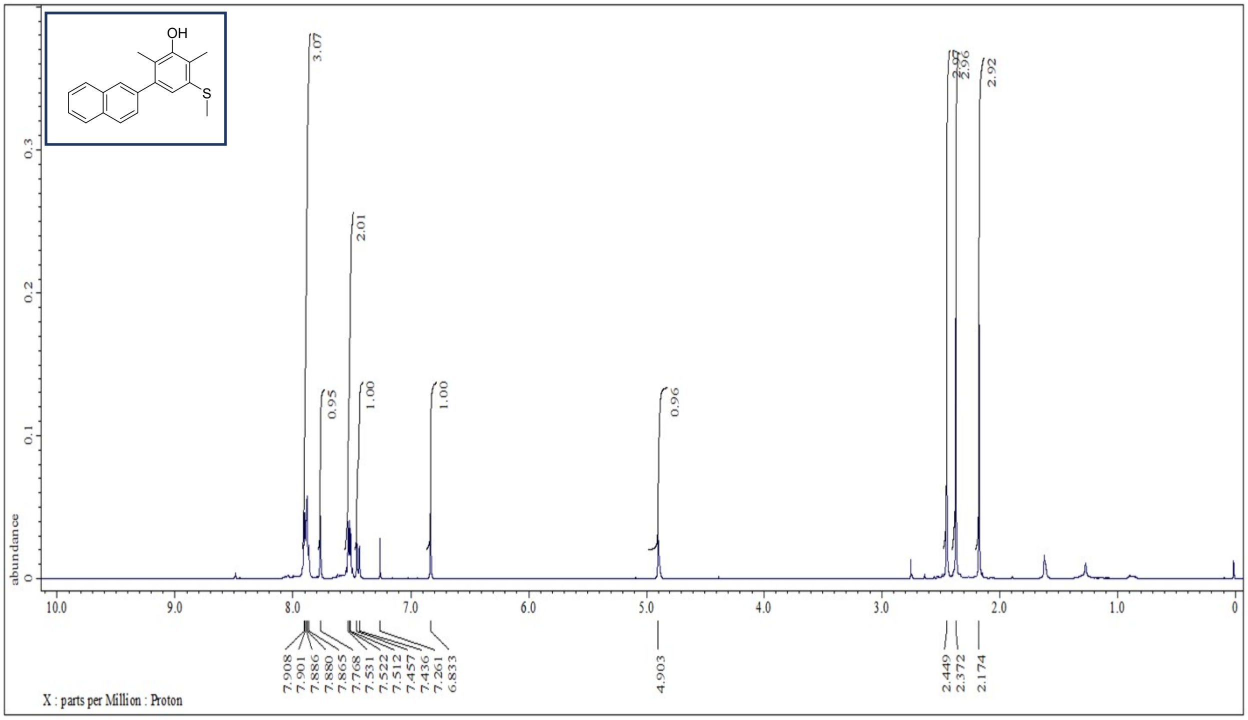

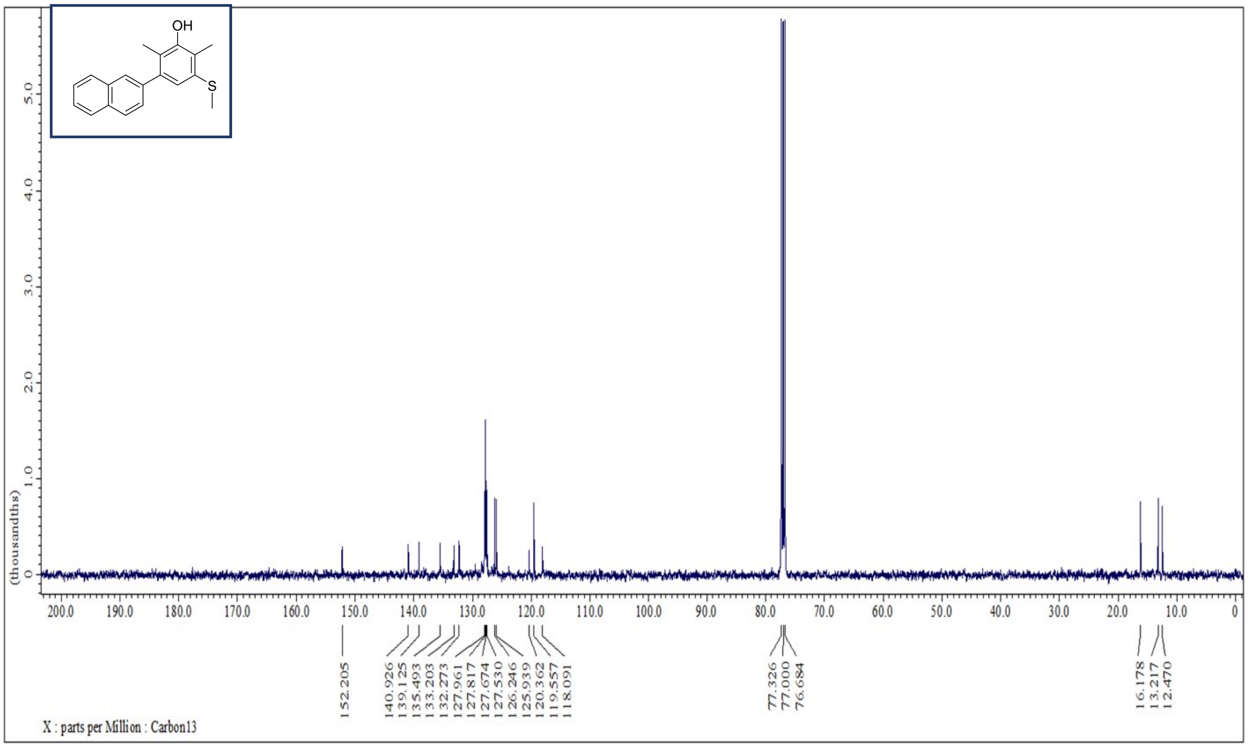

5e. 2,6-dimethyl-3-(methylthio)-5-(thiophen-2-yl)phenol: ^{1}H and ^{13}C NMR

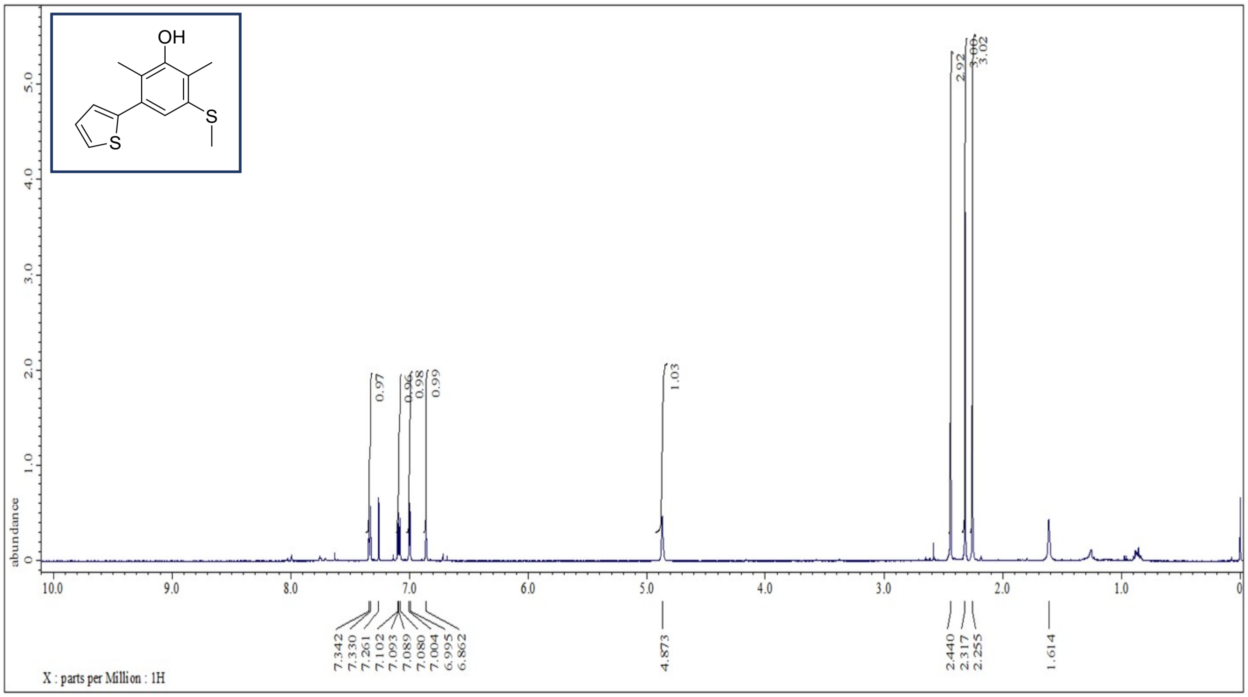

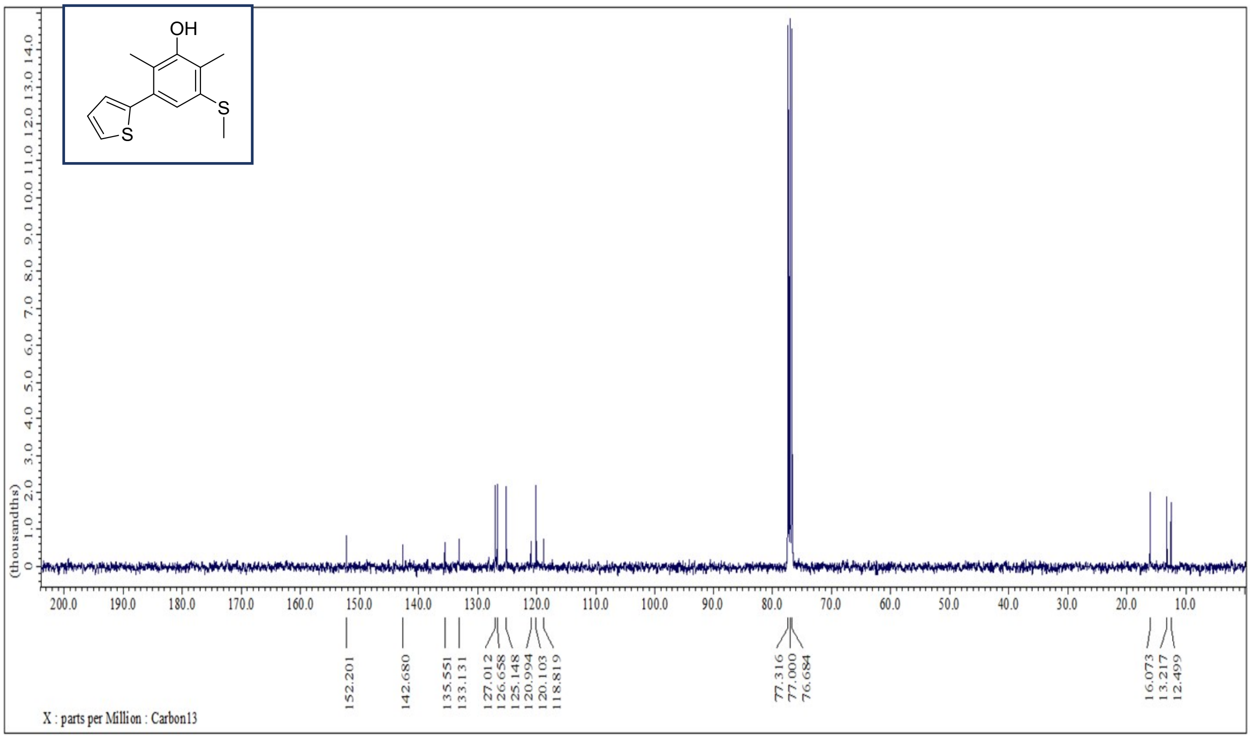

5fa and 5fb. 4-methyl-5-(methylthio)-[1,1'-biphenyl]-3-ol and 2-methyl-5-(methylthio)-[1,1'-biphenyl]-3-ol: ^{1}H NMR (When prepared in NaH, 5fa:5fb = 10;3): ^{1}H and ^{13}C NMR

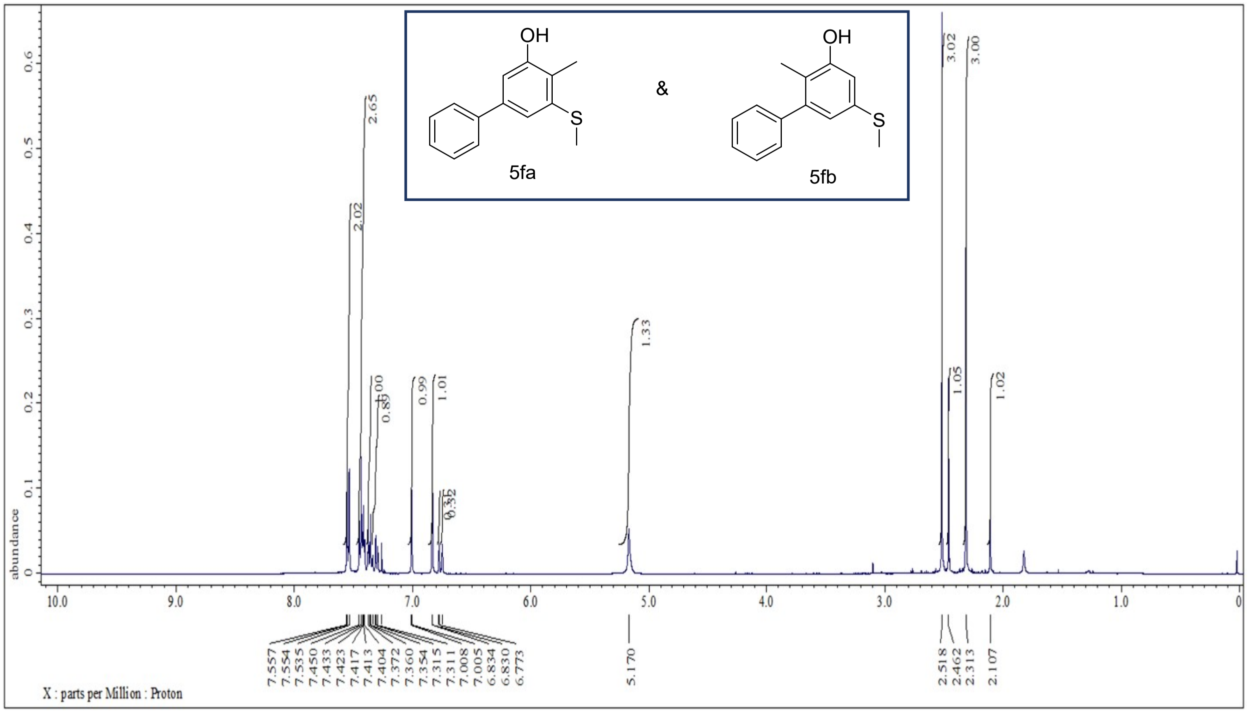

^{1}H NMR of 5fa and 5fb (When prepared in ^{t}BuOK, 5fa:5fb = 5;1)

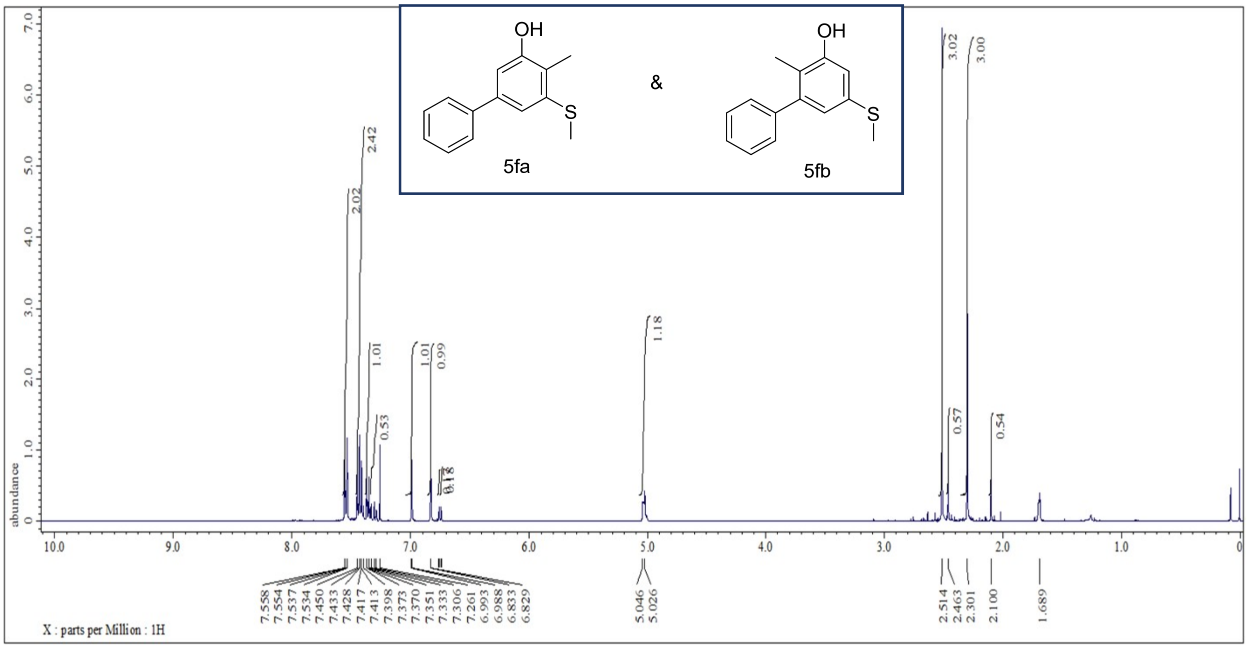

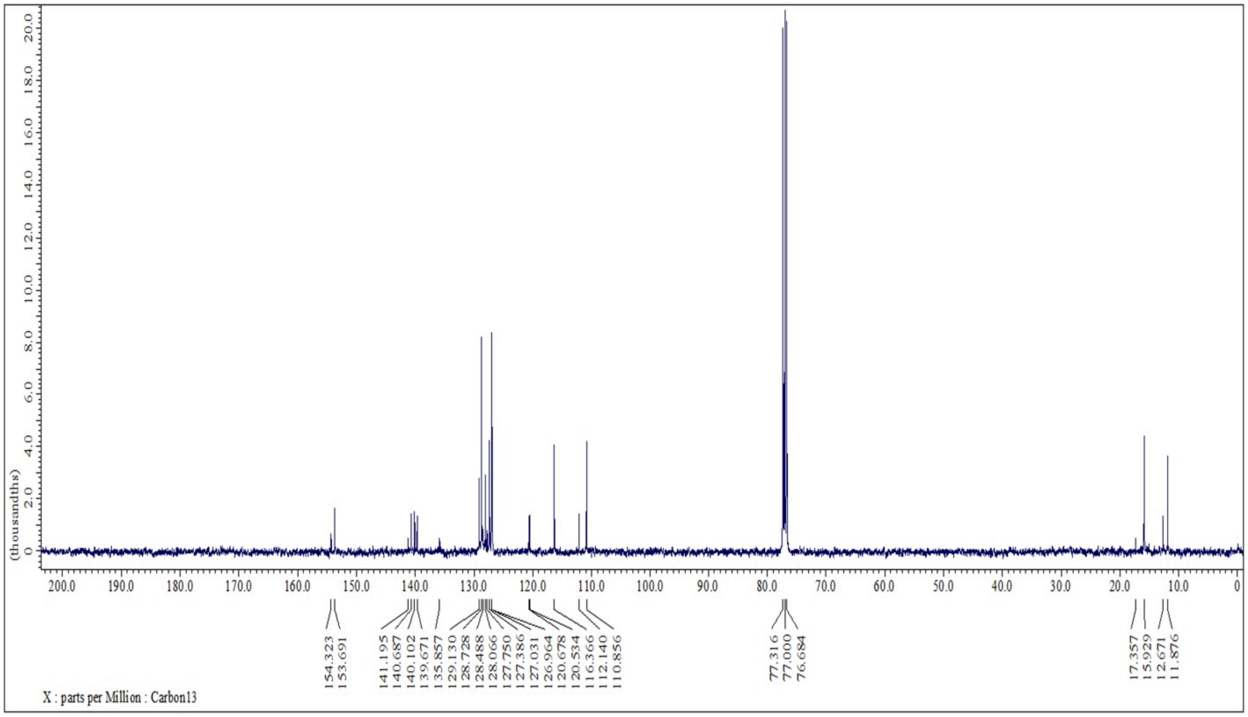
(thousandths)
X : parts per Million : Carbon13
154.323
153.691
141.195
140.687
140.102
139.671
135.857
129.130
128.728
128.488
128.066
127.750
127.386
127.031
126.964
120.678
120.534
116.366
112.140
110.856
77.316
77.000
76.684
17.357
15.929
12.671
11.876

5g. 4-methoxy-6'-(methylthio)-[1,1':4',1''-terphenyl]-2'-ol: ^{1}H and ^{13}C NMR

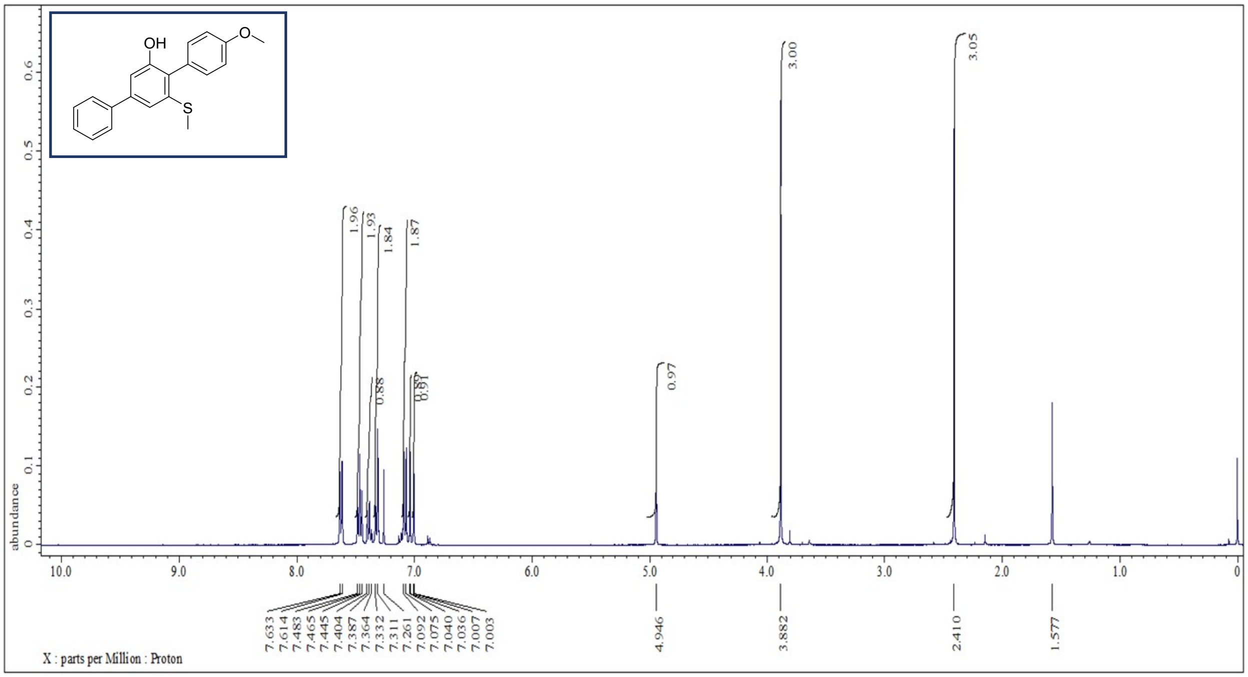

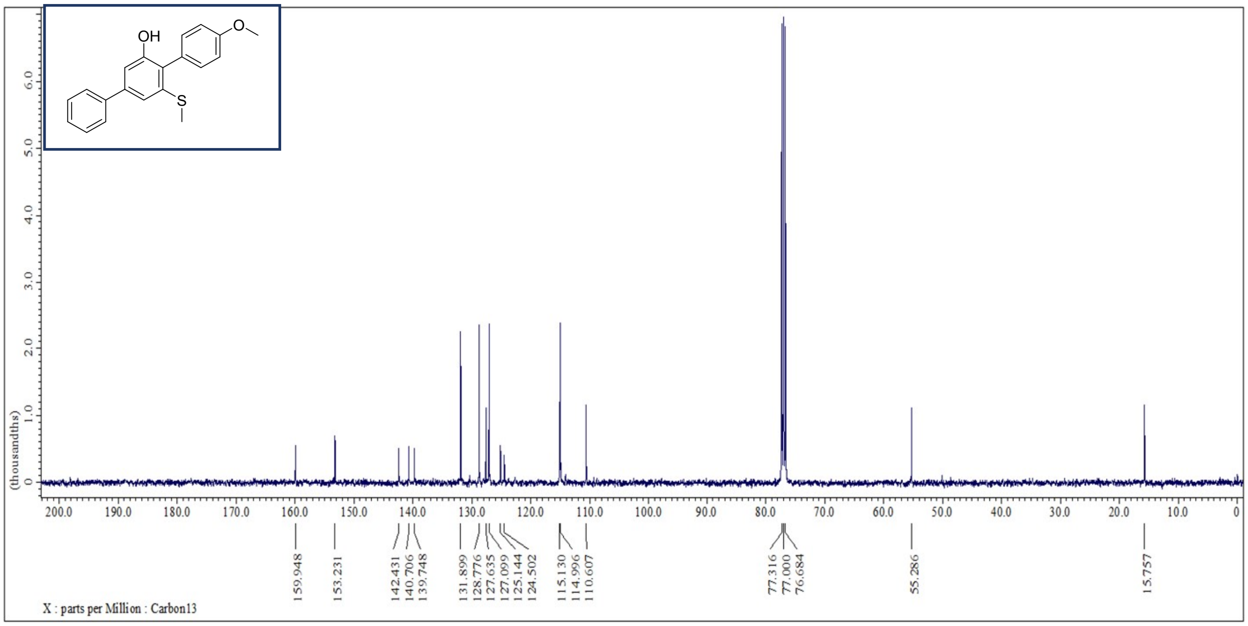

5h. 4,4''-dimethoxy-6'-(methylthio)-[1,1':4',1''-terphenyl]-2'-ol: ^{1}H and ^{13}C NMR

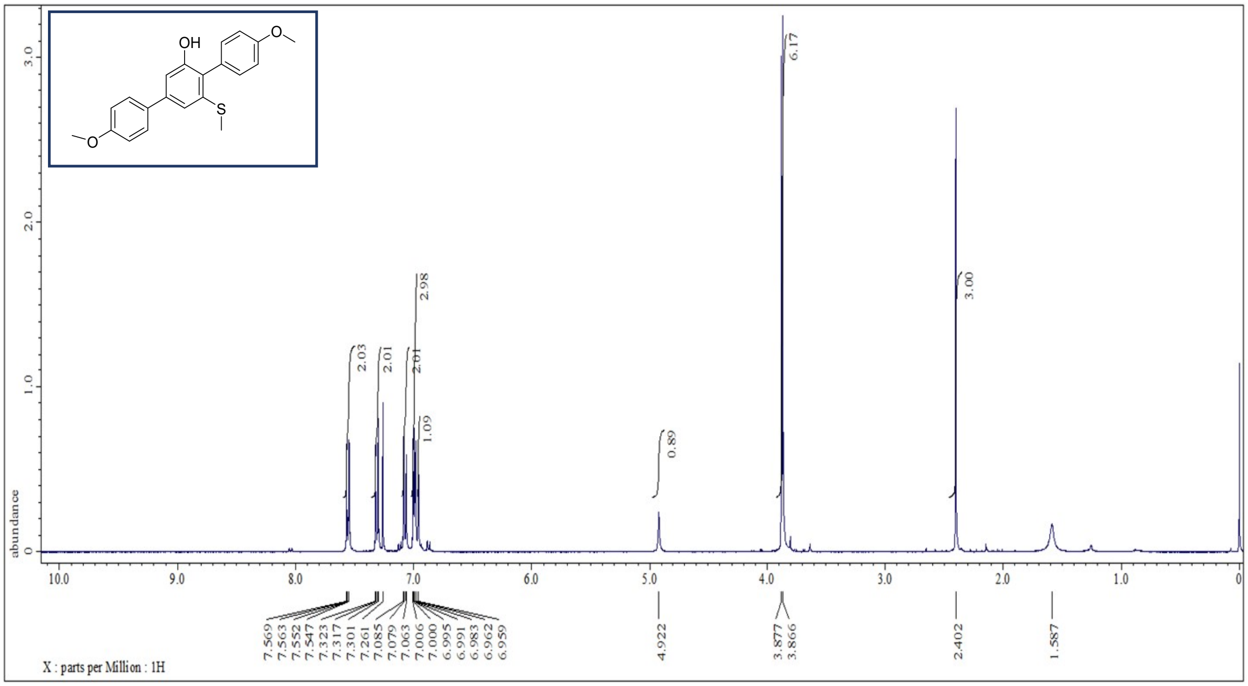

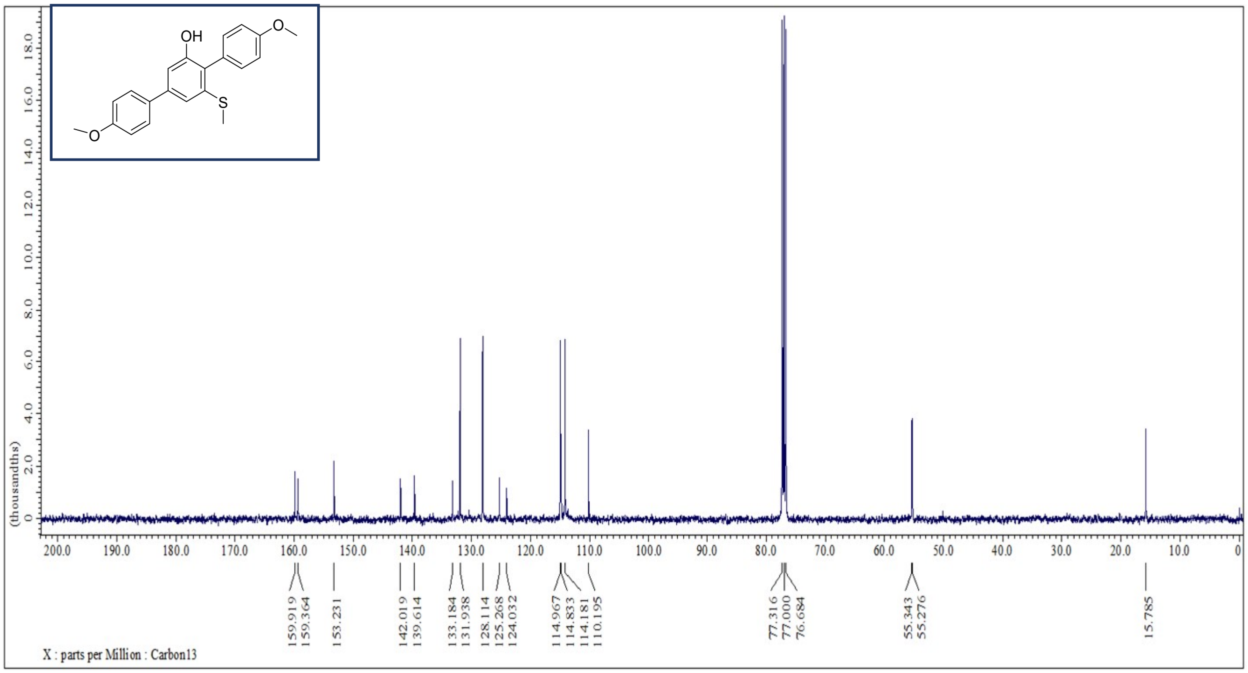

5i. 4'-methoxy-6-(methylthio)-4-(naphthalen-2-yl)-[1,1'-biphenyl]-2-ol: ^{1}H and ^{13}C NMR

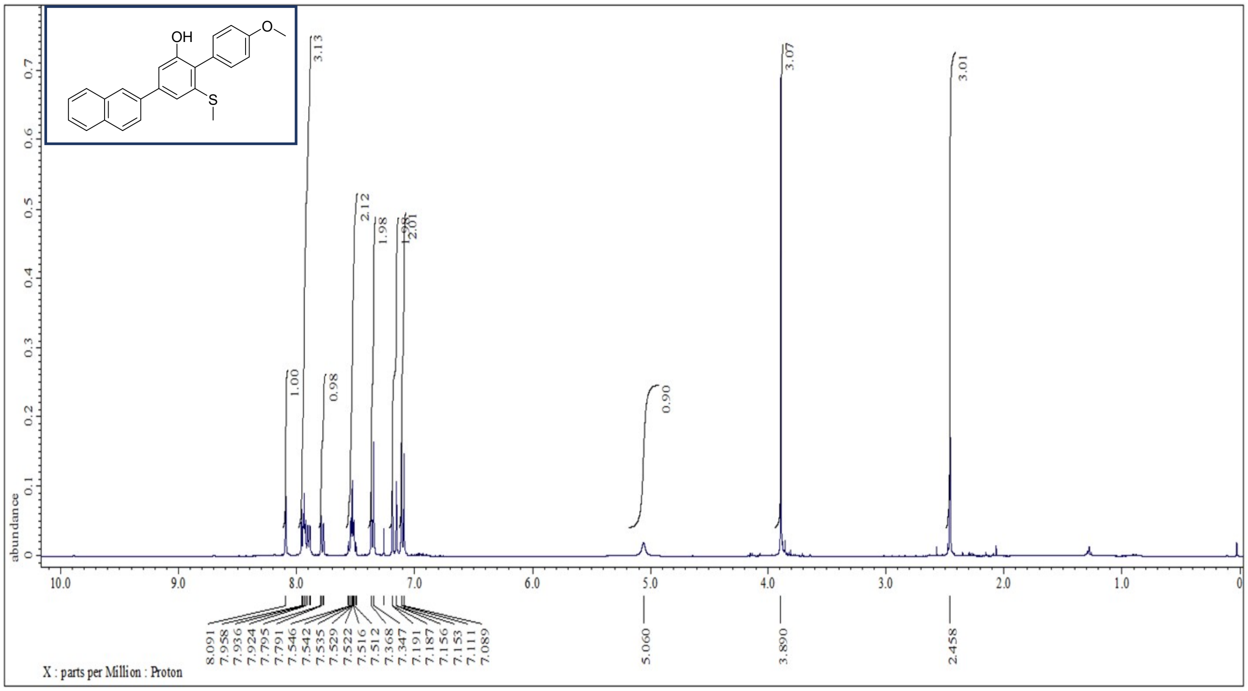

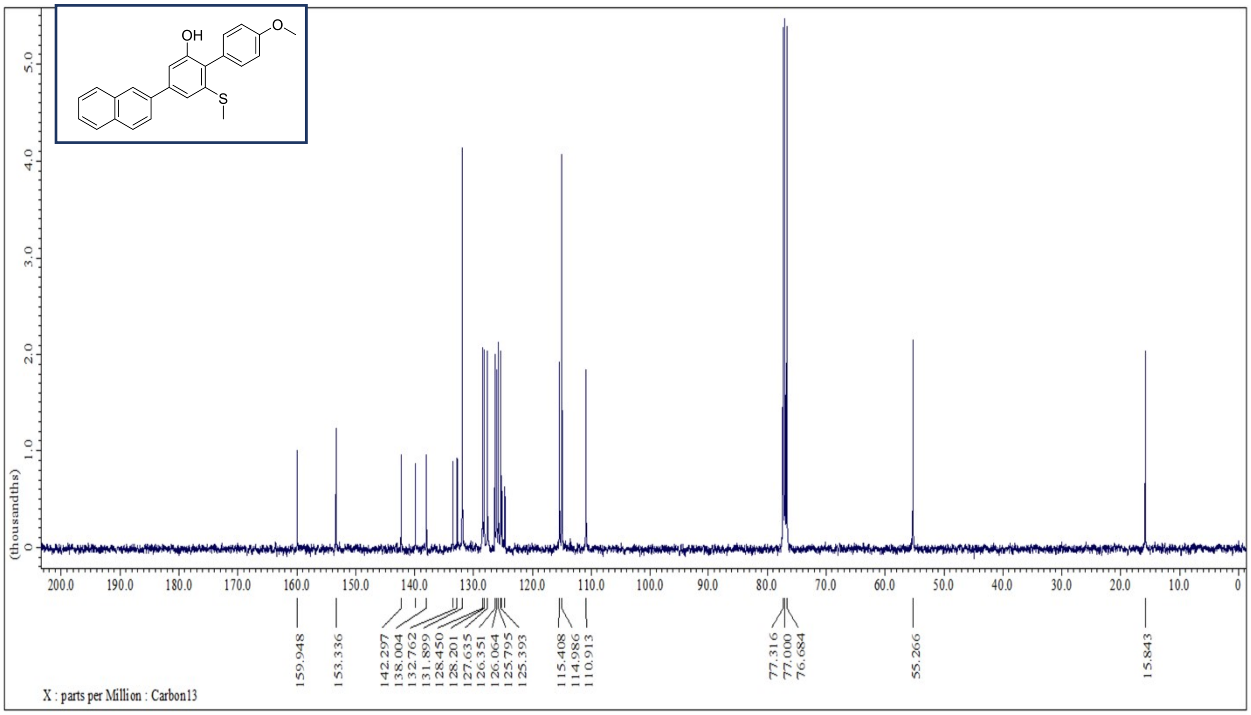

5j. 4-(furan-2-yl)-4'-methoxy-6-(methylthio)-[1,1'-biphenyl]-2-ol: ^{1}H and ^{13}C NMR

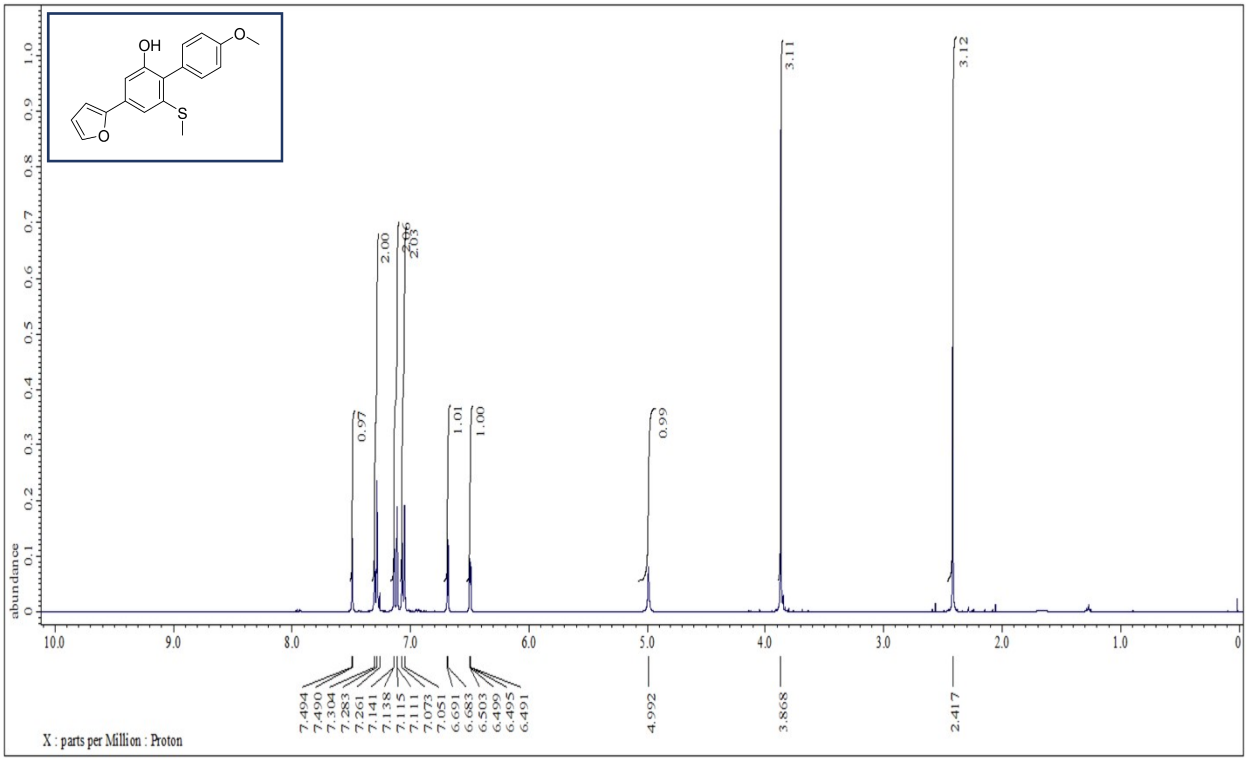

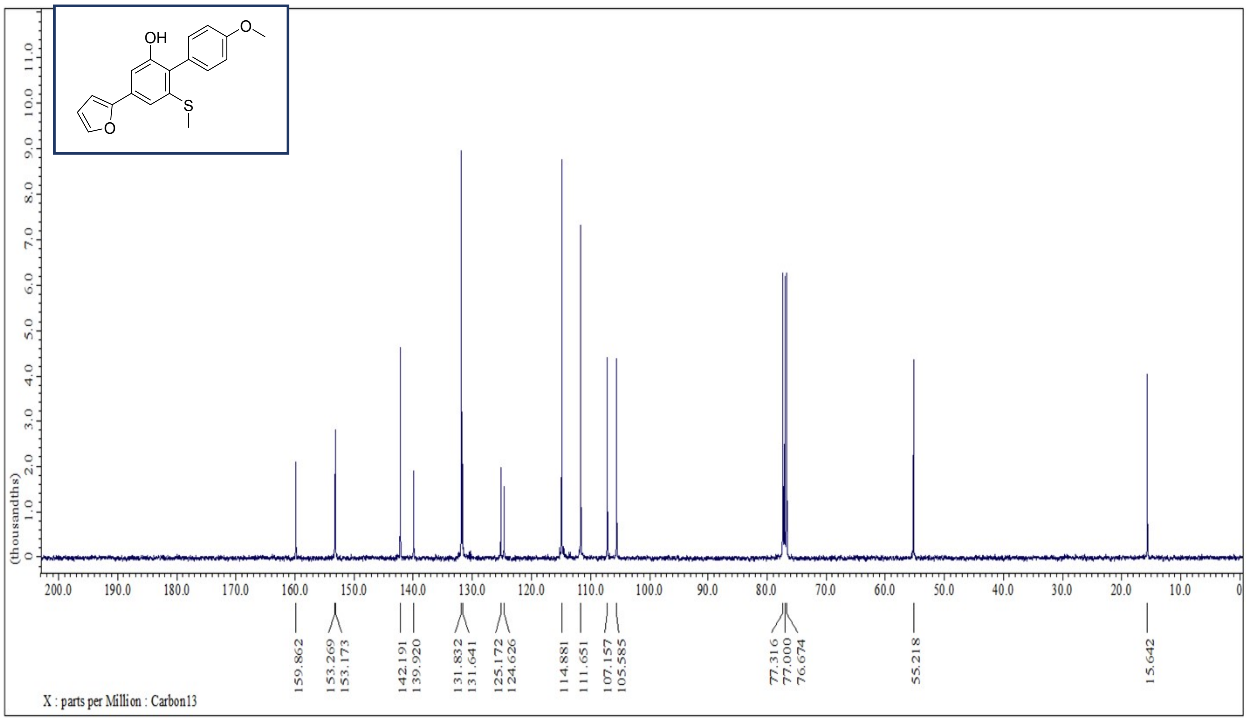

5k. 5'-(methylthio)-4'-phenyl-[1,1':2',1''-terphenyl]-3'-ol: ^{1}H and ^{13}C NMR

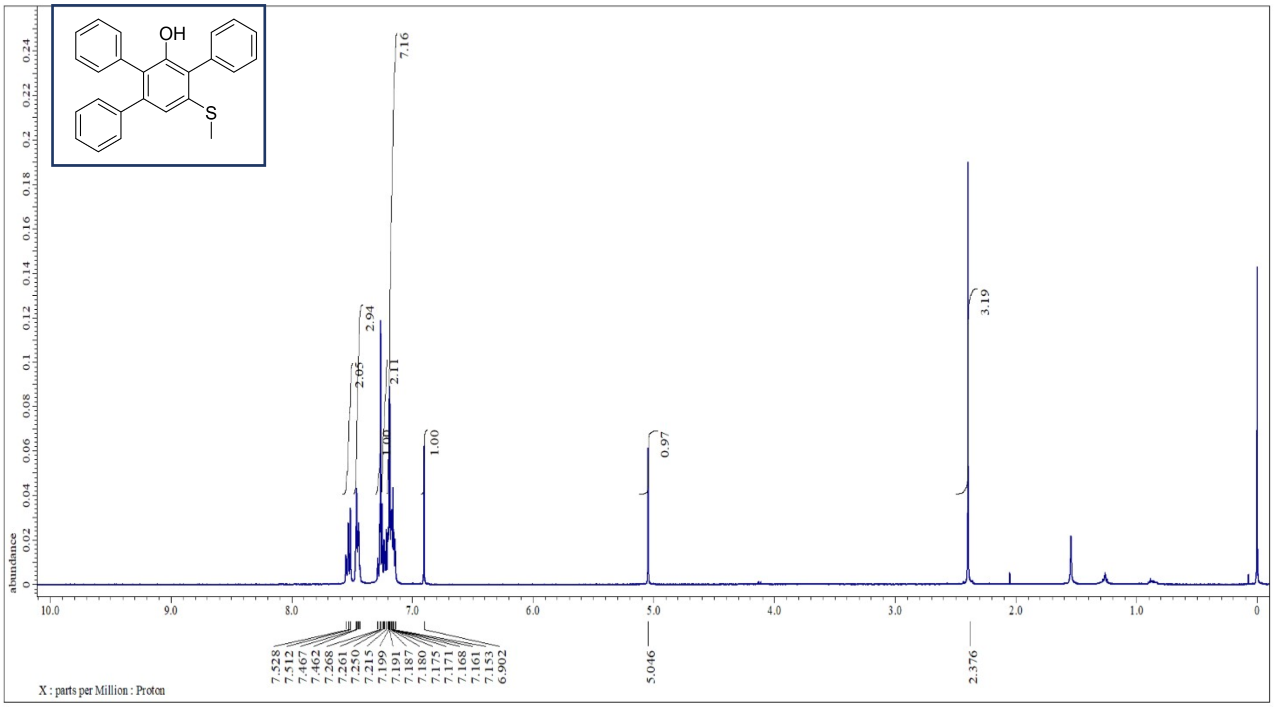

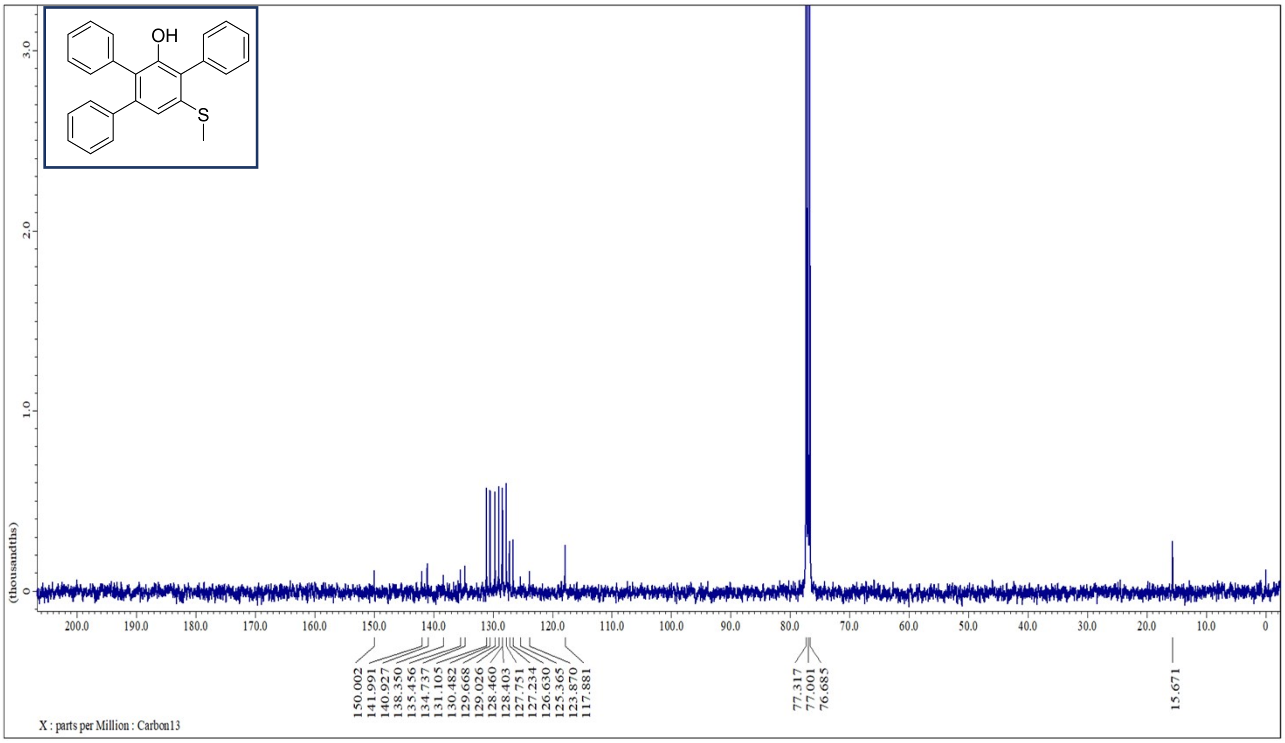

6a. 1-(methylthio)-9,10-dihydrophenanthren-3-ol: ^{1}H and ^{13}C NMR

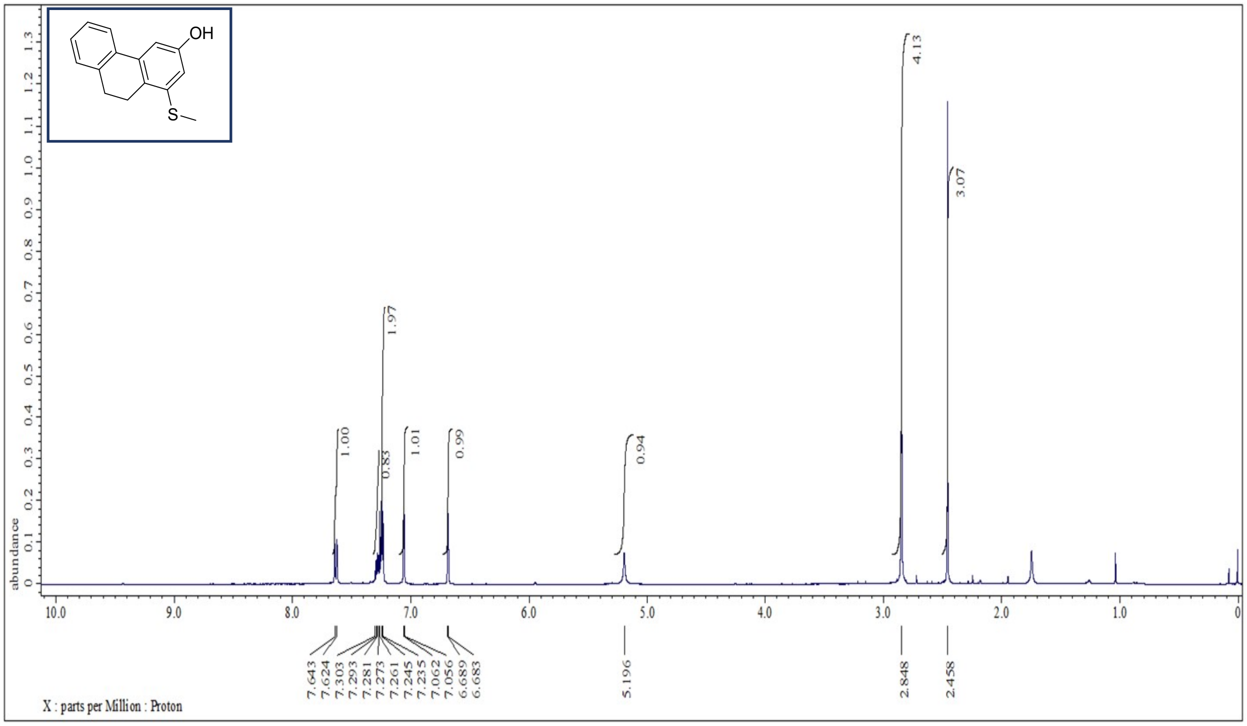

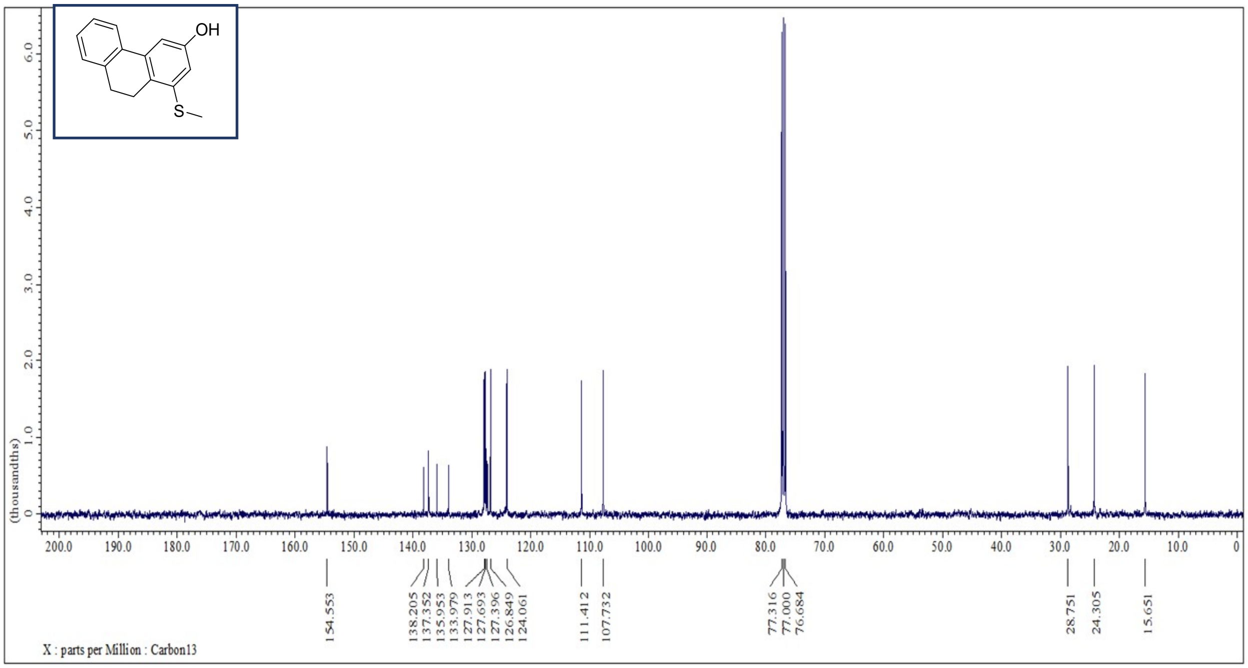

6b. 2,4-dimethyl-1-(methylthio)-9,10-dihydrophenanthren-3-ol: ^{1}H and ^{13}C NMR

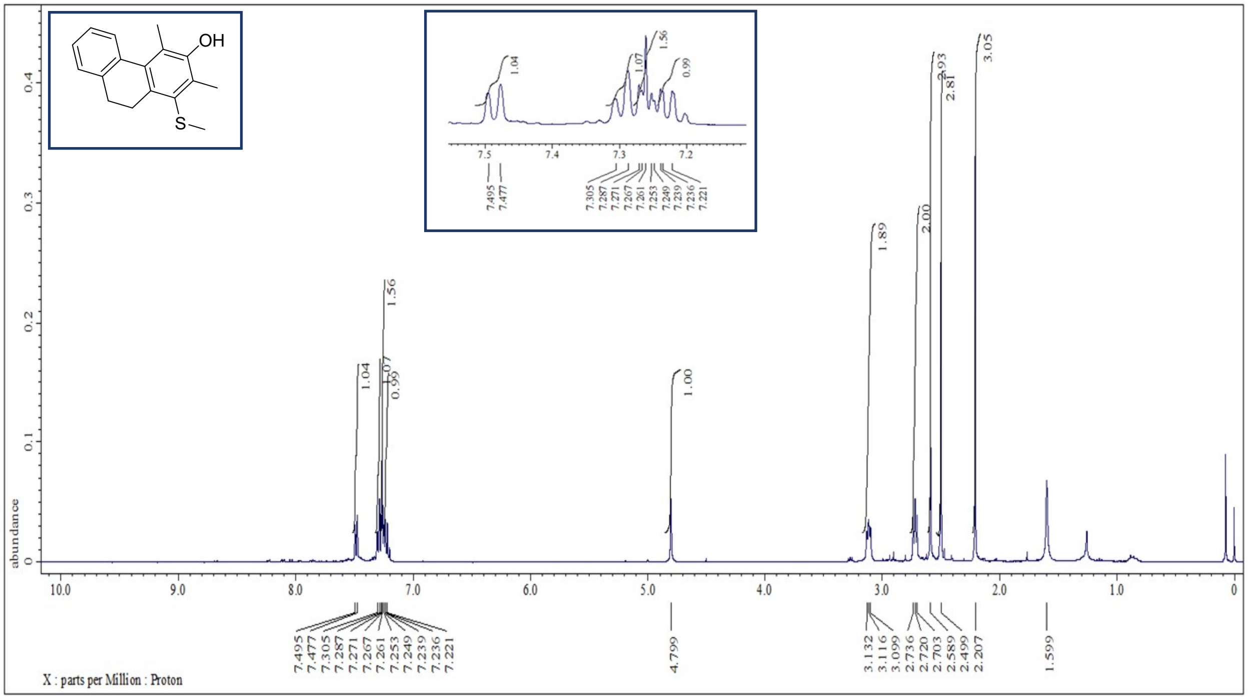

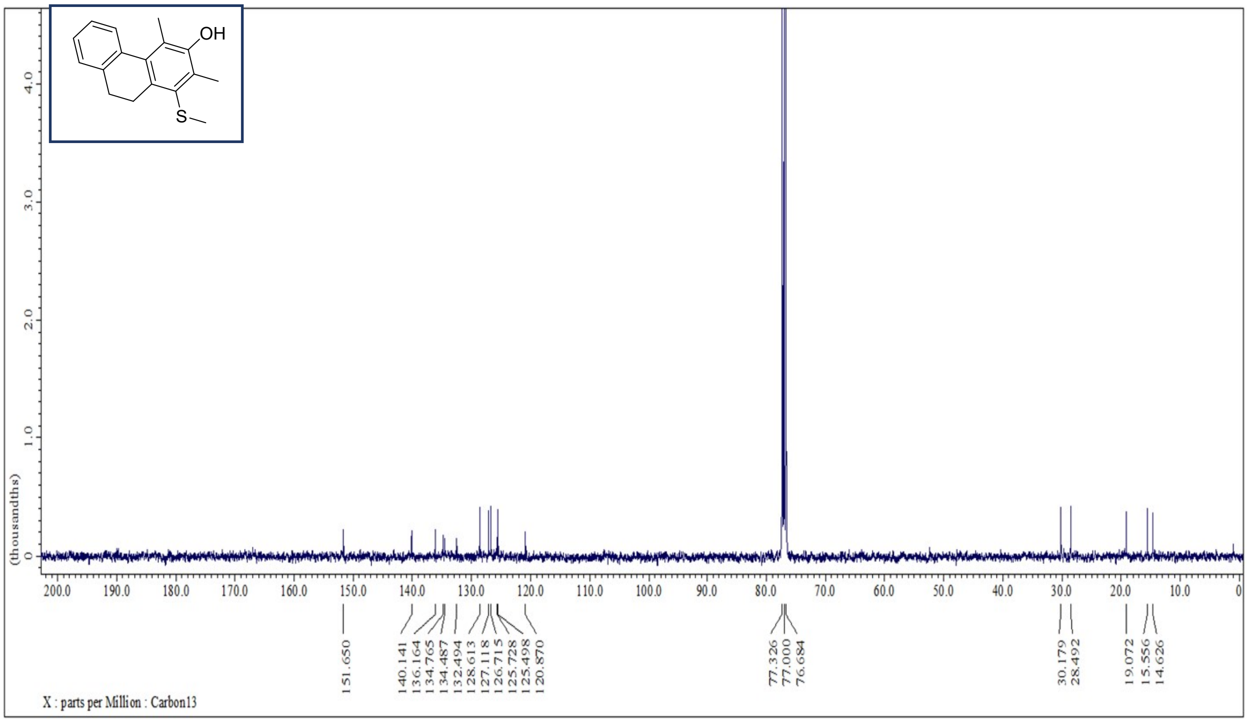

6c. 2-(4-methoxyphenyl)-1-(methylthio)-9,10-dihydrophenanthren-3-ol: ^{1}H and ^{13}C NMR

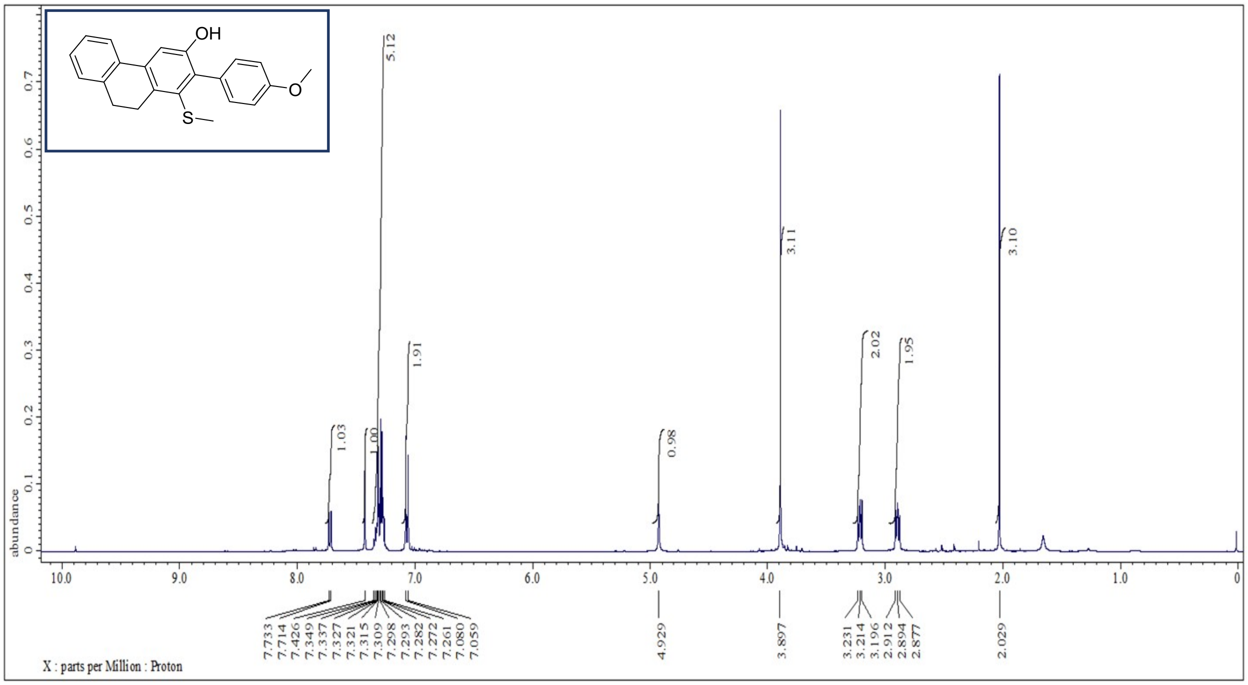

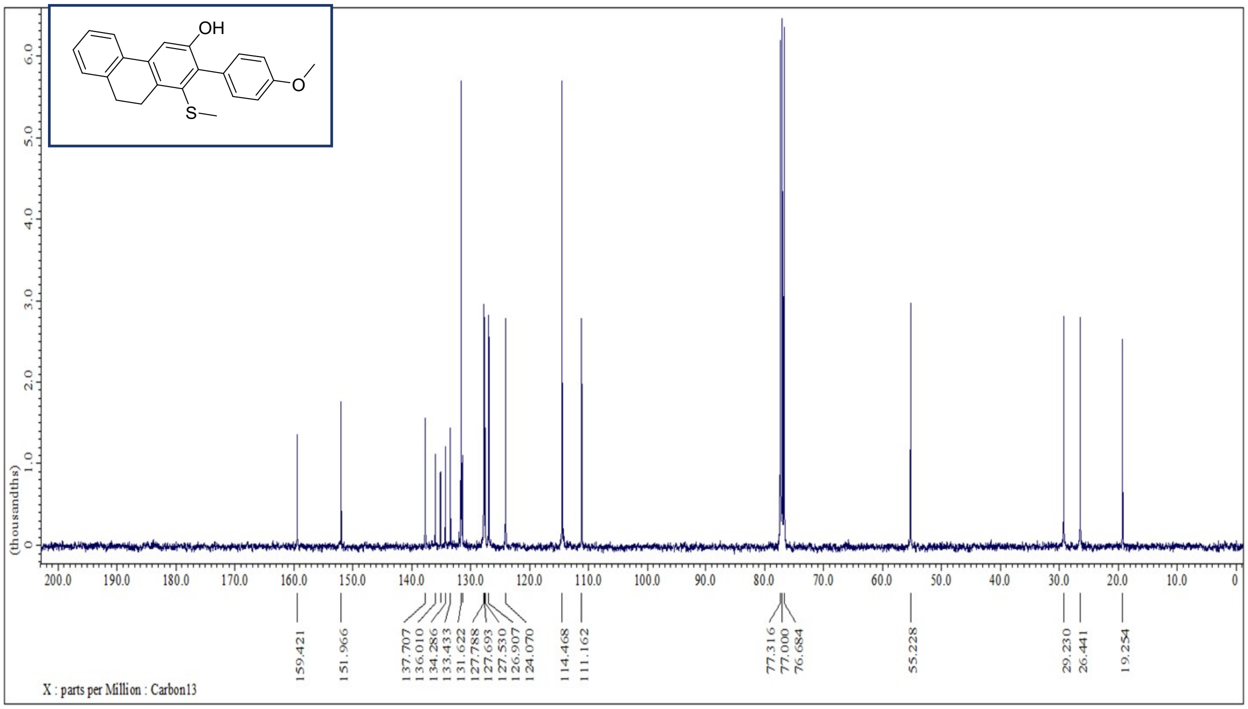

6d. 1-(methylthio)-2,4-diphenyl-9,10-dihydrophenanthren-3-ol: ^{1}H and ^{13}C NMR

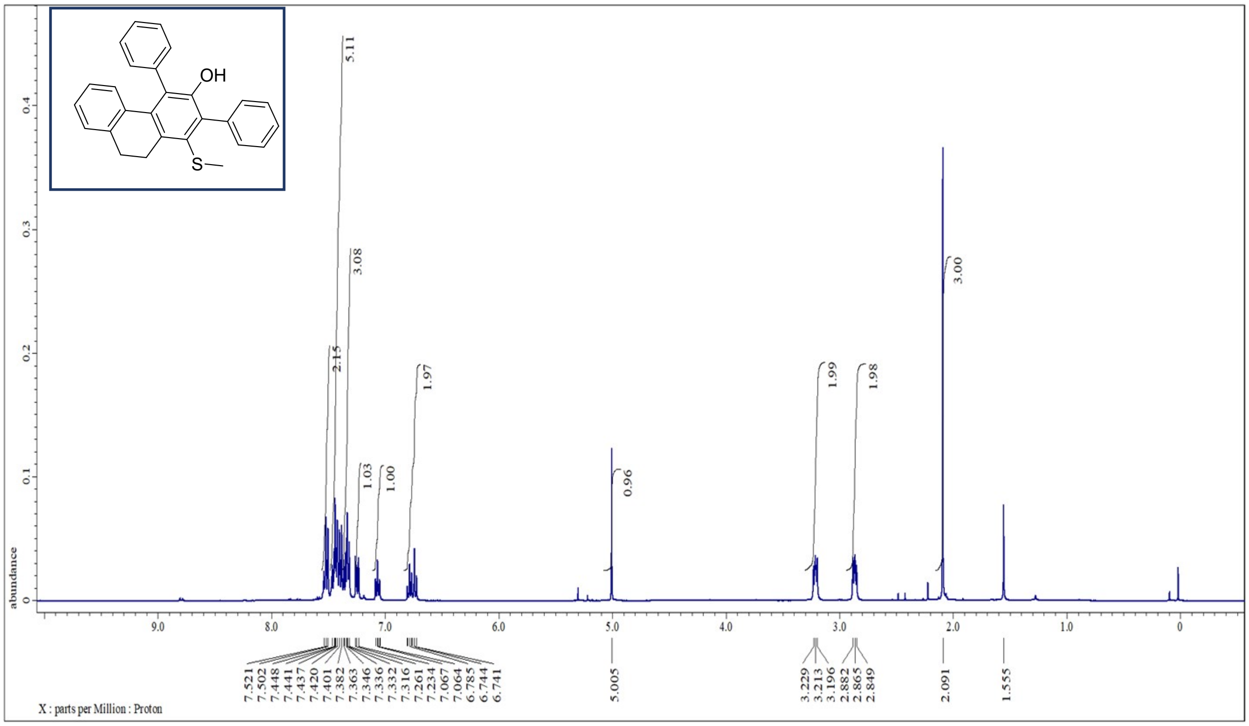

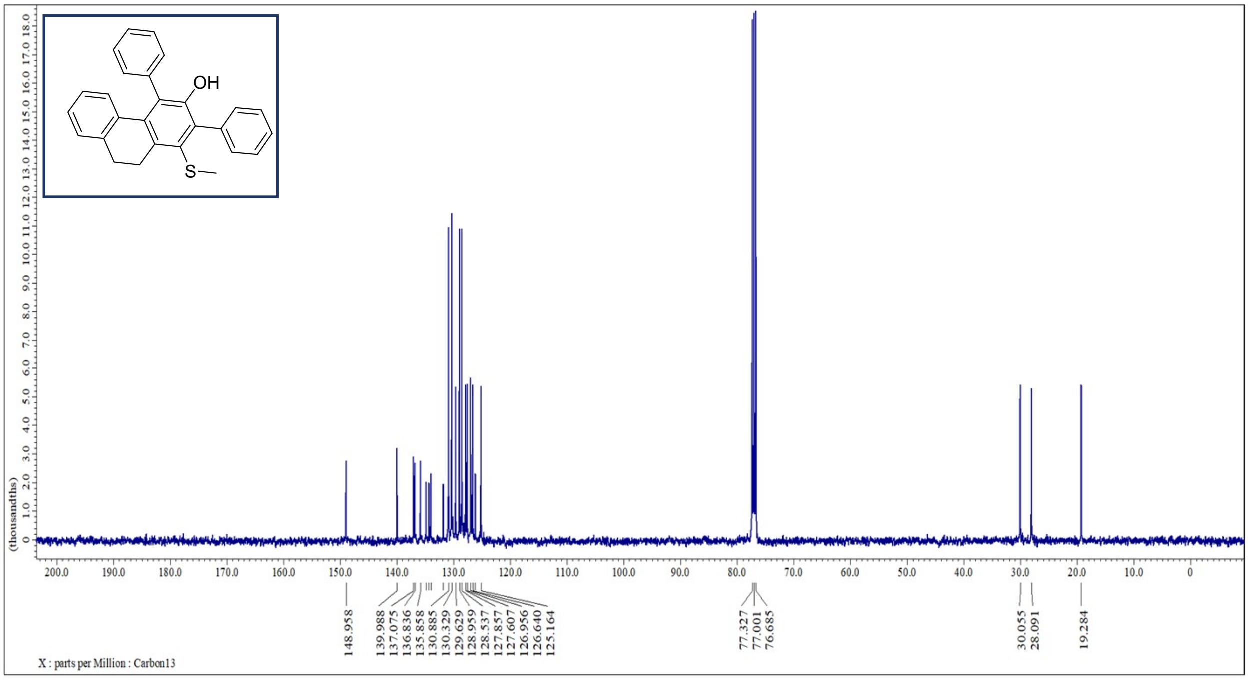

6e. 7-Methoxy-1-(methylthio)-9,10-dihydrophenanthren-3-ol: ^{1}H and ^{13}C NMR

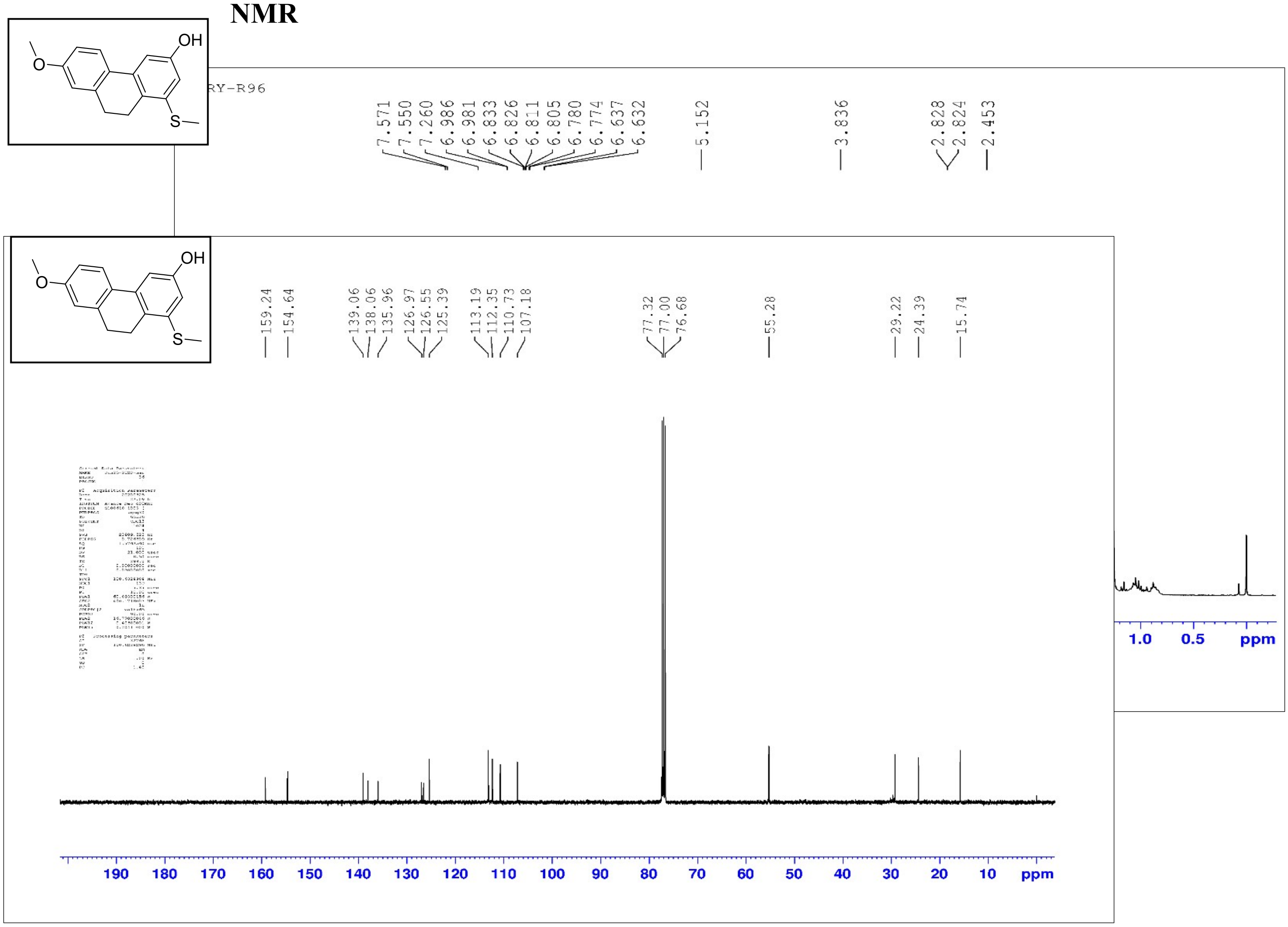

6f. 7-Methoxy-2-(4-methoxyphenyl)-1-(methylthio)-9,10-dihydrophenanthren-3-ol: ^{1}H and ^{13}C NMR

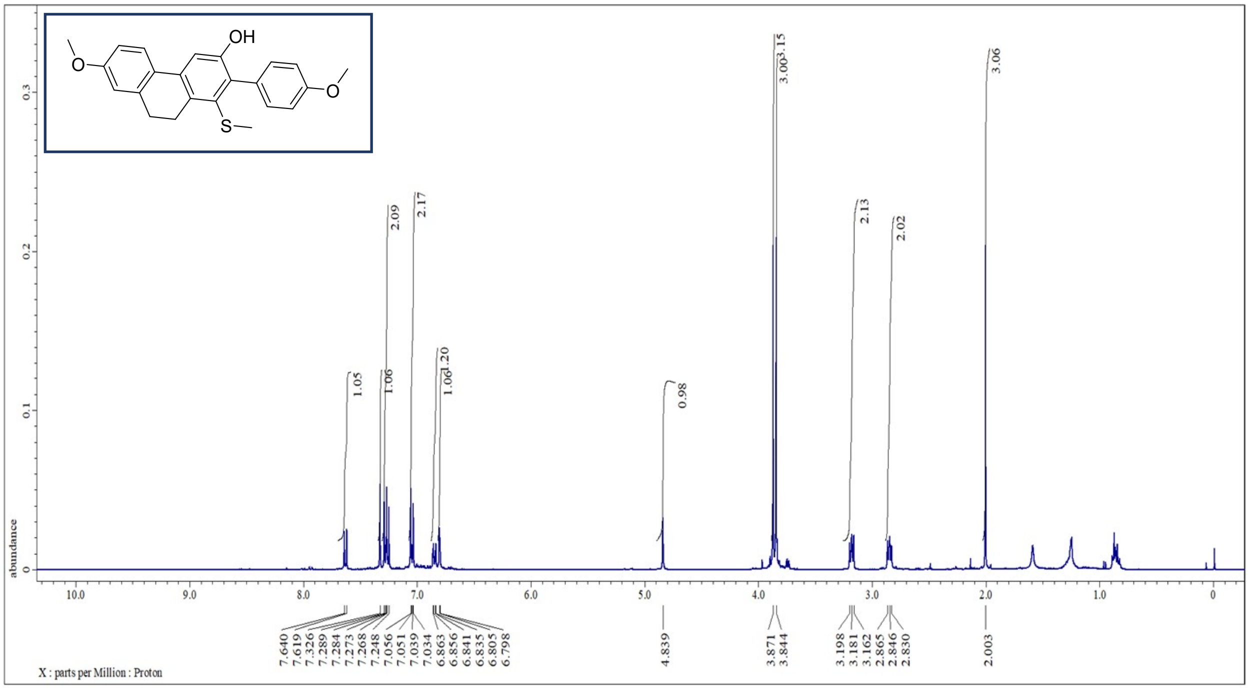

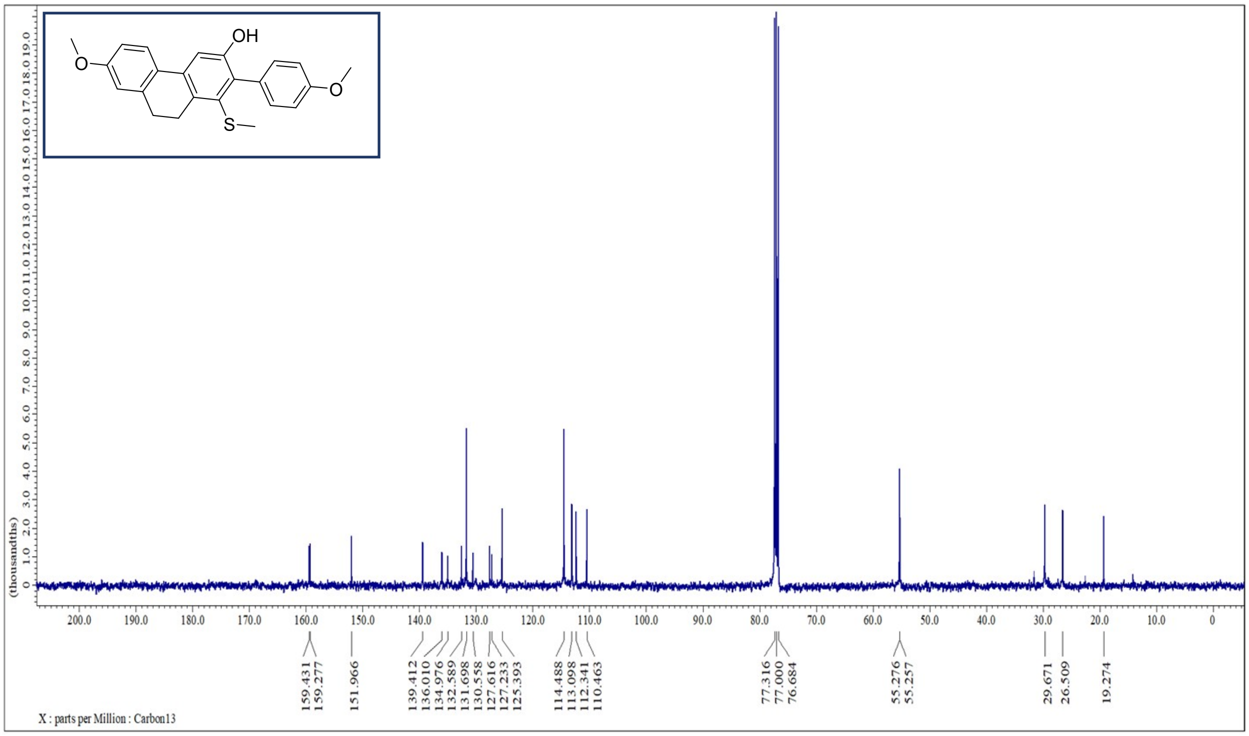

7. 3-cyclopropyl-5-(methylthio)phenol: ^{1}H and ^{13}C NMR

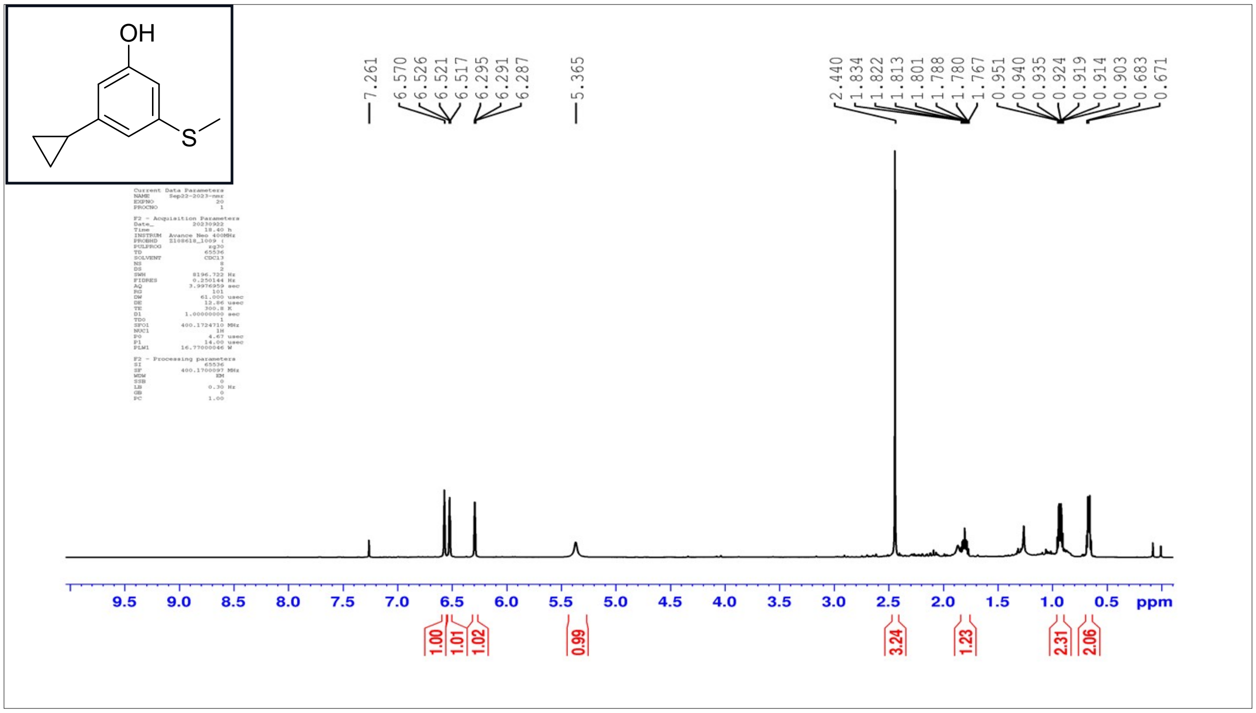
OH
S
7.261
6.570
6.526
6.521
6.517
6.295
6.291
6.287
5.365
2.440
1.834
1.822
1.813
1.801
1.788
1.780
1.767
0.951
0.940
0.935
0.924
0.919
0.914
0.903
0.683
0.671
9.5 9.0 8.5 8.0 7.5 7.0 6.5 6.0 5.5 5.0 4.5 4.0 3.5 3.0 2.5 2.0 1.5 1.0 0.5 ppm
1.00
1.01
1.02
0.99
3.24
1.23
2.31
2.06

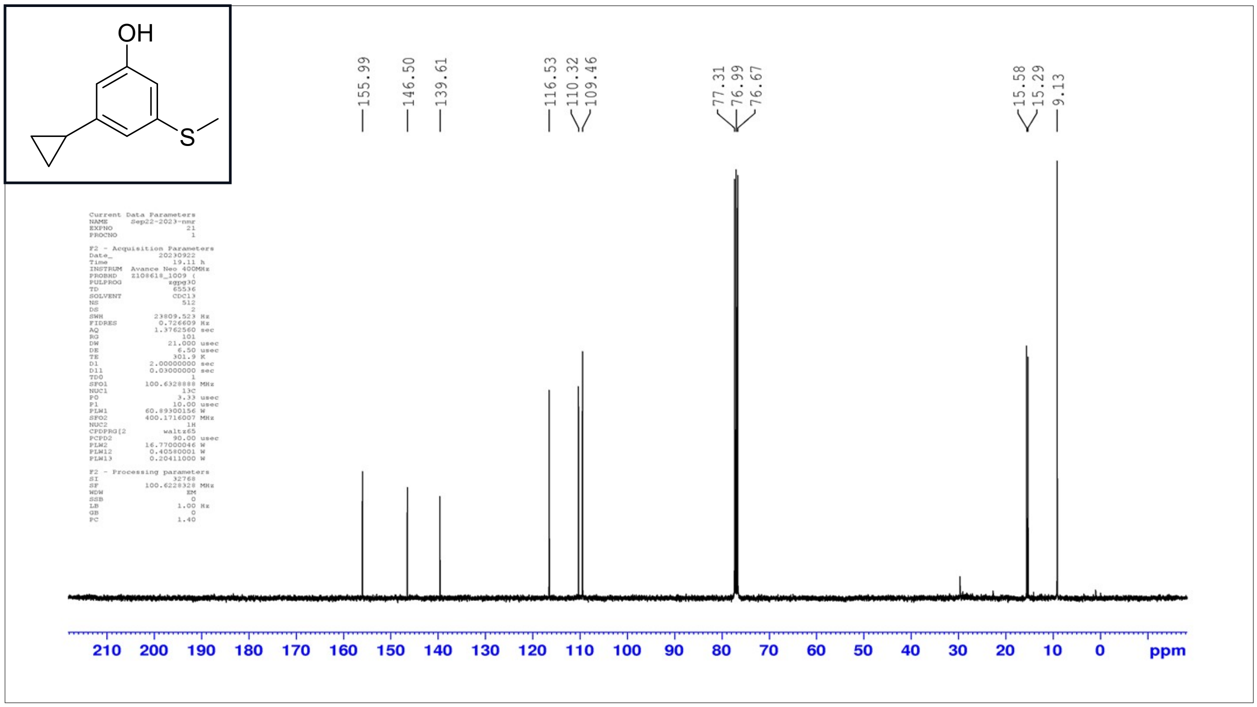
OH
S
155.99
146.50
139.61
116.53
110.32
109.46
77.31
76.99
76.67
15.58
15.29
9.13
210 200 190 180 170 160 150 140 130 120 110 100 90 80 70 60 50 40 30 20 10 0 ppm
